# Evolutionary and structural analyses of alpha-papillomavirus capsid proteins yields novel insights into L2 structure and interaction with L1

**DOI:** 10.1186/1743-422X-5-150

**Published:** 2008-12-17

**Authors:** John Lowe, Debasis Panda, Suzanne Rose, Ty Jensen, Willie A Hughes, For Yue Tso, Peter C Angeletti

**Affiliations:** 1 School of Biological Sciences, Nebraska Center for Virology, University of Nebraska-Lincoln, Lincoln, NE 68583-0900, USA; 2 Veterinary Biomedical Sciences, Nebraska Center for Virology, University of Nebraska-Lincoln, Lincoln, NE 68583-0900, USA

## Abstract

**Background:**

PVs (PV) are small, non-enveloped, double-stranded DNA viruses that have been identified as the primary etiological agent for cervical cancer and their potential for malignant transformation in mucosal tissue has a large impact on public health. The PV family Papillomaviridae is organized into multiple genus based on sequential parsimony, host range, tissue tropism, and histology. We focused this analysis on the late gene products, major (L1) and minor (L2) capsid proteins from the family Papillomaviridae genus Alpha-papillomavirus. Alpha-PVs preferentially infect oral and anogenital mucosa of humans and primates with varied risk of oncogenic transformation. Development of evolutionary associations between PVs will likely provide novel information to assist in clarifying the currently elusive relationship between PV and its microenvironment (i.e., the single infected cell) and macro environment (i.e., the skin tissue). We attempt to identify the regions of the major capsid proteins as well as minor capsid proteins of alpha-papillomavirus that have been evolutionarily conserved, and define regions that are under constant selective pressure with respect to the entire family of viruses.

**Results:**

This analysis shows the loops of L1 are in fact the most variable regions among the alpha-PVs. We also identify regions of L2, involved in interaction with L1, as evolutionarily conserved among the members of alpha- PVs. Finally, a predicted three-dimensional model was generated to further elucidate probable aspects of the L1 and L2 interaction.

## Background

Papillomaviruses (PVs) are small, non-enveloped, double-stranded DNA viruses identified as the primary etiological agent in cervical cancer and their potential for malignant transformation in mucosal tissue is a major health concern. Papillomaviruses (PVs) have also been linked to benign cutaneous lesions and with some non-melanoma skin cancers. These viruses are very common pathogens of epithelial surfaces that account for a variety of proliferating lesions in humans and animals. In the past few years, the available number of complete HPV genomic sequences has increased substantially to comprise more than 150 GenBank entries (2007).

The PV family *Papillomaviridae *is organized into multiple genera based on sequential parsimony, host range, tissue tropism, and histology. We focused this analysis on the late gene products, major (L1) and minor (L2) capsid proteins of the family *Papillomaviridae *genus *Alpha-papillomavirus*. Alpha-PVs preferentially infect oral and anogenital mucosa of humans and primates with varied risk of oncogenic transformation. Two members of the genus *Alpha-papillomavirus *are also associated with cutaneous lesions, Human papillomavirus (HPV) 2 and HPV10. The alpha genus includes twelve cutaneous HPV types and two simian PVs [1]. HPVs of the alpha genus are also sub-categorized based on associated risk of oncogenic transformation into Low Risk (LR) and High Risk (HR) genotypes. The HR group includes 19 HPV genotypes (types 16, 18, 26, 31, 33, 35, 39, 45, 51, 52, 53, 56, 58, 59, 66, 68a, 73, 82, 82subtype) and 13 are grouped as LR (types 6, 6a, 6b, 11, 40, 42, 43, 44, 54, 61, 70, 72 and 81) according to epidemiological evidence [2].

Infection with HR HPV genotypes such as HPV 16 and HPV 18 has been directly related to the subsequent development of cervical cancer [3,4].

PV genomes are characterized by eight well-defined open reading frames (ORFs), which are all transcribed from the same DNA strand and orientation. The translated proteins are classified as "early" (E) or "late" (L) based on their temporal expression. The viral ORFs include 3 regulatory genes involved in transcription and replication (E1, E2, and E4), 3 oncogenes (E5, E6, and E7), and 2 genes encoding for self-assembling proteins which constitute the viral capsid (L1 and L2) [5]. PV capsids are approximately 600 A° (50 nm) in diameter and composed of 72 pentameric capsomeres arranged in a T = 7 icosahedral lattices [6]. The PV capsid proteins L1 and L2 are synthesized late in the infection cycle and function to encapsidate the closed circular double-stranded DNA mini-chromosome [7]. The 72 viral capsomeres are composed of L1 protein pentamers, and the capsomeres are associated with 12 or more copies of the L2 protein. Recombinant L1 or L1 and L2 can be generated in a variety of expression systems to produce self-assembled virus-like particles (VLPs), which approximate the structure of native virions [8,9]. The structure of "small," T = 1 VLPs assembled from HPV16 L1 expressed in *Escherichia coli *has recently been resolved at a resolution of 3.5-A° [10]. Moreover, the crystal structure of L1 major capsid protein provides insights into the conformation of neutralizing epitopes, potential receptor binding sites, the nature of inter-capsomeric contacts [6] and interactions with L2. High levels of neutralizing antibodies can be generated after immunization with HPV L1 VLPs producing highly type-specific neutralization activity [11,12].

Conformational epitopes and the location of epitopes are critical for the production of neutralizing antibodies [13,14]. It has been suggested that L1 loops extending toward the outer surface of the capsomere contain type specific epitopes [6]. Studies with monoclonal antibodies suggest epitopes composed of FG and HI loops are important for HPV 16 [15] neutralization whereas BC, DE, and HI loops are important for neutralization of HPV 6 and 11 [16]. It has also been recently reported that different PV types display distinct features on their surfaces [11]. Analysis of the HPV 11 L1 protein implicated the C-terminus in both DNA binding, as well as inter-capsomere binding [17]. However, less is known about other alpha-PVs. Within a virion, L2 forms contacts with the viral genome, in addition to contacts with L1 pentamers functioning to encapsidate the genome [18]. Comparison of HPV L2 with the polyomavirus major and minor capsid protein suggests that L2 may interact with residues located within the central cavity of L1 pentamer [19]. The carboxy-terminal 44 amino acid region of L2 has been shown to facilitate the interaction of L2 with L1 [19]. Among these 44 amino acids, residues 413–419 are important, since they contain conserved proline residues. It was further demonstrated that heterologous L1-L2 complexes for some PVs can be produced inside the bacteria.

Taking these facts into consideration, we hypothesized that the interaction domains of L1 and L2 should be fairly conserved among alpha- PVs. Some L2 domains may be exposed at the virion surface, thus enabling recognition of a specific epitope for immune recognition [20,21]. This surface-exposed region of L2 would also be able to interact with cellular receptors to facilitate uptake of virions [22]. Moreover, it has been suggested with bovine papillomavirus (BPV) that L2, amino acids 61–123 are exposed on the surface of the virion and can be recognized by monoclonal antibodies while the majority of the residues appear to be buried inside the surface [23].

The association of HPVs with benign and malignant neoplasia has led to research efforts focused toward improvement on the current understanding of diversity within this virus group, so that diagnosis, treatment, and control of HPV infections may be optimized. Many aspects about evolution of PVs are still relatively poorly understood. Therefore, probing of evolutionary and structural relationships between PVs will likely provide novel insight to assist in clarifying the functional differences between PVs and their tropic microenvironment; cutaneous cells or mucosal epithelial cells. To date, a broad range of bioinformatics tools have been applied to analyze the complete PV genome (or at least properly alignable genomic regions). In this paper we identify the regions of the major capsid proteins as well as minor capsid proteins of alpha-papillomavirus that have been evolutionarily conserved, and define regions that are under constant selective pressure with respect to the entire family of viruses. Here we show that the loops of L1 are, in fact, the most variable regions among the alpha-PVs. We also identify regions of L2 involved in interaction with L1 as evolutionarily conserved among the members of alpha-PVs. Finally, we generated a predicted three-dimensional model to further elucidate probable aspects of the L1 and L2 interaction.

## Methods

### Alpha-papillomaviruses

The Seventy-six alpha-PV sequences obtained for this analysis were retrieved from the NCBI protein database according to the reference list of alpha-PVs published on the Universal Virus Database [24]. In addition to the list of PV species in the alpha genus provided by ICTV we also included six characterized variant HPV-16 sequences in this analysis. Corresponding GenBank accession numbers are included in the additional data file [See Additional file 1].

### Alignment

The compiled protein sequence sets were aligned using MUltiple Sequence Comparison by Log-Expectation, MUSCLE [25,26]. MUSCLE alignment was selected as it has been shown to be one of the most accurate multiple alignment tools currently available [26]. MUSCLE utilizes a 3-stage algorithm 1. Generate a progressive alignment 2. Increase the accuracy of the progressive alignment by reconstructing a tree with the Kimura matrix and the clustering method 3. Iterative refinement of progressive alignment

MUSCLE outputs were then loaded into the CLUSTALX user interface for graphical representation of residue conservation and analysis [27]. Sequence logo representation of MUSCLE alignments were generated using WebLogo 3 [28]. The complete output of the L1 and L2 alignments can be viewed in the Additional Data file [See Additional file 1].

### 3D prediction of L2 and L1-L2 interaction

The HPV16 L1 protein structure was obtained from the RCSB Protein Data Bank [29]. The secondary structure of HPV16 L2 protein was predicted by submission of the L2 amino acid sequence into 3D-Jigsaw [30] and these data refined using the Swiss Model Server . The L2 amino acid sequence data was then submitted to SAM-T09, Sequence Alignment and Modeling System, for tertiary structure prediction [31-34]. The SAM predicted L2 structure was then further refined using AL2TS to predict side chains [35]. HPV16 L1 protein structure and the N-terminus of the L2 predicted structure were then submitted to ClusPro, a Protein-Protein Docking Web Server [36-40]. The L1 PDB crystal structure and predicted L2 structure were submitted as ligand and receptor, respectively. PyMOL, a molecular visualization program, was used to view and manipulate both the predicted L2 model and the predicted protein-protein interaction models of HPV16 L1-L2.

## Results

### Variable regions coincide with surface loops of L1 protein

**Figure 1:**
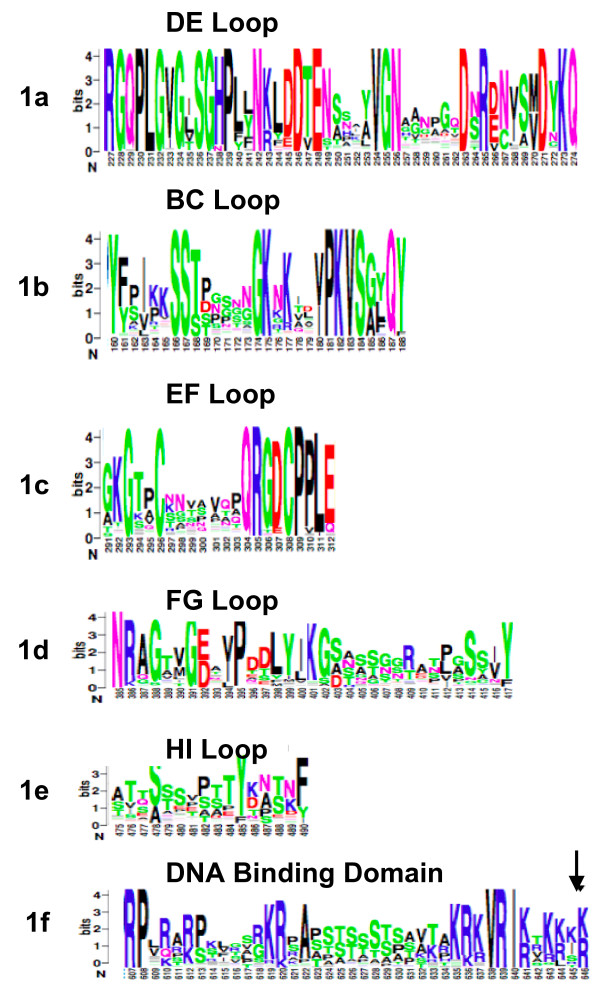
**Analysis of conserved regions within the L1 protein**. Seventy-six alpha-HPV L1 sequences were obtained from the NCBI protein database under the Universal Virus Database, as well as six known HPV-16 L1 variant sequences provided by the ICTV. The sequences were aligned using the MUltiple Sequence Comparison by Log-Expectation (MUSCLE). MUSCLE outputs were loaded into CLUSTALX user interface for graphical representation of residue conservation and analysis. Sequence conservation is by the height of residue logos (indicated in bits), as generated by WebLogo 3. The consensus sequences resulting from the alignments of the external loop regions are as follows: DE loop (aa 227–274) (1a), BC (aa 160–188) (1b), EF loop (aa 291–312) (1c), FG loop (aa 385–417) (1d), the HI loop (aa 475–490) (1e) antigenic determinants, and the conserved DNA binding regions (1f). The HPV 16 cysteines at residues 201 and 454, which are involved with disulfide linkages, are conserved across the entire alpha-papillomavirus family.

We found the external loop regions of alpha-PVs correlate to the least conserved regions in our alignment (Figure 1). The external loop regions: DC loop (AA 50–69), DE loop (AA 110–153), EF loop (AA 160–189) and FG loop (AA 262–291) and the HI loop (AA 348–360) have been characterized as being antigenic in the HPV16 model [15]. The regions which have been previously characterized as showing antigenicity, and have characterized monoclonal antibodies, are L1 residues F50, 1–173, 111–130, A266, 268–281 and 427–445 [19,20]. It has been suggested that these regions are less conserved than other L1 regions because they are under constant immunogenic selective pressure. Our sequence analysis of L1 shows high degree of similarity among all the genotypes [1,2] [Additional file 1]. Despite being classified into different genotypes, identical variable regions are clearly present within the HPV L1 protein (Fig. 1).

### L1 conserved cysteines and lysines

HPV 16 cysteine 201 and 454 are conserved across the entire alpha-papillomavirus family alignment (Fig. 1). This is in good agreement with previous studies that found these regions were required for interaction between the L1 monomers to form trimers [18]. These trimers are believed to be required to form the capsomer, and thus the virion. There are also three lysines residues (278, 356 and 361) that are moderately conserved and highly conserved when viewed from the fact that in each alpha-PV, at least one of these three residues was a lysine. It has also been shown that these residues are involved in cellular binding to host heparan sulfate chains [14].

### Conserved C-terminus DNA binding region

**Figure 2:**
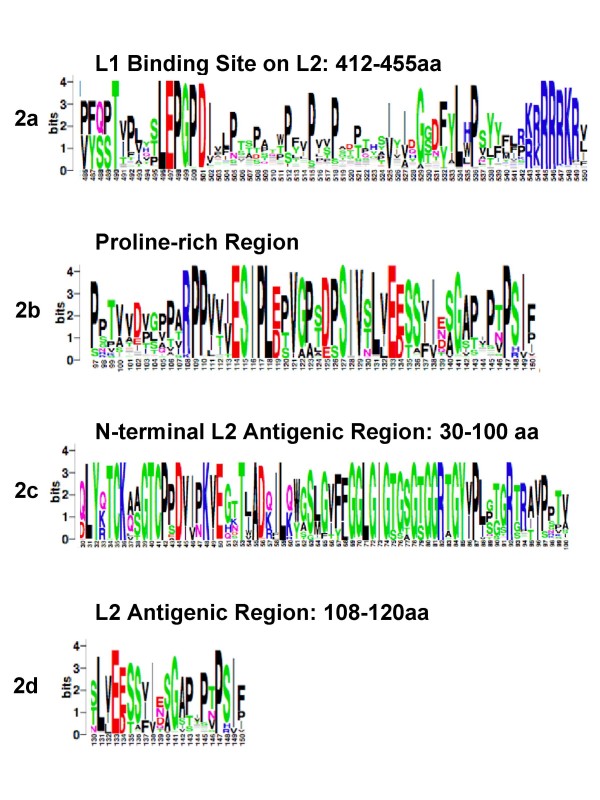
**Analysis of conserved regions within the L2protein**. Similar to the L1 analysis, L2 protein sequences derived from NCBI and ICTV databases were aligned using MUSCLE and visualized with CLUSTALX interface for graphical representation of residue conservation and analysis. Sequence conservation is by the height of residue logos (indicated in bits), as generated by WebLogo 3. The known L1 interaction domain of L2 (486–550 aa in the alignment, corresponding to 412–455 aa in HPV16 L2) (2a), conserved proline rich regions (2b), well conserved N-terminal antigenic regions (30–100 aa) (2c), and (aa 108–120) (2d) are shown. The C-terminal DNA binding domain, rich in lysines, from HPV 16 AA 500–531, is highly conserved for alpha HPVs.

Our alignment shows that the C-terminal DNA binding domain, rich in lysines, from HPV 16 AA 500–531, is highly conserved for alpha-PVs (Fig. 2). The specific location of lysines in the sequence is somewhat variable especially upstream away from the C-terminus. At the extreme C-terminus there are almost completely lysine residues, which are conserved across the alpha-PV family.

### H4 helix region is conserved

The H4 region (AA 413–428, [19]) is in a region of conservation with 5 amino acids being universally conserved (414L, 418Y, 419R, 425A, and 428C4) and four being close to universal (413T, 416D, 420F and 421L). This conservation is, mostly but not wholly, at the N terminus side of the helix.

### L1 regions of interaction with L2 conserved

**Figure 3:**
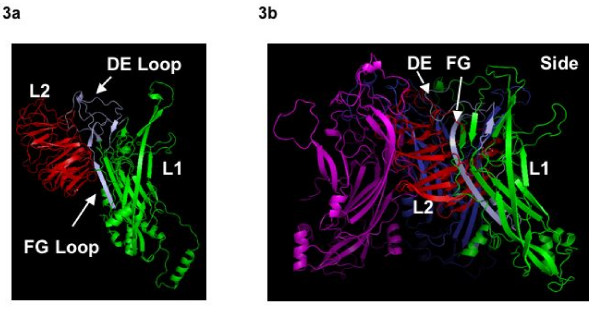
**Predicted 3D model of the L1:L2 interaction**. The HPV16 L1 protein structure was obtained from the RCSB Protein Data Bank. The secondary structure of HPV16 L2 protein was predicted with using the 3D-Jigsaw and the Swiss Modelling Server . The data was then analyzed with the SAM-T09 program (Sequence Alignment and Modeling System, for tertiary structure prediction) which was further refined using AL2TS. The docking position of L2 to L1 was predicted with ClusPro, (Protein-Protein Docking Web Server). The L1 and L2 structures were then visualized using PyMOL, (a molecular visualization program). The predicted L2 structure in its docking position on the L1 monomer (3a). The predicted orientation of the L2 protein within the L1 pentamer structure; two L1 monomers of L1 have been removed to clearly show the alignment of L2 within the structure (3b).

Upon analysis of the 3d docking prediction between L1 and L2, we targeted regions of L1 that were in prime positions to be involved in the protein interaction with L2, specifically the interaction in the region of 247–269 and the region of 113 to 130 (Fig. 3). These regions have a fair amount of conservation (supplemental Fig.), which is probably due to the protein interaction being critical for infectious virion formation.

### L1 interaction domain of L2 is highly conserved

We analyzed the L1 carboxy-terminal binding domain of L2 among alpha-PVs. We observed a moderate degree of conservation exists for these domains among alpha-PVs. Interestingly, proline residues are conserved in many genotypes and occur frequently in this region compared to other regions of the L2 protein. L2 is hypothesized to have at least two L1 interaction domains and the second domain has been suggested to be located in the N terminal portion of L2. Our results show that such repetitive proline residues are not highly conserved in the amino-terminal portion of L2, but to some extent the repetitive proline motifs are found in region corresponding with HVP 16 aa 97–150 (Fig. 2a &2b). The alpha-papillomavirus L2 alignment results did not verify a conserved amino acid region corresponding to the hypothesized second amino terminal L1 interaction domain of L2 as found in BPV-1.

### N-terminal L1 binding domain of L2

We attempted to identify possible conserved neutralizing epitope domains of L2 that would provide valuable direction for development of cross-protective therapeutics against alpha-PVs. Our data suggests residues corresponding to HPV 16 aa108–120 are moderately conserved and a specific subset of 8 residues are highly conserved (Fig. 2d). Other domains of L2 responsible for neutralizing antibody response have been suggested corresponding to amino-terminal 88 residues [41] more specifically 17–36 amino acid region might be responsible for neutralizing antibody response [42,43]. Our alignment shows the amino-terminal residues are mostly conserved among alpha-PVs. Two alignments of alpha-PVs, grouped into high risk and low risk, depicted a similar pattern of conservation at amino terminus as well as for the residues corresponding to HPV 16 L2 aa 108–120.

### DNA binding domains of L2

Positively charged arginine and lysine residues of the extreme carboxy-terminus DNA binding domain of L2 appear highly conserved among alpha-PVs (Fig. 2a). The evolutionarily conservation of the L2 amino-terminus including the DNA binding domain suggests the function of DNA binding for capsid formation and viral DNA transport upon cellular entry has remained relatively stable over the divergence of these PVs.

### Predicted 3D model of the L1:L2 interaction

Data from the predicted secondary structure of HPV16 L2 was compared with the tertiary structure model of L2 confirming similarity. The L1 binding sites on L2 were confirmed to be within the N-terminal region. Specific interactions predicted between L1 and L2 include the DE loop and the FG loop of L1 and specific proline-rich regions of L2 (Fig. 3). Amino acids 105–120 within the L1 DE loop interact with one highly conserved and one completely conserved proline within L2 at amino acids 53–59. The FG loop of L1, consisting of amino acids 247–269, also is predicted to interact with one highly conserved and one completely conserved proline of L2. These regions of L2 consist of residues 24–30 and 260–264. These prolines range from highly conserved to completely conserved among all alpha-PVs. Based on the protein-protein interaction model of the L1 and L2 monomers, we conclude that L2 binds within the center of the L1 pentamer. The position of the L2 antigenic region, therefore, is predicted to face outward when bound to the L1 pentamer. (Fig. 3)

## Discussion

Analysis of the alpha-PV family L1 and L2 proteins provided evolutionary information to assist in understanding the predicted interaction domains and their roles in virion assembly. Particularly of value is our L2:L1 structural interaction model, which has similarities with the manner in which Polyomavirus VP2 interacts with VP1 [44].

Analysis of the sequence alignments, suggested that the variable regions of L1 are mainly located within the surface loops and comprise several neutralizing epitopes. Numerous groups have identified neutralizing epitopes within the L1 surface loops, strongly suggesting that these regions are the major targets for neutralizing antibodies [14-16,36,45-49]. Conversely, only a few CTL epitopes have been identified within L1 protein and targeting of these CTL epitopes could be linked to individual HLA allele expression [50-52]. Indeed, our sequence analysis indicated a strong correlation between these immune epitopes and variable regions of L1 (Fig. 1). We conclude that immune selection as the main driving force for diversity of surface loops on HPV L1 protein, but that the overall structures of the loops are conserved. It is possible that there are other, yet to be identified, epitopes downstream of the HI loop, as our analysis shows that some of these regions are relatively variable (Supplemental Fig.). The caveat to our analysis is that we had only compared linear epitopes with variable regions, as data on discontinuous epitopes is unavailable. It is conceivable that some variable regions could also comprise of discontinuous epitopes. Nevertheless, comparison and identification of L1 protein variable regions could provide beneficial information for development of broadly neutralizing antibodies against HPV.

Along with the interaction loops, several other features or regions of HPV L1 are relatively conserved within the multiple alignments. There are also 3 lysine amino acids (278, 356 and 361) that are thought to bind to heparan sulfate side chains on the cell surface and facilitate cellular entry. Mutations of these residues to alanine is known to cause a reduction cell binding and infection of pseudovirions [53]. Residue 278 is within the FG loop, while the other two are contained in the HI loop. These residues are somewhat well conserved in alpha-papillomavirus family. With residue 361 being the highest conservation and 356 being the lowest and not very well conserved. There is most likely an evolutionary selective pressure to change these loops and looking at the amino acid conservation it appears that all of these amino acids occur at or right next to regions of low conservation. This presumably is due to the selective pressure placed on the antigenic loops by the host adaptive immunity. The function of cellular binding and entry to the cell is absolutely required for viral replication and existence, and so there should be selective pressure to maintain amino acids that are required for cell entry. If these residues are indeed important for cellular binding and entry, then these residues are probably experiencing both of these pressures and this may be an explanation of why some are less conserved than others, while each sequence tested have at least one lysine at one of these positions. The results of the previous experiments [53] suggest that there is an additive effect with these residues, suggesting that they may not all be at the same selective pressure, which would support the idea that alpha-PVs can withstand some changes in these residues.

Also there is a region, the H4 helix that is thought to be involved in pentamer-pentamer interaction [6]. H4 is the helix that is thought to be on the outer rim, deep within the pentamer interaction. It is the most distal part of the protein. Deleting this region causes loss of interaction between pentamers, however the pentamers still form. This region, while not being the best conserved, contains 5 amino acids that are universally conserved in the alpha-PV family. These amino acids are probably critically important in the pentamer interaction, and may constitute conserved interaction points.

We have shown that the conserved region where the final 11 AA of the C-terminus are involved in DNA interaction [54] is fairly well conserved across the genomes, albeit not exactly the same residues positions along the sequence, but the region is holistically conserved (Fig. 1f). Most likely this region is involved in packaging DNA into the virion. Since this is universally needs to be accomplished between alpha-PVs, conservation of this region is probably evolutionally favored.

We found the carboxy terminal L1 binding domain of L2 to be conserved among the alpha-PVs irrespective of high or low risk group. However, the structural interaction of L1 and L2 and formation of capsid is still not clear. Minor capsid protein (L2) binds the L1 capsomers but not to the VLP, suggesting that L2 co-assembles with L1 rather than being inserted into a pre-formed capsid [19]. L2 is required for efficient genome encapsidation, suggesting the capsid assembles around histone-bound genome rather than by injection of the genome into the capsid via a portal vertex. The involvement of L2 in genome encapsidation coupled with the DNA-binding properties of L2 suggests that, within a virion, L2 forms multiple contacts with the viral genome in addition to contacts with L1 pentamers [18,55]. Our results show that both DNA binding domains of L2 are highly conserved among alpha-PVs. The level of conservation of the L2 DNA binding domains indicates the maintenance of this binding function has been vital to the virus from an evolutionary standpoint.

Two distinct L1 binding domains have been described for BPV1 L2; a C-terminal L1 binding domain (BPV L2 aa384–460) that interacts with L1 capsomers in vitro, and a central region (BPV L2 aa129–246) that fails to interact with capsomeres [56]. These authors described the interaction between BPV1 L2 aa129–246 and L1 on the basis of co-immunoprecipitation and co-localization studies. However, when we aligned the N-terminal interaction domain of BPV with HPV-16, only 20% similarity was observed. This region is furthermore not conserved among the members of alpha-PVs. Our data revealed that the N-terminal 100–150 amino acids of L1 are moderately conserved among alpha-PVs and there is occurrence of proline residues more frequently than other region of HPV. We hypothesize that this L2 region is likely to contain the second L1 interaction domain. However, further experimental evidence is required to support this hypothesis.

The carboxy-terminus L1 binding domain described between residues 396–439 of HPV11 L2, is consistent with the C-terminal L1 binding domain in residues 384–460 of BPV1 L2 [56]. Our results confirmed that the C-terminal L1 interaction domain of L2 is highly conserved throughout the members of alpha-PVs. It seems that the C-terminus of L2 composed of many hydrophobic residues neutralizes charges on L1 which further leads to changes in conformation in L1, thereby permitting the assembly of T = 1 VLPs at neutral pH. Moreover the assembly of L1 and L2 into full-size T = 7 VLPs at neutral pH may require further modification of the in vitro assembly buffer conditions, different lengths of L2 or a combination of L1 and L1-L2 containing capsomere. For the important mechanism of capsid assembly, PVs have maintained an evolutionarily conserved L1 binding domain at the C-terminus of L2. The location of the primary L1-binding site on the carboxy-terminus of L2, the structural complexity, and hydrophobicity of the L1-L2 interaction have interesting parallels to the mouse polyomavirus VP1-VP2 interface [57]. However a certain degree of difference in capsomere organization between PVs and polyomaviruses exists due to the amino acid variation between theses two viruses [6].

Recently much focus has been given toward the development of potential vaccines against HPV. Anti-L1 antibodies obtained by immunizing mice or rabbits with the L1 capsids have been shown to have primarily type specific neutralizing activity. Limited cross-neutralizing activity has been observed between closely related types such as HPV18 and 45, and HPV6 and HPV11 [58]. Moreover, anti-L1 antibodies can protect animals against challenge with animal PVs [59,60]. The L1 capsids of HPV6, 11, 16, and 18 were used in recent clinical trials as prophylactic vaccines, which successfully induced type-specific neutralizing antibodies in recipients [61,62]. However, there is no general consensus regarding the epitope at the amino terminus of L2 responsible for production of neutralizing antibody response. One group showed amino acids from 108–120 are conserved between HPV 16 and HPV18, which have at least 46% similarity in this region [20]. Our results depict conservation of the first half of this region (aa108–120) among alpha-PVs and this might be the epitope associated with production of neutralizing antibody response. It is important to note that the second half of this region (108–120) is highly variable and the cause of this variability is currently unclear. Other domains of L2 responsible for neutralizing antibody response have been established as well [41,42]. These groups suggested that amino-terminal 88 residue more specifically 17–36 amino acid region might be responsible for neutralizing antibody response. Our results correlated with the previous published results [20,41,63]. When separated and group by HR and LR, the alpha-PVs produced a similar pattern of conservation at the amino terminus as well as for HPV 16 residues 108–120. These results suggest that both regions may be involved in production of neutralization antibody and cross protection against different types. A recent study reported that the amino terminal 18–144 is conserved in some of the papillomavirus and our results are also in good agreements this observation [63]. Furthermore, we show that the extreme N-terminal region is highly conserved for the alpha-PVs. The N-terminal region is also the location of a DNA binding domain and it is still unclear how the N-terminal epitope is exposed on the surface of the virion. Recently Buck et al 2007 a proposed model of assembly for L2 and L1 capsomers suggested there may be changes in conformation of capsid in order to extrude the terminal epitopes.

Several studies have attempted to identify the nature of both neutralizing epitopes of both L1 and L2 using L1/L2 VLP to better define the topology of L2. All these data suggest that HPV16 L2 residues 108–120 and 69–81 are epitopes displayed on the surface of VLPs and virions [20,22]. Clearly our knowledge of L2's topology in the capsid is limited but perhaps the L1 capsomer-L2 complex or pseudovirions might be suitable for X-ray crystallographic studies. Unlike structures of VLPs or capsomers, analysis of pseudovirion or true virion preparations would also clarify the interaction between the capsid and the nucleohistone core. Studies with purified capsid proteins or VLPs indicate that the C-terminal positively charged tail of L1 that includes a nuclear localization signal is also critical for binding to and packaging DNA. Similar sequences on both termini of L2 may also play a role in encapsidation of the viral genome as well as infection. In the present study we attempt to predict the secondary structure of L2. We also mapped the interaction domain of L2 within the monomer of L1. Our data shows that the amino terminus of L2 is involved in interaction with L1. Our data is unique from previous results in which the second independent L1 interaction domain of L2 has been shown to be amino acid 129–246 for BPV [56]. Analysis of the corresponding region of BPV with alpha-PVs we only 20% similarity suggesting that other regions of L2 may be involved in interaction with L1.

Nonetheless, our L2:L1 structural interaction model had distinct similarities with the Polyomavirus VP2 interaction with VP1 [44]. The Polyomavirus VP2 protein is predicted to be inserted at the center of VP1 pentamers, just as we predict PV L2 to be positioned in L1 pentamers. The alignment of L2 for the alpha-PVs, the amino terminus 100–150 aa is rich in proline. Previous studies have also suggested that the proline rich regions are involved in protein-protein interaction [64]. Moreover, the carboxy terminus region of L2 contains repetitive prolines which are highly conserved in the alpha-PVs [19]. However, our computer-predicted L2 structure should be considered a hypothetical. Nevertheless this interaction is representative of L1 and L2 interaction domains. Two large limitations of the predicted 3D interaction model are the absence of DNA bound to L2 and the difficulty in determining L2 flexure within the pentameric form of L1. In this model the DE and FG loop of L1 are involved in interaction with L2 and these loops are also outside the structure. According to one proposed model, L2 drives the formation of capsid by recruiting the L1 pentamers [65,66] and it has been suggested that both the L1 interaction domain of L2 are necessary for efficient virus encapsidation [56]. Studies utilizing VLPs and purified capsid proteins coupled with detailed virion mutagenesis and structural studies are necessary for confirmation of these results.

## Competing interests

The authors declare that they have no competing interests.

## Authors' contributions

JL assembled HPV sequences and performed MUSCLE and Clustal analyses. DP did further data and literature research and helped write the manuscript. SR performed 3D protein modeling and docking analyses. TJ, WAH helped in analysis of 3D structures and conserved regions. FT helped in writing the manuscript and data analysis. PCA conceived of the study and coordinated the work, performed 3D rendering of structures and using Pymol, and edited the manuscript.

## Supplementary Material

Additional file 1 Multiple sequence alignment of L1 and L2 proteins. Seventy-six alpha-HPV L1 and L2 sequences were obtained from the NCBI protein database under the Universal Virus Database, as well as six known HPV-16 L1 and L2 variant sequences provided by the ICTV. The sequences were aligned using the MUltiple Sequence Comparison by Log-Expectation (MUSCLE). MUSCLE outputs were loaded into CLUSTALX user interface for graphical representation of residue conservation and analysis. Sequence conservation is by the height of residue logos (indicated in bits), as generated by WebLogo 3. All outputs for both sequences are shown. Click here for file

## References

[B1] de Villiers EM, Fauquet C, Broker TR, Bernard HU, zur Hausen H (2004). Classification of papillomaviruses. Virology.

[B2] Munoz N, Bosch FX, de Sanjose S, Herrero R, Castellsague X, Shah KV, Snijders PJ, Meijer CJ (2003). Epidemiologic classification of human papillomavirus types associated with cervical cancer. N Engl J Med.

[B3] Cain JM, Howett MK (2000). Preventing cervical cancer. Science.

[B4] Bosch FX, Manos MM, Munoz N, Sherman M, Jansen AM, Peto J, Schiffman MH, Moreno V, Kurman R, Shah KV (1995). Prevalence of human papillomavirus in cervical cancer: a worldwide perspective. International biological study on cervical cancer (IBSCC) Study Group. J Natl Cancer Inst.

[B5] Munger K, Howley PM (2002). Human papillomavirus immortalization and transformation functions. Virus Res.

[B6] Chen XS, Garcea RL, Goldberg I, Casini G, Harrison SC (2000). Structure of small virus-like particles assembled from the L1 protein of human papillomavirus 16. Mol Cell.

[B7] Lowy DR, Howley PM, Knipe DM HP, Griffin DE, Lamb RA, Martin MA, Roizman B, Straus SE (2001). Papillomaviruses. Fields Virology.

[B8] Kirnbauer R, Booy F, Cheng N, Lowy DR, Schiller JT (1992). Papillomavirus L1 major capsid protein self-assembles into virus-like particles that are highly immunogenic. Proc Natl Acad Sci USA.

[B9] Kirnbauer R, Taub J, Greenstone H, Roden R, Durst M, Gissmann L, Lowy DR, Schiller JT (1993). Efficient self-assembly of human papillomavirus type 16 L1 and L1-L2 into virus-like particles. J Virol.

[B10] Chen XS, Casini G, Harrison SC, Garcea RL (2001). Papillomavirus capsid protein expression in Escherichia coli: purification and assembly of HPV11 and HPV16 L1. J Mol Biol.

[B11] Bishop B, Dasgupta J, Klein M, Garcea RL, Christensen ND, Zhao R, Chen XS (2007). Crystal structures of four types of human papillomavirus L1 capsid proteins: understanding the specificity of neutralizing monoclonal antibodies. J Biol Chem.

[B12] Christensen ND, Kreider JW (1990). Antibody-mediated neutralization in vivo of infectious papillomaviruses. J Virol.

[B13] Rose RC, White WI, Li M, Suzich JA, Lane C, Garcea RL (1998). Human papillomavirus type 11 recombinant L1 capsomeres induce virus-neutralizing antibodies. J Virol.

[B14] White WI, Wilson SD, Palmer-Hill FJ, Woods RM, Ghim SJ, Hewitt LA, Goldman DM, Burke SJ, Jenson AB, Koenig S (1999). Characterization of a major neutralizing epitope on human papillomavirus type 16 L1. J Virol.

[B15] Christensen ND, Cladel NM, Reed CA, Budgeon LR, Embers ME, Skulsky DM, McClements WL, Ludmerer SW, Jansen KU (2001). Hybrid papillomavirus L1 molecules assemble into virus-like particles that reconstitute conformational epitopes and induce neutralizing antibodies to distinct HPV types. Virology.

[B16] Ludmerer SW, Benincasa D, Mark GE, Christensen ND (1997). A neutralizing epitope of human papillomavirus type 11 is principally described by a continuous set of residues which overlap a distinct linear, surface-exposed epitope. J Virol.

[B17] Li M, Cripe TP, Estes PA, Lyon MK, Rose RC, Garcea RL (1997). Expression of the human papillomavirus type 11 L1 capsid protein in Escherichia coli: characterization of protein domains involved in DNA binding and capsid assembly. J Virol.

[B18] Zhou J, Sun XY, Louis K, Frazer IH (1994). Interaction of human papillomavirus (HPV) type 16 capsid proteins with HPV DNA requires an intact L2 N-terminal sequence. J Virol.

[B19] Finnen RL, Erickson KD, Chen XS, Garcea RL (2003). Interactions between papillomavirus L1 and L2 capsid proteins. J Virol.

[B20] Kawana K, Yoshikawa H, Taketani Y, Yoshiike K, Kanda T (1999). Common neutralization epitope in minor capsid protein L2 of human papillomavirus types 16 and 6. J Virol.

[B21] Embers ME, Budgeon LR, Pickel M, Christensen ND (2002). Protective immunity to rabbit oral and cutaneous papillomaviruses by immunization with short peptides of L2, the minor capsid protein. J Virol.

[B22] Kawana Y, Kawana K, Yoshikawa H, Taketani Y, Yoshiike K, Kanda T (2001). Human papillomavirus type 16 minor capsid protein l2 N-terminal region containing a common neutralization epitope binds to the cell surface and enters the cytoplasm. J Virol.

[B23] Liu WJ, Gissmann L, Sun XY, Kanjanahaluethai A, Muller M, Doorbar J, Zhou J (1997). Sequence close to the N-terminus of L2 protein is displayed on the surface of bovine papillomavirus type 1 virions. Virology.

[B24] Büchen-Osmond C (2006). Index of Viruses – Papillomaviridae.

[B25] Edgar RC (2004). MUSCLE: a multiple sequence alignment method with reduced time and space complexity. BMC Bioinformatics.

[B26] Edgar RC (2004). MUSCLE: multiple sequence alignment with high accuracy and high throughput. Nucleic Acids Res.

[B27] Larkin MA, Blackshields G, Brown NP, Chenna R, McGettigan PA, McWilliam H, Valentin F, Wallace IM, Wilm A, Lopez R (2007). Clustal W and Clustal X version 2.0. Bioinformatics.

[B28] Crooks GE, Hon G, Chandonia JM, Brenner SE (2004). WebLogo: a sequence logo generator. Genome Res.

[B29] Berman HM, Westbrook J, Feng Z, Gilliland G, Bhat TN, Weissig H, Shindyalov IN, Bourne PE (2000). The Protein Data Bank. Nucleic Acids Res.

[B30] Bates PA, Kelley LA, MacCallum RM, Sternberg MJ (2001). Enhancement of protein modeling by human intervention in applying the automatic programs 3D-JIGSAW and 3D-PSSM. Proteins.

[B31] Karplus K, Barrett C, Hughey R (1998). Hidden Markov models for detecting remote protein homologies. Bioinformatics.

[B32] Karplus K, Karchin R, Barrett C, Tu S, Cline M, Diekhans M, Grate L, Casper J, Hughey R (2001). What is the value added by human intervention in protein structure prediction?. Proteins.

[B33] Karplus K, Karchin R, Draper J, Casper J, Mandel-Gutfreund Y, Diekhans M, Hughey R (2003). Combining local-structure, fold-recognition, and new fold methods for protein structure prediction. Proteins.

[B34] Karplus K, Katzman S, Shackleford G, Koeva M, Draper J, Barnes B, Soriano M, Hughey R (2005). SAM-T04: what is new in protein-structure prediction for CASP6. Proteins.

[B35] Bower MJ, Cohen FE, Dunbrack RL (1997). Prediction of protein side-chain rotamers from a backbone-dependent rotamer library: a new homology modeling tool. J Mol Biol.

[B36] Combita AL, Touze A, Bousarghin L, Christensen ND, Coursaget P (2002). Identification of two cross-neutralizing linear epitopes within the L1 major capsid protein of human papillomaviruses. J Virol.

[B37] Comeau SR, Gatchell DW, Vajda S, Camacho CJ (2004). ClusPro: a fully automated algorithm for protein-protein docking. Nucleic Acids Res.

[B38] Comeau SR, Gatchell DW, Vajda S, Camacho CJ (2004). ClusPro: an automated docking and discrimination method for the prediction of protein complexes. Bioinformatics.

[B39] Comeau SR, Kozakov D, Brenke R, Shen Y, Beglov D, Vajda S (2007). ClusPro: performance in CAPRI rounds 6–11 and the new server. Proteins.

[B40] Comeau SR, Vajda S, Camacho CJ (2005). Performance of the first protein docking server ClusPro in CAPRI rounds 3–5. Proteins.

[B41] Pastrana DV, Gambhira R, Buck CB, Pang YY, Thompson CD, Culp TD, Christensen ND, Lowy DR, Schiller JT, Roden RB (2005). Cross-neutralization of cutaneous and mucosal Papillomavirus types with anti-sera to the amino terminus of L2. Virology.

[B42] Gambhira R, Jagu S, Karanam B, Gravitt PE, Culp TD, Christensen ND, Roden RB (2007). Protection of rabbits against challenge with rabbit papillomaviruses by immunization with the N terminus of human papillomavirus type 16 minor capsid antigen L2. J Virol.

[B43] Gambhira R, Karanam B, Jagu S, Roberts JN, Buck CB, Bossis I, Alphs H, Culp T, Christensen ND, Roden RB (2007). A protective and broadly cross-neutralizing epitope of human papillomavirus L2. J Virol.

[B44] Chen XS, Stehle T, Harrison SC (1998). Interaction of polyomavirus internal protein VP2 with the major capsid protein VP1 and implications for participation of VP2 in viral entry. The EMBO journal.

[B45] Christensen ND, Dillner J, Eklund C, Carter JJ, Wipf GC, Reed CA, Cladel NM, Galloway DA (1996). Surface conformational and linear epitopes on HPV-16 and HPV-18 L1 virus-like particles as defined by monoclonal antibodies. Virology.

[B46] Ludmerer SW, Benincasa D, Mark GE (1996). Two amino acid residues confer type specificity to a neutralizing, conformationally dependent epitope on human papillomavirus type 11. J Virol.

[B47] Ludmerer SW, McClements WL, Wang XM, Ling JC, Jansen KU, Christensen ND (2000). HPV11 mutant virus-like particles elicit immune responses that neutralize virus and delineate a novel neutralizing domain. Virology.

[B48] McClements WL, Wang XM, Ling JC, Skulsky DM, Christensen ND, Jansen KU, Ludmerer SW (2001). A novel human papillomavirus type 6 neutralizing domain comprising two discrete regions of the major capsid protein L1. Virology.

[B49] Wang Z, Christensen N, Schiller JT, Dillner J (1997). A monoclonal antibody against intact human papillomavirus type 16 capsids blocks the serological reactivity of most human sera. J Gen Virol.

[B50] Garcia AM, Ortiz-Navarrete VF, Mora-Garcia ML, Flores-Borja F, Diaz-Quinonez A, Isibasi-Araujo A, Trejo-Becerril C, Chacon-Salinas R, Hernandez-Montes J, Granados-Arreola J (1999). Identification of peptides presented by HLA class I molecules on cervical cancer cells with HPV-18 infection. Immunol Lett.

[B51] Monroy-Garcia A, Weiss-Steider B, Hernandez-Montes J, Ortiz-Navarrete VF, Banuelos-Panuco A, Acosta-Araujo A, Diaz-Quinonez A, Lopez-Graniel CM, Herbert G, Granados J (2002). Identification of two homologous antigenic peptides derived from L1 HPV-16 and 18 proteins specific for the HLA-B*3901 allele. Arch Virol.

[B52] Shepherd PS, Rowe AJ, Cridland JC, Coletart T, Wilson P, Luxton JC (1996). Proliferative T cell responses to human papillomavirus type 16 L1 peptides in patients with cervical dysplasia. J Gen Virol.

[B53] Knappe M, Bodevin S, Selinka HC, Spillmann D, Streeck RE, Chen XS, Lindahl U, Sapp M (2007). Surface-exposed amino acid residues of HPV16 L1 protein mediating interaction with cell surface heparan sulfate. J Biol Chem.

[B54] Sapp M, Fligge C, Petzak I, Harris JR, Streeck RE (1998). Papillomavirus assembly requires trimerization of the major capsid protein by disulfides between two highly conserved cysteines. J Virol.

[B55] Touze A, Mahe D, El Mehdaoui S, Dupuy C, Combita-Rojas AL, Bousarghin L, Sizaret PY, Coursaget P (2000). The nine C-terminal amino acids of the major capsid protein of the human papillomavirus type 16 are essential for DNA binding and gene transfer capacity. FEMS Microbiol Lett.

[B56] Okun MM, Day PM, Greenstone HL, Booy FP, Lowy DR, Schiller JT, Roden RB (2001). L1 interaction domains of papillomavirus l2 necessary for viral genome encapsidation. J Virol.

[B57] Barouch DH, Harrison SC (1994). Interactions among the major and minor coat proteins of polyomavirus. J Virol.

[B58] Giroglou T, Sapp M, Lane C, Fligge C, Christensen ND, Streeck RE, Rose RC (2001). Immunological analyses of human papillomavirus capsids. Vaccine.

[B59] Breitburd F, Kirnbauer R, Hubbert NL, Nonnenmacher B, Trin-Dinh-Desmarquet C, Orth G, Schiller JT, Lowy DR (1995). Immunization with viruslike particles from cottontail rabbit papillomavirus (CRPV) can protect against experimental CRPV infection. J Virol.

[B60] Suzich JA, Ghim SJ, Palmer-Hill FJ, White WI, Tamura JK, Bell JA, Newsome JA, Jenson AB, Schlegel R (1995). Systemic immunization with papillomavirus L1 protein completely prevents the development of viral mucosal papillomas. Proc Natl Acad Sci USA.

[B61] Harper DM, Franco EL, Wheeler CM, Moscicki AB, Romanowski B, Roteli-Martins CM, Jenkins D, Schuind A, Costa Clemens SA, Dubin G (2006). Sustained efficacy up to 4.5 years of a bivalent L1 virus-like particle vaccine against human papillomavirus types 16 and 18: follow-up from a randomised control trial. Lancet.

[B62] Villa LL, Ault KA, Giuliano AR, Costa RL, Petta CA, Andrade RP, Brown DR, Ferenczy A, Harper DM, Koutsky LA (2006). Immunologic responses following administration of a vaccine targeting human papillomavirus Types 6, 11, 16, and 18. Vaccine.

[B63] Kondo K, Ishii Y, Ochi H, Matsumoto T, Yoshikawa H, Kanda T (2007). Neutralization of HPV16, 18, 31, and 58 pseudovirions with antisera induced by immunizing rabbits with synthetic peptides representing segments of the HPV16 minor capsid protein L2 surface region. Virology.

[B64] Kay BK, Williamson MP, Sudol M (2000). The importance of being proline: the interaction of proline-rich motifs in signaling proteins with their cognate domains. Faseb J.

[B65] Day PM, Roden RB, Lowy DR, Schiller JT (1998). The papillomavirus minor capsid protein, L2, induces localization of the major capsid protein, L1, and the viral transcription/replication protein, E2, to PML oncogenic domains. J Virol.

[B66] Florin L, Sapp C, Streeck RE, Sapp M (2002). Assembly and translocation of papillomavirus capsid proteins. J Virol.

