# Supplementary material for: Evolutionary and structural analyses of alpha-papillomavirus capsid proteins yields novel insights into L2 structure and interaction with L1
Source: Virol J. 2008 Dec 17;5:150. doi: 10.1186/1743-422X-5-150 (PMC2630942; doi:10.1186/1743-422X-5-150)
Supplement: Additional file 1 — Multiple sequence alignment of L1 and L2 proteins. Seventy-six alpha-HPV L1 and L2 sequences were obtained from the NCBI protein database under the Universal Virus Database, as well as six known HPV-16 L1 and L2 variant sequences provided by the ICTV. The sequences were aligned using the MUltiple Sequence Comparison by Log-Expectation (MUSCLE). MUSCLE outputs were loaded into CLUSTALX user interface for graphical representation of residue conservation and analysis. Sequence conservation is by the height of residue logos (indicated in bits), as generated by WebLogo 3. All outputs for both sequences are shown. [file 1743-422X-5-150-S1.pdf]

CLUSTAL 2.0.5 MULTIPLE SEQUENCE ALIGNMENT

File: /Users/johnlowe/Desktop/L1protein.ps **Date: Fri May 2 11:55:30 2008**


```
                                                                                                                                                   :   *:**  .   ::   : *       *:..:
[HPV7]                     ---------------------------------------------------------------------------------------------------------------MWQLNENQVYLPPPTPVATIV-STDEYVQRTSLYYHAGS 38
[HPV-7]                    ---------------------------------------------------------------------------------------------------------------MWQLNENQVYLPPPTPVATIV-STDEYVQRTSLYYHAGS 38
[HPV40]                    ---------------------------------------------------------------------------------------------------------------MWQLNENQVYLPPPTPVATIV-STDEYVQRTSLYYHAGS 38
[HPV43]                    -----------------------------------------------------------------------------------MALIFICILHCGIWANAVNAFHIFLQITMWRLNDNKVYLPPPGPIASIV-STDEYVQRTNLFYYAGS 66
[HPV91]                    ---------------------------------------------------------------------------------------------------------------MWRLNDNKVYLPPPGPIASIV-STDEYVQRTNLFYHAGS 38
[HPV6b]                    ---------------------------------------------------------------------------------------------------------------MWRPSDSTVYVPPPNPVSKVV-ATDAYVTRTNIFYHASS 38
[HPV6bNP_040304.1]         ---------------------------------------------------------------------------------------------------------------MWRPSDSTVYVPPPNPVSKVV-ATDAYVTRTNIFYHASS 38
[HPV11]                    ---------------------------------------------------------------------------------------------------------------MWRPSDSTVYVPPPNPVSKVV-ATDAYVKRTNIFYHASS 38
[HPV13]                    ---------------------------------------------------------------------------------------------------------------MWRPSDNKLYVPPPAPVSKVI-TTDAYVTRTNIFYHASS 38
[PCP                       ---------------------------------------------------------------------------------------------------------------MWRPSDNKLYVPPPAPVSKVI-TTDAYVTRTKIFYHASS 38
[CCP                       ---------------------------------------------------------------------------------------------------------------MWRPSDNKLYVPPPAPVSKVL-TTDAYVTRTKIFYHASS 38
[HPV74]                    -----------------------------------------------------------------------------------MVLHFIYIHHGILHANAVNVFPCF--LQMWRPSDNQVYVPPPAPVSKVI-STDAYVTRTNIFYHASS 64
[HPV44]                    ---------------------------------------------------------------------------------------------------------------MWRPSENQVYVPPPAPVSKVI-PTDAYVKRTNIYYHASS 38
[hpv55]                    ---------------------------------------------------------------------------------------------------------------MWRPSENQVYVPPPAPVSKVI-TTDAYVKRTNIVYHASS 38
HPV34_L1aa                 -----------------------------------------------------------------------------------MGPILYCILACMSFHANVNVCHIF--LQMWRPTEAKVYLPPV-SVSKVV-STEEYVTRTNIYYYAGS 63
[HPV34NP_041812.1]         -----------------------------------------------------------------------------------MGPILYCILACMSFHANVNVCHIF--LQMWRPTEAKVYLPPV-SVSKVV-STEEYVTRTNIYYYAGS 63
[HPV34CAA52560.1]          -----------------------------------------------------------------------------------MGPILYCILACMSFHANVNVCHIF--LQMWRPTEAKVYLPPV-SVSKVV-STEEYVTRTNIYYYAGS 63
HPV73CAA63887.1            ---------------------------------------------------------------------------------------------------------------MWRPTDAKVYLPPV-SVSKVV-STDEYVTRTNIYYYAGS 37
[HPV54]                    ---------------------------------------------------------------------------------------------------------------MWRPSENKVYLPPT-PVSKVV-STDEYVTRTSIYYHASS 37
[HPV32]                    -------------------------------------------------------------------------------------------------------------MSVWRPSDNKVYLPPP-PVSKVV-STDEYVQRTNYFYHASS 39
[HPV32CAA52554.1]          -------------------------------------------------------------------------------------------------------------MSVWRPSDNKVYLPPP-PVSKVV-STDEYVQRTNYFYHASS 39
HPV42                      -------------------------------------------------------------------------------------------------------------MSVWRPSDNKVYLPPP-PVSKVV-STDEYVQRTNYFYHASS 39
RhesusPV1                  -------------------------------------------------------------------------------------------------------------MSMWRPSDSKVYLPPV-PVSKVV-STDEYVSRTSIYYHAGS 39
HPV52_L1aa                 -----------------------------------------------------------------------------------MVQILFYILVIFYYVAGVNVFHIFLQMSVWRPSEATVYLPPV-PVSKVV-STDEYVSRTSIYYYAGS 65
HPV67_L1aa                 --------------------------------------------------------------------------------MYLFMVVILFYTLVIFYVAGVNGFHIFLQMSVWRPSEATVYLPPV-PVSKVV-STDEYVSRTSIYYYAGS 68
HPV33_L1aa                 -------------------------------------------------------------------------------------------------------------MSVWRPSEATVYLPPV-PVSKVV-STDEYVSRTSIYYYAGS 39
HPV58_L1aa                 -----------------------------------------------------------------------------------MVLILCCTLAILFCVADVNVFHIFLQMSVWRPSEATVYLPPV-PVSKVV-STDEYVSRTSIYYYAGS 65
HPV35                      -------------------------------------------------------------------------------------------------------------MSLWRSNEATVYLPPV-SVSKVV-STDEYVTRTNIYYHAGS 39
HPV31_L1aa                 -------------------------------------------------------------------------------------------------------------MSLWRPSEATVYLPPV-PVSKVV-STDEYVTRTNIYYHAGS 39
HPV16NP_041332.1           -----------------------------------------------------------------------------------MQVTFIYILVITCYENDVNVYHIFFQMSLWLPSEATVYLPPV-PVSKVV-STDEYVARTNIYYHAGT 65
HPV16_L1aa                 -----------------------------------------------------------------------------------MQVTFIYILVITCYENDVNVYHIFFQMSLWLPSEATVYLPPV-PVSKVV-STDEYVARTNIYYHAGT 65
[HPV16eaAF534061]          -----------------------------------------------------------------------------------MQVTFIYILVITCYENDVNVYHIFFQMSLWLPSEATVYLPPV-PVSKVV-STDEYVARTNIYYHAGT 65
[HPV16gerAAQ10719.1]       -----------------------------------------------------------------------------------MQVTFIYILVITCYENDVNVYHIFFQMSLWLPSEATVYLPPV-PVSKVV-STDEYVARTNIYYHAGT 65
[HPV16af2]                 -----------------------------------------------------------------------------------MEVTFIYILVITCYENDVNVYHIFFQMSLWLPSEATVYLPPV-PVSKVV-STDEYVARTNIYYHAGT 65
[HPV16asamAF402678]        -----------------------------------------------------------------------------------MEVTFIYILVITCYENDVNVYHIFFQMSLWLPSEATVYLPPV-PVSKVV-STDEYVARTNIYYHAGT 65
[HPV16aa]                  -----------------------------------------------------------------------------------MEVTFIYILVITCYENDVNVYHIFFQMSLWLPSEATVYLPPV-PVSKVV-STDEYVARTNIYYHAGT 65
[HPV16af1]                 -----------------------------------------------------------------------------------MEVTFIYILVITCYENDVNVYHIFFQMSLWLPSEATVYLPPV-PVSKVV-STDEYVARTNIYYHAGT 65
HPV51                      -------------------------------------------------------------------------------------------------------------MALWRTNDSKVYLPPA-PVSRIV-NTEEYITRTGIYYYAGS 39
[HPV82]                    -------------------------------------------------------------------------------------------------------------MALWRTNDSKVYLPPA-PVSRIV-NTEEYITRTGIYYYAGS 39
HPV26NP_041787.1           -------------------------------------------------------------------------------------------------------------MALWRTSDSKVYLPPT-PVSRVV-NTDEYVTRTGIYYYAGS 39
HPV26bCAA52535.1           -------------------------------------------------------------------------------------------------------------MALWRTSDSKVYLPPT-PVSRVV-NTDEYVTRTGIYYYAGS 39
[HPV69]                    -------------------------------------------------------------------------------------------------------------MALRRTSDSKVYLPPT-PVSRVVRRMNMYTHR--IYYYAGS 38
HPV18_X05015               ------------------------------------------------MCLYTRVLILHYHLLPLYGPLYHPRPLPLHSILVYMVHIIICGHYIILFLRNVNVFPIFLQMALWRPSDNTVYLPPP-SVARVV-NTDDYVTPTSIFYHAGS 100
HPv18_NC001357             ------------------------------------------------MCLYTRVLILHYHLLPLYGPLYHPRPLPLHSILVYMVHIIICGHYIILFLRNVNVFPIFLQMALWRPSDNTVYLPPP-SVARVV-NTDDYVTPTSIFYHAGS 100
HPV45_L1aa                 -----------------------------------------------------------------------------------MAHNIIYGHGIIIFLKNVNVFPIFLQMALWRPSDSTVYLPPP-SVARVV-STDDYVSRTSIFYHAGS 65
HPV59_L1aa                 -------------------------------------------------------------------------------------------------------------MALWRSSDNKVYLPPP-SVAKVV-STDEYVTRTSIFYHAGS 39
HPVcand85_L1aa             -------------------------------------------------------------------------------------------------------------MALWRSSDSKVYLPPP-SVAKVV-NTDDYVTRTSTFYHAGS 39
HPV68_L1aa                 -------------------------------------------------------------------------------------------------------------MALWRASDNMVYLPPP-SVAKVV-NTDDYVTRTGMYYYAGT 39
HPV39_L1aa                 -------------------------------------------------------------------------------------------------------------MAMWRSSDSMVYLPPP-SVAKVV-NTDDYVTRTGIYYYAGS 39
HPV70_L1aa                 -------------------------------------------------------------------------------------------------------------MALWRSSDNTVYLPPP-SVAKVV-NTDDYVTRTGIYYYAGS 39
HPV29                      -------------------------------------------------------------------------------------------------------------MALWRSSDNLVYLPPT-PVSKVI-STDDYVTRTNIYYYAGS 39
[HPV77]                    -------------------------------------------------------------------------------------------------------------MALWRSSDNLVYLPPT-PVSKVI-STDDYVTRTNVYYYAGS 39
[HPV94]                    ---------------------------------------------------------------------------------MEGIFISGLLPFTCPDVVVVNVFNIVLQMALWRPSDNLVYLPPT-PVSKVL-STDDYVTRTNIYYYAGT 67
[HPV10NP041746.1]          ---------------------------------------------------------------------------------MAGISIFGLLPFTSPDIVAVNVSHIFLQMALWRSSDNLVYLPPT-PVSKVL-STDDYVTRTNIYYYAGT 67
[HPV10CAA52494.1]          ---------------------------------------------------------------------------------MAGISIFGLLPFTSPDIVAVNVSHIFLQMALWRSSDNLVYLPPT-PVSKVL-STDDYVTRTNIYYYAGT 67
[HPV-10CAA52495.1]         -------------------------------------------------------------------------------------------------------------MALWRSSDNLVYLPPT-PVSKVL-STDDYVTRTNIYYYAGT 39
[HPV3]                     ---------------------------------------------------------------------------------MAGIFIYGLSPSFCPDVVAVNVSHIFLQMALWRSSDNLVYLPPT-PVSKVL-STDDYVTRTNIYYYAGS 67
HPV28                      -------------------------------------------------------------------------------------------------------------MALWRSSDNLVYLPPT-PVSKVL-STDDYVTRTNIYYYAGT 39
HPV56CAA52600.1            --------------------------------------------------------------------------MMLPMMYIYRDPPLHYGLCIFLDVGAVNVFPIFLQMATWRPSENKVYLPPT-PVSKVV-ATDSYVKRTSIFYHAGS 74
HPV66                      -------------------------------------------------------------------------------------------------------------MAMWRPSDNKVYLPPT-PVSKVV-ATDTYVKRTSIFYHAGS 39
[HPV53]                    -------------------------------------------------------------------------------------------------------------MAVWRPSDSKVYLPPT-PVSKVI-TTDAYVKRTTIFYHAGS 39
[HPV53CAA52595.1]          -------------------------------------------------------------------------------------------------------------MAVWRPSDSKVYLPPT-PVSKVI-TTDAYVKRTTIFYHAGS 39
[HPV30]                    ------------------------------------------------------------------------------------------------------MFPIFLQMAVWRPSETKVYLPPT-PVSKVV-PTDAYVKRTNIFYHAGS 46
[HPV71]                    ------------------------------------------------------------------------------------------MPSYIFFPKKLNVSTIFLQMALWRPSDSKVYLPPA-PRIQVL-STDDYVTRTKLFYYAGS 58
[HPV81]                    ---------------------------------------------------------------------------------MLLALIFTCFLLIYSSLKNINVFTILLQMAMWRPGDGKVYLPPT-PVSKVI-STDAYVTRTNLFYYGGS 67
[HPV61]                    -------------------------------------------------------------------------------------------------------------MALWRPGDGKVYLPPT-PVSKVI-STDRYVQRTNLFYYGGS 39
[HPV72]                    ---------------------------------------------------------------------------------MLRALIFICCLHISFFLNVVNVCPILLQMALWRPGDGKVYLPPN-PVSKVL-STDRYVQRTNLYYYGGS 67
[HPV84]                    -------------------------------------------------------------------------------------------------------------MALWRPGDGKVYLPPT-PVSKVI-STDRYVSRTNLFYYGGS 39
[HPV86]                    -------------------------------------------------------------------------------------------------------------MALWRPGDGKVYLPPT-PVSKVL-STDRYVSRTNLFYYGGS 39
[HPVcand87]                -------------------------------------------------------------------------------------------------------------MALWRPGDGKVYLPPT-PVSKVI-STDRYVSRTNLFYYGGS 39
HPV83AAD38974.1|AF151983_7 -------------------------------------------------------------------------------------------------------------MAMWRPGDGKTYLPPT-PVSKVI-STDRYVTRTNLFYYGGS 39
[HPVcand89]                ---------------------------------------------------------------------------------------------MCFFLKGVNVSPILLQMALWRPGDGKVYLPPT-PVSKVL-STDRYVQRTNLYYYGGS 55
[HPV90]                    -------------------------------------------------------------------------------------------------------------MAMWKPSDNKVYLPPA-PVSKVL-STDAYVSRTNIYYYAGS 39
[HPV57]                    ---------------------------------------------------------------------------------------------MFCGLNDVNVCTISLQMAMWRPNESKVYLPPT-PVSKVL-STDVYVTRTNVYYHGGS 55
[HPV-2]                    ---------------------------------------------------------------------------------------------MSCGLNDVNVSTISLQMALWRPNESKVYLPPT-PVSKVI-STDVYVTRTNVYYHGGS 55
[HPV27]                    MMFMLILMSCSHWTITTQPLAAPWLIPRYLPPLHLHCEGPLQPLSRVVLMCLCILGLILNHPLFLVWGPSFLWLHRCHPLFTYLGGIIIYCQVISCGLNDVNVSTISLQMALWRPNESKVYLPPT-PVSKVI-STDVYVTRTNVYYHGGS 148
                           1.......10........20........30........40........50........60........70........80........90.......100.......110.......120.......130.......140.......150
```

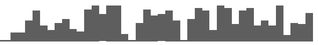

CLUSTAL 2.0.5 MULTIPLE SEQUENCE ALIGNMENT

File: /Users/johnlowe/Desktop/L1protein.ps **Date: Fri May 2 11:55:30 2008**

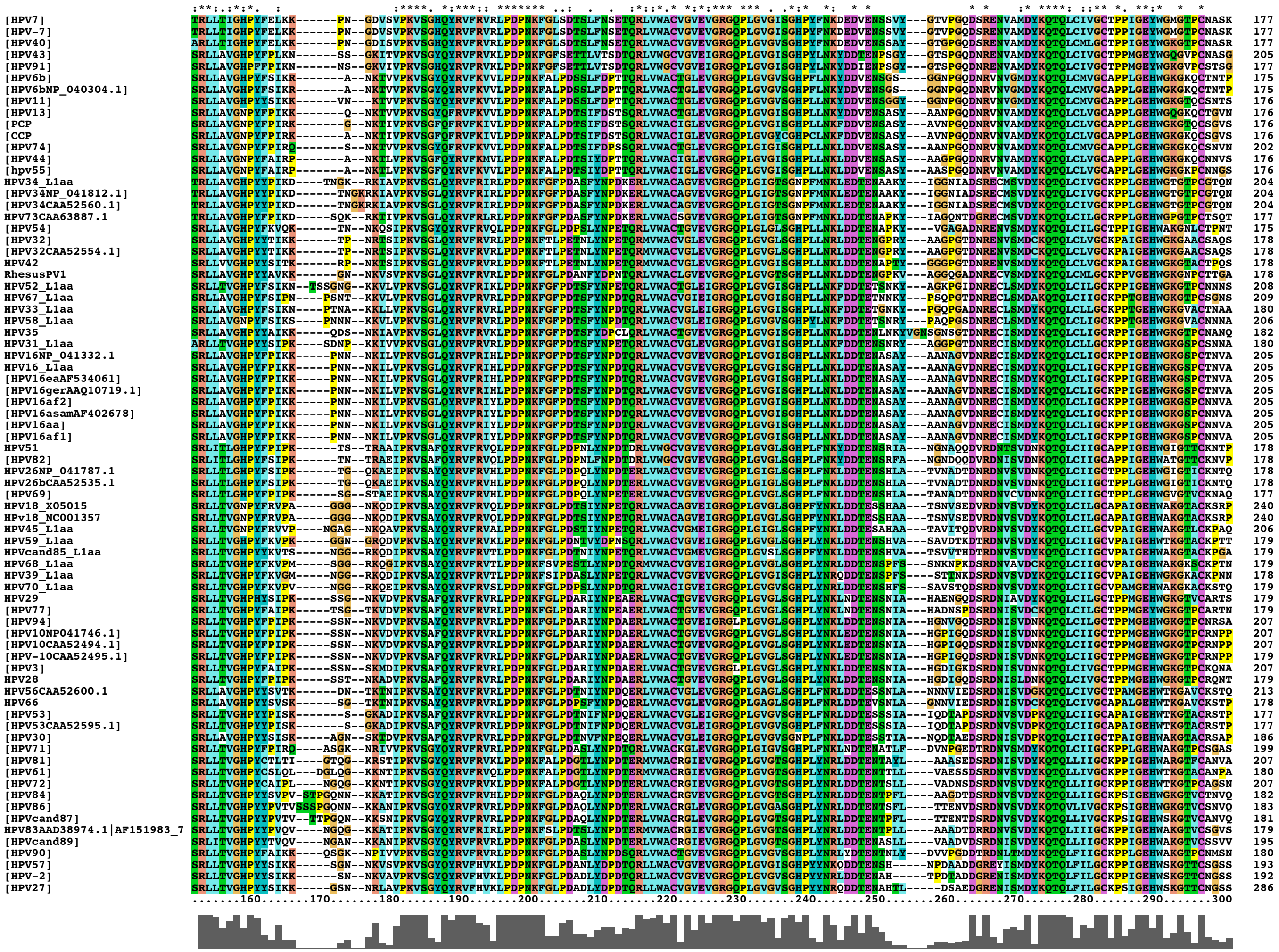

CLUSTAL 2.0.5 MULTIPLE SEQUENCE ALIGNMENT


```
                                   ** : : .  ::****. *:*.::* **  :.:**:*:    ** : :   .  *: ::* :*:** * :  *     *  :.                         *
[HPV7]                        VSP--GDCPVLELKSEVIEDGDMVDAGFGAMDFASLQANKSDVPLDLCTSISKYPDYLGMAAEPYGNSLFFFLRREQMFVRHFFNRAGTTGDSVPNDLYITG---SSNRASIAGSIYYSTPSGSLVTSDSQIFNK--PLWIQKAQGHNNG   320
[HPV-7]                       VSP--GDCPVLELKSEVIEDGDMVDAGFGAMDFASLQANKSDVPLDLCTSISKYPDYLGMAAEPYGNSLFFFLRREQMFVRHFFNRAGTTGDSVPNDLYITG---SSNRASIAGSIYYSTPSGSLVTSDSQIFNK--PLWIQKAQGHNNG   320
[HPV40]                       VTL--GDCPVLELKTEVIQDGDMVDTGFGAMDFASLQANKSDVPLDLCTSISKYPDYLGMAAEPYGNSLFFFLRREQMFVRHFFNRAGTTGDSVPTDLYITG---TSGRTPIAGSIYYSTPSGSLVTSDSQIFNK--PLWIQKAQGHNNG   320
[HPV43]                       VTQ--GDCPVIELKSEVIQDGDMVDTGFGAMDFASLQASKSDVPLDLVNTKSKYPDYLGMAAEPYGNSLFFFLRREQMFLRHFFNKAGKTGDVVPSDMYIAG---SNTRSKIADSIYFSTPSGSLVTSDSQLFNK--PLWIQKAQGHNNG   348
[HPV91]                       ITQ--GDCPVIELKSEVIEDGDMVDTGFGALDFASLQASKSDVPLDLCNTKSKYPDYLGMAAEPYGNSLFFFLRREQMFVRHFFNRAGTTGDAVPKDLYIAG---TGNRANIAGSIYYSTPSG-------------------------   295
[HPV6b]                       VQA--GDCPPLELITSVIQDGDMVDTGFGAMNFADLQTNKSDVPIDICGTTCKYPDYLQMAADPYGDRLFFFLRKEQMFARHFFNRAGEVGEPVPDTLIIKG---SGNRTSVGSSIYVNTPSGSLVSSEAQLFNK--PYWLQKAQGHNNG   318
[HPV6bNP_040304.1]            VQA--GDCPPLELITSVIQDGDMVDTGFGAMNFADLQTNKSDVPIDICGTTCKYPDYLQMAADPYGDRLFFFLRKEQMFARHFFNRAGEVGEPVPDTLIIKG---SGNRTSVGSSIYVNTPSGSLVSSEAQLFNK--PYWLQKAQGHNNG   318
[HPV11]                       VQN--GDCPPLELITSVIQDGDMVDTGFGAMNFADLQTNKSDVPLDICGTVCKYPDYLQMAADPYGDRLFFYLRKEQMFARHFFNRAGTVGEPVPDDLLVKG---GNNRSSVASSIYVHTPSGSLVSSEAQLFNK--PYWLQKAQGHNNG   319
[HPV13]                       VQP--GDCPPLELISSVIQDGDMVDTGFGAMNFAELQSNKSDVPLDICTSTCKYPDYLQMAADPYGDRLFFYLRKEQMFARHFFNRAGSVGEQIPAELYVKG---SN---TLSNSIYYNTPSGSLVSSEAQLFNK--PYWLQKAQGHNNG   316
[PCP                          VQD--GDCPPLELVTSVIQDGDMVDTGFGAMDFAQLQSNKSDVPLDICTATCKYPDYLQMAADPYGDRLFFSLRKEQMFARHFFNRAGTVGEQIPEDLLVKG---TTSRATVSSTIYFNTPSGSLVSSEAQLFNK--PYWLHKAQGHNNG   319
[CCP                          VQD--GDCPPLELVTSVIQDGDMVDTGFGAMDFAELQSNKSDVPLDICTSTCKYPDYLQMAADPYGDRLFFYLRKEQMFARHFFNRAGTVGEQIPDELFVKG---TTSRATVSSNIYFNTPSGSLVSSEAQLFNK--PYWLHKAQGHNNG   319
[HPV74]                       VKP--GDCPALELITSVIQDGDMVDTGFGAMNFADLQPIKSDVPLDICNTTCKYPDYLQMAADPYGDRLFFYLRKEQMFARHFFNRAGTVGEDIPQDLYIKG----TTRSTLANAIYFNTPSGSLVSSETQLFNK--PFWLQRAQGHNNG   344
[HPV44]                       VKD--GDCPPLELITSVIEDGDMVDTGFGAMNFAELQPNKSDVPLDICTATCKYPDYLQMAADPYGDRLFFYLRKEQMFARHFFNRAGTVGEDVSQDLVIKS----ASKNTVPNAIYFNTPSGSLVSSETQLFNK--PFWLQKAQGHNNG   318
[hpv55]                       VNS--GDCPPLELITSVIEDGDMVDTGFGAMNFAELQPNKSDVPLDICTATCKYPDYLQMAADPYGDRLFFYLRKEQMFARHFFNRAGTVGEDIPQDLVFKG----ATKSTVPNAIYFNTPSGSLVSSETQLFNK--PFWLQRAQGHNNG   318
HPV34_L1aa                    A----GECPPLELKNTTIQDGDMIDVGFGAMDFKALQANKSDVPIDISNTICKYPDYLGMAADPYGDSMWFYIRREQMFVRHLFNRAGTVGDAIPDDLMIKG---TGNTASPSSCVFYPTPSGSMVSSDAQIFNK--PYWLQKAQGQNNG   345
[HPV34NP_041812.1]            A----GECPPLELKNTTIQDGDMIDVGFGAMDFKALQANKSDVPIDISNTICKYPDYLGMAADPYGDSMWFYIRREQMFVRHLFNRAGTVGDAIPDDLMIKG---TGNTASPSSCVFYPTPSGSMVSSDAQIFNK--PYWLQKAQGQNNG   345
[HPV34CAA52560.1]             A----GECPPLELKNTTIQDGDMIDVGFGAMDFKALQANKSDVPIDISNTICKYPDYLGMAADPYGDSMWFYIRREQMFVRHLFNRAGTVGDAIPDDLMIKG---TGNTASPSSCVFYPTPSGSMVSSDAQIFNK--PYWLQKAQGQNNG   345
HPV73CAA63887.1               VNT--GDCPPLELKNTPIQDGDMIDVGFGAMDFKALQANKSDVPIDISNTTCKYPDYLGMAADPYGDSMWFYLRREQMFVRHLFNRAGDTGDKIPDDLMIKG---TGNTATPSSCVFYPTPSGSMVSSDAQLFNK--PYWLQKAQGQNNG   320
[HPV54]                       LAA--GDCPPLELVNSYIQDGDMVDIGFGAMDFKTLQTSKSEVPLDVATSICKYPDYLKMAAEAYGDSLFFYLRREQMFVRHMLNRAGTMGEPVPNDLYIKK---SSG--NLDSSIYAATPSGSMVTSEYQIFNK--PYWLQRAQGQNNG   316
[HPV32]                       N----GDCPPLELQNSVIQDGDMADVGFGAMDFSALQTSKAEVPLDIMNSISKYPDYLKMSAEAYGDNMFFFLRREQMFVRHLFNRAGTLGEPVPEDMYIKASNGASGRNNLASSIYYPTPSGSMVTSDAQIFNK--PYWLQQAQGHNNG   322
[HPV32CAA52554.1]             N----GDCPPLELQNSVIQDGDMADVGFGAMDFSALQTSKAEVPLDIMNSISKYPDYLKMSAEAYGDNMFFFLRREQMFVRHLFNRAGTLGEPVPEDMYIKASNGASGRNNLASSIYYPTPSGSMVTSDAQIFNK--PYWLQQAQGHNNG   322
HPV42                         N----GDCPPLELKNSFIQDGDMVDVGFGALDFGALQSSKAEVPLDIVNSITKYPDYLKMSAEAYGDSMFFFLRREQMFVRHLFNRAGAIGEPVPDELYTKAANNASGRHNLGSSIYYPTPSGSMVTSDAQLFNK--PYWLQQAQGHNNG   322
RhesusPV1                     A----GDCPALELVNSVIQDGDMVDTGYGAMDFNALQANKSDVPIDICTSVCKYPDYLKMASDPYGDSLFFYLRREQMFVRHLFNRAGTMGDSVPDDLYIKG---SGSNVKLASHVFYPTPSGSMVTSDAQLFNK--PYWLQKAQGHNNG   319
HPV52_L1aa                    GNP--GDCPPLQLINSVIQDGDMVDTGFGCMDFNTLQASKSDVPIDICSSVCKYPDYLQMASEPYGDSLFFYLRREQMFVRHFFNRAGTLGDPVPGDLYIQG-SNSGNTATVQSSAFFPTPSGSMVTSESQLFNK--PYWLQRAQGHNNG   353
HPV67_L1aa                    NGP--GACPPLELMNTVIEDGDMIDTGFGCMDFKSLQANKSDVPLDICTSICKYPDYLGMASEAYGDSLFFFLRREQMFVRHLFNRAGKLGEDVPTDLYFKG---SANTSALQTSAFFPTPSGSMVSSESQLFNK--PYWLQRAQGHNNG   352
HPV33_L1aa                    PA---NDCPPLELINTIIEDGDMVDTGFGCMDFKTLQANKSDVPIDICGSTCKYPDYLKMTSEPYGDSLFFFLRREQMFVRHFFNRAGTLGEAVPDDLYIKG---SGTTASIQSSAFFPTPSGSMVTSESQLFNK--PYWLQRAQGHNNG   322
HPV58_L1aa                    AA---TDCPPLELFNSIIEDGDMVDTGFGCMDFGTLQANKSDVPIDICNSTCKYPDYLKMASEPYGDSLFFFLRREQMFVRHFFNRAGKLGEAVPDDLYIKG---SGNTAVIQSSAFFPTPSGSIVTSESQLFNK--PYWLQRAQGHNNG   348
HPV35                         VKA--GECPPLELLNTVLQDGDMVDTGFGAMDFTTLQANKSDVPLDICSSICKYPDYLKMVSEPYGDMLFFYLRREQMFVRHLFNRAGTVGETVPADLYIKG---TTG--TLPSTSYFPTPSGSMVTSDAQIFNK--PYWLQRAQGHNNG   323
HPV31_L1aa                    ITP--GDCPPLELKNSVIQDGDMVDTGFGAMDFTALQDTKSNVPLDICNSICKYPDYLKMVAEPYGDTLFFYLRREQMFVRHFFNRSGTVGESVPTDLYIKG---SGSTATLANSTYFPTPSGSMVTSDAQIFNK--PYWMQRAQGHNNG   323
HPV16NP_041332.1              VNP--GDCPPLELINTVIQDGDMVHTGFGAMDFTTLQANKSEVPLDICTSICKYPDYIKMVSEPYGDSLFFYLRREQMFVRHLFNRAGTVGENVPDDLYIKG---SGSTANLASSNYFPTPSGSMVTSDAQIFNK--PYWLQRAQGHNNG   348
HPV16_L1aa                    VNP--GDCPPLELINTVIQDGDMVHTGFGAMDFTTLQANKSEVPLDICTSICKYPDYIKMVSEPYGDSLFFYLRREQMFVRHLFNRAGTVGENVPDDLYIKG---SGSTANLASSNYFPTPSGSMVTSDAQIFNK--PYWLQRAQGHNNG   348
[HPV16eaAF534061]             VTP--GDCPPLELINTVIQDGDMVDTGFGAMDFTTLQANKSEVPLDICTSICKYPDYIKMVSEPYGDSLFFYLRREQMFVRHLFNRAGAVGENVPDDLYIKG---SGSTANLASSNYFPTPSGSMVTSDAQIFNK--PYWLQRAQGHNNG   348
[HPV16gerAAQ10719.1]          VNP--GDCPPLELINTVIQDGDMVDTGFGAMDFTTLQANKSEVPLDICTSICKYPDYIKMVSEPYGDSLFFYLRREQMFVRHLFNRAGAVGENVPDDLYIKG---SGSTANLASSNYFPTPSGSMVTSDAQIFNK--PYWLQRAQGHNNG   348
[HPV16af2]                    VTP--GDCPPLELINTVIQDGDMVDTGFGAMDFTTLQANKSEVPLDICTSICKYPDYIKMVSEPYGDSLFFYLRREQMFVRHLFNRAGAVGENVPDDLYIKG---SGPTANLASSNYFPTPSGSMVTSDAQIFNK--PYWLQRAQGHNNG   348
[HPV16asamAF402678]           VTP--GDCPPLELINTVIQDGDMVDTGFGAMDFTTLQANKSEVPLDICTSICKYPDYIKMVSEPYGDSLFFYLRREQMFVRHLFNRAGAVGENVPDDLYIKG---SGSTANLASSNYFPTPSGSMVTSDAQIFNK--PYWLQRAQGHNNG   348
[HPV16aa]                     VNP--GDCPPLELINTVIQDGDMVDTGFGAMDFTTLQANKSEVPLDICTSICKYPDYIKMVSEPYGDSLFFYLRREQMFVRHLFNRAGAVGENVPDDLYIKG---SGSTANLASSNYFPTPSGSMVTSDAQIFNK--PYWLQRAQGHNNG   348
[HPV16af1]                    VNP--GDCPPLELINTVIQDGDMVDTGFGAMDFTTLQANKSEVPLDICTSICKYPDYIKMVSEPYGDSLFFYLRREQMFVRHLFNRAGAVGENVPDDLYIKG---SGSTANLASSNYFPTPSGSMVTSDAQIFNK--PYWLQRAQGHNNG   348
HPV51                         VPP--GDCPPLELVSSVIQDGDMIDTGFGAMDFAALQATKSDVPLDISQSVCKYPDYLKMSADTYGNSMFFHLRREQIFARHYYNKLVGVGEDIPNDYYIKG--SGNGRDPIESYIYSATPSGSMITSDSQIFNK--PYWLHRAQGHNNG   322
[HPV82]                       VPQ--GDCPPLELVSTVIEDGDMVDTGFGAMDFANLQATKSDVPLDIAQSVCKYPDYLKMSADTYGNSMFFHLRREQIFARHYYNKAGVVGDAIPDKAYIKG--TGAGRDPISSYIYSATPSGSMITSDSQIFNK--PYWLHRAQGHNNG   322
HPV26NP_041787.1              TQR--GDCPPLELISSIIEDGDMIDTGFGAMDFTALQATKSDVPIDISQSTCKYPDYLKMSADTYGNSMFFFLRREQLFARHFYNKAGAVGDAIPTTLYIKG--AESGREPPTSSIYSATPSGSMVTSDAQLFNK--PYWLQRAQGHNNG   322
HPV26bCAA52535.1              TQR--GDCPPLELISSIIEDGDMIDTGFGAMDFTALQATKSDVPIDISQSTCKYPDYLKMSADTYGNSMFFFLRREQLFARHFYNKAGAVGDAIPTTLYIKG--AESGREPPTSSIYSATPSGSMVTSDAQLFNK--PYWLQRAQGHNNG   322
[HPV69]                       SQVQRGDCPPLELISSVIEDGDMIDTGFGAMDFTALQATKCDVPLDINQSICKYPDYLKMSADTYGNSMFFFLRREQLFARHFFNKAGTIGDSVPVSMYIKG--AGQGREPPTTSIYSRTPSGSMVTSDAQLFNK--PYWLQRAQGHNNG   323
HPV18_X05015                  LSQ--GDCPPLELKNTVLEDGDMVDTGYGAMDFSTLQDTKCEVPLDICQSICKYPDYLQMSADPYGDSMFFCLRREQLFARHFWNRAGTMGDTVPQSLYIKG---TGMPASPGSCVYSPSPSGSIVTSDSQLFNK--PYWLHKAQGHNNG   383
HPv18_NC001357                LSQ--GDCPPLELKNTVLEDGDMVDTGYGAMDFSTLQDTKCEVPLDICQSICKYPDYLQMSADPYGDSMFFCLRREQLFARHFWNRAGTMGDTVPQSLYIKG---TGMPASPGSCVYSPSPSGSIVTSDSQLFNK--PYWLHKAQGHNNG   383
HPV45_L1aa                    LQP--GDCPPLELKNTIIEDGDMVDTGYGAMDFSTLQDTKCEVPLDICQSICKYPDYLQMSADPYGDSMFFCLRREQLFARHFWNRAGVMGDTVPTDLYIKG-TSANMRETPGSCVYSPSPSGSIITSDSQLFNK--PYWLHKAQGHNNG   351
HPV59_L1aa                    VVQ--GDCPPLELINTPIEDGDMVDTGYGAMDFKLLQDNKSEVPLDICQSICKYPDYLQMSADAYGDSMFFCLRREQVFARHFWNRSGTMGDQLPESLYIKG---TDIRANPGSYLYSPSPSGSVVTSDSQLFNK--PYWLHKAQGLNNG   322
HPVcand85_L1aa                VQT--GDCPPLELVNTPIEDGDMIDTGYGAMDFSTLQDNKSEVPLDICQSICKYPDYLQMSADAYGDSMFFCLRREQLFARHFWNRGGTIGDAVPETLYIKG---TNDRATPGSCIYSPSPSGSMVSSDAQMFNK--PYWLHKAQGQNNG   322
HPV68_L1aa                    VQQ--GDCPPLELVNTPIEDGDMIDTGYGAMDFGTLQETKSEVPLDICQSVCKYPDYLQMSADVYGDSMFFCLRREQLFARHFWNRGGMVGDTIPTDMYIKG---TDIRETPSSYVYAPSPSGSMVSSDSQLFNK--PYWLHKAQGHNNG   322
HPV39_L1aa                    VST--GDCPPLELVNTPIEDGDMIDTGYGAMDFGALQETKSEVPLDICQSICKYPDYLQMSADVYGDSMFFCLRREQLFARHFWNRGGMVGDAIPAQLYIKG---TDIRANPGSSVYCPSPSGSMVTSDSQLFNK--PYWLHKAQGHNNG   321
HPV70_L1aa                    Q----GDCPPLELVNTAIEDGDMIDTGYGAMDFRTLQETKSEVPLDICQSVCKYPDYLQMSADVYGDSMFFCLRKEQLFARHFWNRGGMVGDTIPSELYIKG---TDIRERPGTHVYSPSPSGSMVSSDSQLFNK--PYWLHKAQGHNNG   320
HPV29                         SAA--GDCPPLELMTTHIEDGDMVDTGYGAMDFAALQVNKSDVPLDICQSTCKYPDYLGMAADPYGDSMFFFLRREQLFARHFFNRAGVVGDKIPDSLYLKG---NNGRETPGSAIYSPTPSGSMVTSEAQIFNK--PYWLQQAQGHNNG   322
[HPV77]                       TTP--GDCPPLELMTSYIQDGDMVDTGYGAMDFTALQFNKSDVPLDICQSICKYPDYLGMAADPYGDSMFFFLRREQLFARHFFNRAGDVGDKIPESLYLKG---SSGRETPGSAIYSPTPSGSMVTSEAQIFNK--PYWLQQAQGHNNG   322
[HPV94]                       PAP--GDCPPLELVTSVIQDGDMVDTGYGAMDFTALQLNKSDVPIDICQSTCKYPDYLGMAAEPYGDSMFFYLRREQLFARHFFNRASAVGDTIPDTLILKS---AGGDQNVGSAVYSPTPSGSMVTSEAQLFNK--PYWLKRAQGHNNG   350
[HPV10NP041746.1]             AQ---GDCPPLELITSPIQDGDMVDTGYGAMDFTALQLNKSDVPIDICQSTCKYPDYLGMAAEPYGDSMFFYLRREQLFARHFFNRASAVGDAIPDTFILKS---NGGGRDVGSAVYSPTPSGSMVTSEAQLFNK--PYWLRRAQGHNNG   349
[HPV10CAA52494.1]             AQ---GDCPPLELITSPIQDGDMVDTGYGAMDFTALQLNKSDVPIDICQSTCKYPDYLGMAAEPYGDSMFFYLRREQLFARHFFNRASAVGDAIPDTFILKS---NGGGRDVGSAVYSPTPSGSMVTSEAQLFNK--PYWLRRAQGHNNG   349
[HPV-10CAA52495.1]            AQ---GDCPPLELITSPIQDGDMVDTGYGAMDFTALQLNKSDVPIDICQSTCKYPDYLGMAAEPYGDSMFFYLRREQLFARHFFNRASAVGDAIPDTFILKS---NGGGRDVGSAVYSPTPSGSMVTSEAQLFNK--PYWLRRAQGHNNG   321
[HPV3]                        SP---GDCPPLELITAPIQDGDMVDTGYGAMDFGNLQSNKSDVPLDICQTTCKYPDYLGMAAEPYGDSMFFYLRKEQLFARHFLNRAGMAGDTVPDALYIKGDSQSGGRDKIGSAVYCPTPSGSMVTSETQLFNK--PYWLRRAQGHNNG   352
HPV28                         QQ---GDCPPLELITAPIQDGDMVDTGYGAMDFAVLQANKSDVPLDICQSTCKYPDYLGMAAEPYGDSMFFYLRKEQLFARHFFNRAGVAGDTIPETLYIKG--QGNGRDVIGSAVYSPTPSGSMISSEAQLFNK--PYWLRRAQGHNNG   322
HPV56CAA52600.1               VTT--GDCPPLALINTPIEDGDMIDTGFGAMDFKVLQESKAEVPLDIVQSTCKYPDYLKMSADAYGDSMWFYLRREQLFARHYFNRAGKVGETIPAELYLKG---SNGREPPPSSVYVATPSGSMITSEAQLFNK--PYWLQRAQGHNNG   356
HPV66                         GNT--GDCPPLALVNTPIEDGDMVDTGFGAMDFKLLQESKAEVPLDIVQSTCKYPDYLKMSADAYGDSMWFYLRREQLFARHYFNRAGNVGEAIPTDLYWKG---GNGRDPPPSSVYVATPSGSMITSEAQLFNK--PYWLQRAQGHNNG   321
[HPV53]                       TTA--GDCPPLELINSPIEDGDMVDTGFGALNFKALQESKSDVPLDIVQSTCKYPDYLKMSADAYGDSMWFYLRREQLFTRHFFNRAGVIGEEIPNDLYIKG---SNGRDPPPSSVYVATPSGSMITSEAQLFNK--PYWLQRAQGHNNG   320
[HPV53CAA52595.1]             TTA--GDCPPLELINSPIEDGDMVDTGFGALNFKALQESKSDVPLDIVQSTCKYPDYLKMSADAYGDSMWFYLRREQLFTRHFFNRAGVIGEEIPNDLYIKG---SNGRDPPPSSVYVATPSGSMITSEAQLFNK--PYWLQRAQGHNNG   320
[HPV30]                       PAQ--GDCPPLELVNSPIQDGDMVDIGFGAMDFKTLQESKSDVPLDISQSTCKYPDYLKMSADAYGDSMWFYLRREQLFARHYFNRAGAIGEQLPSTLYIKG---TNNRDPPPSSVYVATPSGSMVTSEAQLFNK--PYWLQRAQGHNNG   329
[HPV71]                       AAA--GSCPPLELASTVIQDGDMVDTGFGAMDFAALQTNRSDVPLDIVTTTCKY-QIISNGCRAIGDRMFF-LRREQCF-KTFYNRQAHLG-RVPDDYYLKV-HLLPFVLPLQALFMHPHLVGPWLPQSHSCLTSLTGYNVHRAQTMA--   340
[HPV81]                       PAQ--NECPPLEFKNTTIQDGDMVETGYGAIDFKALQENKSEVPLDICNTTCKYPDYLQMAAEPYGDCMFFCLRREQMFARHFFNRHGTMGEALPADLYMKG-AAGSDRITPGSYIYAPTPSGSMVSSDSQLFNK--PYWLQRAQGHNNG   352
[HPV61]                       PRP--TDCPPLEFTNTTIQDGDMVETGYGAIDFAALQENKSEVPLDICTTICKYPDYLQMAAEPYGDCMFFCLRREQMFARHFFNRQGVMGEALPDSYYLKG---ANDKAAPGSYIYSPTPSGSMVSSDSQLFNK--PYWLQRAQGHNNG   323
[HPV72]                       SQP--TDCPPLEFTNSTIQDGDMVETGYGAIDFATLQENKSEVPLDICTTTCKYPDYLQMAAEPYGDCMFFCLRREQMFARHFFNRQGTMGEALPASLYLKG-ASGSDRVTPGSYIYSPTPSGSMVSSDAQLFNK--PYWLQRAQGHNNG   352
[HPV84]                       YRA--GDCPPLQFTNSTIEDGDMVEAGYGAIDFATLQESKSEVPLDLCTTTCKYPDYLQMAAEPYGDCMFFCLRREQMFARHFFNRQGTMGEEVPQSFYLKG---TSSRATLSSSVYAPTPSGSMVSSDSQLFNK--PYWLQKAQGHNNG   325
[HPV86]                       PKP--GDCPPLQFTNSTIQDGDMVEAGYGAMDFDKLQENKSEVPLDICATVCKYPDYLQMAAEPYGDCMFFCLRREQMFARHFFNRQGVMGEPVPETYYLKG--SSGPRGSLSSYVYAPTPSGSMVSSDSQLFNK--PYWLQKAQGHNNS   327
[HPVcand87]                   PKT--GDCPPLQFTNSTIQDGDMVEAGYGAIDFEKLQENKSEVPLDICTTVCKYPDYLQMAAEPYGDCMFFCLRREQMFARHYFNRQGTMGEEVPDTYFLKG-ASSGPRASLSSYVYAPTPSGSMVSSDSQLFNK--PYWLQRAQGHNNG   326
HPV83AAD38974.1|AF151983_7    PQR--GDCPPLQFVNSTIQDGDMVETGYGAMDFATLQESKSEVPIDICTATCKYPDYLQMAAEPYGDCMFFCLRREQMFARHFFNRHGTMGEVLPTSYYIPG-TSANSRNTLTSYIYAPTPSGSLVSSDSQLFNK--PYWLHRAQGHNNG   324
[HPVcand89]                   PQQ--GDCPPLQLLNTTIEDGDMVETGYGAMDFSKLQESKSEVPIDICDSTCKYPDYLQMAAEPYGDCMFFCLRREQMFARHFFNRQGVMGEDLPKTLYMPG-SAANNRNTLTSYVYAPTPSGSMVTSDSQLFNK--PYWLHRAQGHNNG   340
[HPV90]                       VQA--GDCPPIELKSSTIQDGDMIDTGFGALDFAALQTNKSDVPLDIVTTTCKYPDFLQMAAEPYGDLMFFCLRREQMFIRHFYNRAGTMGDAIPDGFVLKG-TSSTSRGTPTSSVYGSTPSGSMVTSESQLFNK--PYWLQRAQGPNNG   325
[HPV57]                       AV---GDCPPLQFTNTTIEDGDMVETGFGALDFAALQSNKSDVPLDICTNICKYPDYLKMAADPYGDSMFFSLRREQMFTRHFFNRGGSMGDALPDELYVKS----STVQTPGSYVYTSTPSGSMVSSEQQLFNK--PYWLRRAQGHNNG   334
[HPV-2]                       AA---GDCPPLQFTNTTIEDGDMVETGFGALDFATLQSNKSDVPLDICTNTCKYPDYLKMAAEPYGDSMFFSLRREQMFTRHFFNLGGKMGDTIPDELYIKS----TSVPTPGSHVYTSTPSGSMVSSEQQLFNK--PYWLRRAQGHNNG   333
[HPV27]                       AA---GDCPPLQFTNSTIEDGDMVETGFGALDFATLQSNRSDVPLDICTNVCKYPDYLKMAAEPYGDSMFFSLRREQMFTRHFFNRAGKMGDTIPDELYIKS----TTISDPGSHVYTSTPSGSMVSSEQQLFNK--PYWLRRAQGHNNG   427
                              .......310.......320.......330.......340.......350.......360.......370.......380.......390.......400.......410.......420.......430.......440.......450
```

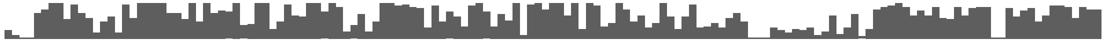

CLUSTAL 2.0.5 MULTIPLE SEQUENCE ALIGNMENT

File: /Users/johnlowe/Desktop/L1protein.ps **Date: Fri May 2 11:55:30 2008**


```
                                        .   **: .**. :           :       :: ** **:::*.::*** : *. : * *:* *.  :*: *:.    *   .* : **::   *  *:          :      ** ::* : :: :* *:.*****
[HPV7]                          ICFGNQLFVTVVDTTRSTNLTLCAATQ-SPTPTPYDNSKFKEYLRHGEEFDLQFIFQLCVITLNAEVMTYIHAMDSSLLDDWNFKIGPPASATLEDTYRFLTNKAIACQRDAP-PKEKE-DPYKKYKFWEVNLTEKFSSQLDQFPLGRKF  467
[HPV-7]                         ICFGNQLFVTVVDTTRSTNLTLCAATQ-SPTPTPYDNSKFKEYLRHGEEFDLQFIFQLCVITLNAEVMTYIHAMDSSLLDDWNFKIGPPASATLEDTYRFLTNKAIACQRDAP-PKEKE-DPYKKYKFWEVNLTEKFSSQLDQFPLGRKF  467
[HPV40]                         ICFGNQLFVTVVDTTRSTNLTLCAATQ-SPTPTPYNNSNFKEYLRHGEEFDLQFIFQLCVITLNAEVMTYIHAMDPTLLEDWNFKIAPPASASLEDTYRFLTNKAIACQRDAP-PKVRE-DPYKKYKFWDVNLTERFSSQLDQFPLGRKF  467
[HPV43]                         ICFGNQLFVTVVDTTRSTNLTLCASTD-PTVPSTYDNAKFKEYLRHVEEYDLQFIFQLCIITLNPEVMTYIHTMDPTLLEDWNFGVSPPASASLEDTYRFLSNKAIACQKNAP-PKERE-DPYKKYTFWDINLTEKFSAQLTQFPLGRKF  495
[HPV91]                         --------VTVVDTTRSTNLTLCASTE-SVLPTTYDNTKFKEYLRHAEEFDLQFIFQLCIITLNPEVMTYIHTMDASLLEDWNFGVSPPATASLEDTYRFLANKAIACQKNVP-PKAKE-DPYKNYTFWDVDLTERFSAQLTQFPLGRKF  434
[HPV6b]                         ICWGNQLFVTVVDTTRSTNMTLCASVT-TS--STYTNSDYKEYMRHVEEYDLQFIFQLCSITLSAEVMAYIHTMNPSVLEDWNFGLSPPPNGTLEDTYRYVQSQAITCQKPTP-EKEKP-DPYKNLSFWEVNLKEKFSSELDQYPLGRKF  463
[HPV6bNP_040304.1]              ICWGNQLFVTVVDTTRSTNMTLCASVT-TS--STYTNSDYKEYMRHVEEYDLQFIFQLCSITLSAEVMAYIHTMNPSVLEDWNFGLSPPPNGTLEDTYRYVQSQAITCQKPTP-EKEKP-DPYKNLSFWEVNLKEKFSSELDQYPLGRKF  463
[HPV11]                         ICWGNHLFVTVVDTTRSTNMTLCASVS-KS--ATYTNSDYKEYMRHVEEFDLQFIFQLCSITLSAEVMAYIHTMNPSVLEDWNFGLSPPPNGTLEDTYRYVQSQAITCQKPTP-EKEKQ-DPYKDMSFWEVNLKEKFSSELDQFPLGRKF  464
[HPV13]                         ICWGNHLFVTVVDTTRSTNMTVCAATT-SSLSDTYKATEYKQYMRHVEEFDLQFIFQLCTIKLTAEVMSYIHTMNPTILEDWNFGLSPPPNGTLEDTYRYVQSQAITCQKPTP-DKEKQ-DPYAGLSFWEVNLKEKFSSELDQYPLGRKF  463
[PCP                            ICWGNTLFVTVVDTTRSTNMTVCASTT-SSPSATYTASEYKQYMRHVEEFDLQFIFQLCSIKLTAEVMAYIHTMNPTVLEEWNFGLSPPPNGTLEDTYRYVQSQAITCQKPTP-DKEKQ-DPYAGLSFWEVNLKEKFSSELDQYPLGRKF  466
[CCP                            ICWGNTLFVTVVDTTRSTNMTVCASTT-SSPSATYTASEYKQYMRHVEEFDLQFIFQLCTIKLTAELMAYIHTMNPTVLEEWNFGLSPPPNGTLEDTYRYVQSQAITCQKPTP-DKEKQ-DPYAGLSFWEVNLKEKFSSELEQYPLGRKF  466
[HPV74]                         ICWGNQLFVTVVDTTRSTNMTVCAPTS-QSPSATYNSSDYKQYMRHVEEFDLQFIFQLCSIKLTAEVMAYIHTMNPTVLEEWNFGLTPPPNGTLEDTYRYVQSQAITCQKPTP-DKAKP-NPYANLSFWEVNLKEKFSSELDQYPLGRKF  491
[HPV44]                         ICWGNQLFVTVVDTTRSTNMTICAATT-QSPPSTYTSEQYKQYMRHVEEFDLQFMFQLCSITLTAEVMAYLHTMNAGILEQWNFGLSPPPNGTLEDKYRYVQSQAITCQKPPP-EKAKQ-DPYAKLSFWEVDLREKFSSELDQYPLGRKF  465
[hpv55]                         ICWGNQLFVTVVDTTRSTNMTICAATT-QSPSTTYNSTEYKQYMRHVEEFDLQFMFQLCSITLTAEVMAYLHTMNPGILEQWNFGLSPPPNGTLEDKYRYVQSQAITCQKPPP-EKAKQ-DPYAKLSFWEVDLREKFSSELDQYPLGRKF  465
HPV34_L1aa                      ICWHNQLFLTVVDTTRSTNFSVCVGTQSTSTTAPYANSNFKEYLRHAEEYDLQFVFQLCKINLTTDVMTYIHSMSSSILEQWNFGLTPPPSGTLEETYRYVTSQAITCQRPQP-PKETE-DPYGKMTFWEVDLKEKFSAELDQFALGRKF  493
[HPV34NP_041812.1]              ICWHNQLFLTVVDTTRSTNFSVCVGTQSTSTTAPYANSNFKEYLRHAEEYDLQFVFQLCKINLTTDVMTYIHSMSSSILEQWNFGLTPPPSGTLEETYRYVTSQAITCQRPQP-PKETE-DPYGKMTFWEVDLKEKFSAELDQFALGRKF  493
[HPV34CAA52560.1]               ICWHNQLFLTVVDTTRSTNFSVCVGTQSTSTTAPYANSNFKEYLRHAEEYDLQFVFQLCKINLTTDVMTYIHSMSSSILEQWNFGLTPPPSGTLEETYRYVTSQAITCQRPQP-PKETE-DPYGKMTFWEVDLKEKFSAELDQFALGRKF  493
HPV73CAA63887.1                 ICWHNQLFLTVVDTTRSTNFSVCVGTQASSSTTTYANSNFKEYLRHAEEFDLQFVFQLCKISLTTEVMTYIHSMNSTILEEWNFGLTPPPSGTLEETYRYVTSQAISCQRPQP-PKETE-DPYAKLSFWDVDLKEKFSAELDQFPLGRKF  468
[HPV54]                         ICWGNQVFLTVVDTTRSTNLTLCATAS-TQ--DSFNNSDFREYIRHVEEYDLQFIFQLCTITLTADVMAYIHGMNPTILEDWNFGITPPATSSLEDTYRFVQSQAIACQKNNA-PAKEKEDPYSKFNFWTVDLKERFSSDLDQFPLGRKF  462
[HPV32]                         ICWGNQVFLTVVDTTRSTNMTVCATVT-TE--DTYKSTNFKEYLRHAEEYDIQFIFQLCKITLSVEVMSYIHTMNPDILDDWNVGVAPPPSGTLEDSYRFVQSQAIRCQAKVT-APEKK-DPFSDYSFWEVNLSEKFSSDLDQFPLGRKF  467
[HPV32CAA52554.1]               ICWGNQVFLTVVDTTRSTNMTVCATVT-TE--DTYKSTNFKEYLRHAEEYDIQFIFQLCKITLSVEVMSYIHTMNPDILDDWNVGVAPPPSGTLEDSYRFVQSQAIRCQAKVT-APEKK-DPFSDYSFWEVNLSEKFSSDLDQFPLGRKF  467
HPV42                           ICWGNQLFLTVVDTTRSTNMTLCATAT-SG--DTYTAANFKEYLRHAEEYDVQFIFQLCKITLTVEVMSYIHNMNPNILEEWNVGVAPPPSGTLEDSYRYVQSEAIRCQAKVT-TPEKK-DPYSDFWFWEVNLSEKFSTDLDQFPLGRKF  467
RhesusPV1                       ICWGNQVFLTVVDTTRSTNMTLCASTA-STVTTPYNNESFKEYLRHVEEFDLQFIFQLCKVTLNTEVMAYIHSMDASILEDWNFGLQPPPSGSLQDTYRFVTSAAITCQKPAP-PKEKE-DPLAKYTFWEVDLKEKFSADLDQFPLGRKF  466
HPV52_L1aa                      ICWGNQLFVTVVDTTRSTNMTLCAEVK-KE--STYKNENFKEYLRHGEEFDLQFIFQLCKITLTADVMTYIHKMDATILEDWQFGLTPPPSASLEDTYRFVTSTAITCQKNTP-PKGKE-DPLKDYMFWEVDLKEKFSADLDQFPLGRKF  498
HPV67_L1aa                      ICWGNQIFVTVVDTTRSTNMTLCSEEK-SE--ATYKNENFKEYLRHVEEYDLQFIFQLCKISLTANVMQYIHTMNPDILEDWQFGLTPPPSGNLQDTYRFVTSQAITCQKTSP-PTAKE-DPLKKYSFWEINLKEKFSADLDQFPLGRKF  497
HPV33_L1aa                      ICWGNQVFVTVVDTTRSTNMTLCTQVT-SD--STYKNENFKEYIRHVEEYDLQFVFQLCKVTLTAEVMTYIHAMNPDILEDWQFGLTPPPSASLQDTYRFVTSQAITCQKTVP-PKEKE-DPLGKYTFWEVDLKEKFSADLDQFPLGRKF  467
HPV58_L1aa                      ICWGNQLFVTVVDTTRSTNMTLCTEVT-KE--GTYKNDNFKEYVRHVEEYDLQFVFQLCKITLTAEIMTYIHTMDSNILEDWQFGLTPPPSASLQDTYRFVTSQAITCQKTAP-PKEKE-DPLNKYTFWEVNLKEKFSADLDQFPLGRKF  493
HPV35                           ICWSNQLFVTVVDTTRSTNMSVCSAVS-SSD-STYKNDNFKEYLRHGEEYDLQFIFQLCKITLTADVMTYIHSMNPSILEDWNFGLTPPPSGTLEDTYRYVTSQAVTCQKPSA-PKPKD-DPLKNYTFWEVDLKEKFSADLDQFPLGRKF  469
HPV31_L1aa                      ICWGNQLFVTVVDTTRSTNMSVCAAIA-NSD-TTFKSSNFKEYLRHGEEFDLQFIFQLCKITLSADIMTYIHSMNPAILEDWNFGLTTPPSGSLEDTYRFVTSQAITCQKTAP-QKPKE-DPFKDYVFWEVNLKEKFSADLDQFPLGRKF  469
HPV16NP_041332.1                ICWGNQLFVTVVDTTRSTNMSLCAAIS-TSE-TTYKNTNFKEYLRHGEEYDLQFIFQLCKITLTADVMTYIHSMNSTILEDWNFGLQPPPGGTLEDTYRFVT-QAIACQKHTP-PAPKEDDPLKKYTFWEVNLKEKFSADLDQFPLGRKF  494
HPV16_L1aa                      ICWGNQLFVTVVDTTRSTNMSLCAAIS-TSE-TTYKNTNFKEYLRHGEEYDLQFIFQLCKITLTADVMTYIHSMNSTILEDWNFGLQPPPGGTLEDTYRFVT-QAIACQKHTP-PAPKEDDPLKKYTFWEVNLKEKFSADLDQFPLGRKF  494
[HPV16eaAF534061]               ICWGNQLFVTVVDTTRSTNMSLCAAIS-TSE-TTYKNTNFKEYLRHGEEYDLQFIFQLCKITLTADVMTYIHSMNSTILEDWNFGLQPPPGGTLEDTYRFVTSQAIACQKHTP-PAPKE-DPLKKYTFWEVNLKEKFSADLDQFPLGRKF  494
[HPV16gerAAQ10719.1]            ICWGNQLFVTVVDTTRSTNMSLCAAIS-TSE-TTYKNTNFKEYLRHGEEYDLQFIFQLCKITLTADVMTYIHSMNSTILEDWNFGLQPPPGGTLEDTYRFVTSQAIACQKHTP-PAPKE-DPLKKYTFWEVNLKEKFSADLDQFPLGRKF  494
[HPV16af2]                      ICWGNQLFVTVVDTTRSTNMSLCAAIS-TSE-PTYKNTNFKEYLRHGEEYDLQFIFQLCKITLTADVMTYIHSMNSTILEDWNFGLQPPPGGTLEDTYRFVTSQAIACQKHTP-PAPKE-DPLKKYTFWEVNLKEKFSADLDQFPLGRKF  494
[HPV16asamAF402678]             ICWGNQLFVTVVDTTRSTNMSLCAAIS-TSE-PTYKNTNFKEYLRHGEEYDLQFIFQLCKITLTADVMSYIHSMNSTILEDWNFGLQPPPGGTLEDTYRFVTSQAIACQKHTP-PAPKE-DPLKKYTFWEVNLKEKFSADLDQFPLGRKF  494
[HPV16aa]                       ICWGNQLFVTVVDTTRSTNMSLCAAIS-TSE-PTYKNTNFKEYLRHGEEYDLQFIFQLCKITLTADVMTYIHSMNSTILEDWNFGLQPPPGGTLEDTYRFVT-QAIACQKHTP-PAPKEDDPLKKYTFWEVNLKEKFSADLDQFPLGRKF  494
[HPV16af1]                      ICWGNQLFVTVVDTTRSTNMSLCAAIS-TSE-TTYKNTNFKEYLRHGEEYDLQFIFQLCKITLTADVMTYIHSMNSTILEDWNFGLQPPPGGTLEDTYRFVTSQAIACQKHTP-PAPKE-DPLKKYTFWEVNLKEKFSADLDQFPLGRKF  494
HPV51                           ICWNNQLFITCVDTTRSTNLTISTAT--AAVSPTFTPSNFKQYIRHGEEYELQFIFQLCKITLTTEVMAYLHTMDPTILEQWNFGLTLPPSASLEDAYRFVRNAATSCQKDTP-PQAKP-DPLAKYKFWDVDLKERFSLDLDQFALGRKF  468
[HPV82]                         ICWNNQLFITCVDTTKSTNLTISTAVT-PSVAQTFTPANFKQYIRHGEEYELQFIFQLCKITLTTEIMAYLHTMDSTILEQWNFGLTLPPSASLEDAYRFVKNAATSCHKDSP-PQAKE-DPLAKYKFWNVDLKERFSLDLDQFALGRKF  469
HPV26NP_041787.1                ICWGNQLFVTCVDTTRSTNLTISTLSA-ASASTPFKPSDYKQFIRHGEEYELQFIFQLCKITLTTDVMAYIHLMNASILEDWNFGLTLPPTASLEDAYRFIKNSATTCQRNAP-PVPKE-DPFQKFKFWDVDLKEKFSIDLDQFPLGRKF  469
HPV26bCAA52535.1                ICWGNQLFVTCVDTTRSTNLTISTLSA-ASASTPFKPSDYKQFIRHGEEYELQFIFQLCKITLTTDVMAYIHLMNASILEDWNFGLTLPPTASLEDAYRFIKNSATTCQRNAP-PVPKE-DPFQKFKFWDVDLKEKFSIDLDQFPLGRKF  469
[HPV69]                         ICWGNQLFVTCVDTTRSTNLTISTVSA-QSASATFKPSDYKQFIRHGEEYELQFIFQLCKITLTTDVMAYIHTMNSTILENWNFGLTLPPTASLEDAYRFIKNSATTCQRDAP-AQPKE-DPFSKLKFWDVDLKEKFSIDLDQFPLGRKF  470
HPV18_X05015                    VCWHNQLFVTVVDTTPSTNLTICASTQ-SPVPGQYDATKFKQYSRHVEEYDLQFIFQLCTITLTADVMSYIHSMNSSILEDWNFGVPPPPTTSLVDTYRFVQSVAITCQKDAA-PAENK-DPYDKLKFWNVDLKEKFSLDLDQYPLGRKF  530
HPv18_NC001357                  VCWHNQLFVTVVDTTPSTNLTICASTQ-SPVPGQYDATKFKQYSRHVEEYDLQFIFQLCTITLTADVMSYIHSMNSSILEDWNFGVPPPPTTSLVDTYRFVQSVAITCQKDAA-PAENK-DPYDKLKFWNVDLKEKFSLDLDQYPLGRKF  530
HPV45_L1aa                      ICWHNQLFVTVVDTTRSTNLTLCASTQ-NPVPSTYDPTKFKQYSRHVEEYDLQFIFQLCTITLTAEVMSYIHSMNSSILENWNFGVPPPPTTSLVDTYRFVQSVAVTCQKDTT-PPEKQ-DPYDKLKFWTVDLKEKFSSDLDQYPLGRKF  498
HPV59_L1aa                      ICWHNQLFLTVVDTTRSTNLSVCASTT-SSIPNVYTPTSFKEYARHVEEFDLQFIFQLCKITLTTEVMSYIHNMNTTILEDWNFGVTPPPTASLVDTYRFVQSAAVTCQKDTA-PPVKQ-DPYDKLKFWPVDLKERFSADLDQFPLGRKF  469
HPVcand85_L1aa                  ICWHNQLFITVVDTTRSTNLTLSTATT-NPVPSIYEPSKFKEYTRHVEEYDLQFIFQLCKITLTTDVMSYIHNMDPTILDSWNFGVSPPPSASLVDTYRFLQSSAITCQKDVV-VPQKK-DPYEKLKFWNVDLKEHFSSDLDQFPLGRKF  469
HPV68_L1aa                      ICWHNQLFLTVVDTTRSTNFTLSTTTD-STVPAVYDSNKFKEYVRHVEEYDLQFIFQLCTITLSTDVMSYIHTMNPAILDDWNFGVAPPPSASLVDTYRYLQSAAITCQKDAP-APVKK-DPYDGLNFWNVDLKEKFSSELDQFPLGRKF  469
HPV39_L1aa                      ICWHNQLFLTVVDTTRSTNFTLSTSIE-SSIPSTYDPSKFKEYTRHVEEYDLQFIFQLCTVTLTTDVMSYIHTMNSSILDNWNFAVAPPPSASLVDTYRYLQSAAITCQKDAP-APEKK-DPYDGLKFWNVDLREKFSLELDQFPLGRKF  468
HPV70_L1aa                      ICWHNQLFITVVDTTRSTNFTLSACTE-TAIPAVYSPTKFKEYTRHVEEYDLQFIFQLCTITLTADVMAYIHTMNPAILDNWNIGVTPPPSASLVDTYRYLQSAAIACQKDAP-TPEKK-DPYDDLKFWNVDLKEKFSTELDQFPLGRKF  467
HPV29                           ICWANQVFLTVVDTTRSTNMSLCATTE-SQPLTTYDATKIKEYLRHGEEYDLQFIFQLCKVTLTPEIMAYLHTMNSALLEDWNFGLTLPPSTSLEDTYRFVTSSAITCQKDLA-PTEKQ-DPYAKLNFWDVDLKDRFTLDLSQFPLGRKF  469
[HPV77]                         ICWGNQVFLTVVDTTRSTNMSLSASTE-SQTPSTYDATKIKEYLRHGEEYDLQFIFQLCKVTLTPEIMAYLHTMNTALLEDWNFGLTLPPSTSLEDTYRFVTSSAITCQKDVA-PTEKQ-DPYAKLNFWDVDLKDRFTLDLSQFPLGRKF  469
[HPV94]                         ICWGNQMFVTVVDTTRSTNLSLCVPSD-ASTATTYDATKFKEFLRHGEEYDLQFIFQLCKVTLTPEIMAYLHTMNSSLLEDWNFGLTLPPSTSLEDTYRFLSSSAITCQKEAP-PTEKQ-DPYAKLNFWDVDLKDRFTLDLSQFPLGRKF  497
[HPV10NP041746.1]               ICWANQLFVTVVDTTRSTNMCLCVPSE-ASPATTYDATKFKEYLRHGEEYDLQFIFQLCKVTLTPDIMAYLHTMNSSLLEDWNFGLTLPPSTSLEDTYRFLSSSAITCQKDTP-PTEKQ-DPYAKLNFWDVDLKDRFSLDLSQFPLGRKF  496
[HPV10CAA52494.1]               ICWANQLFVTVVDTTRSTNMCLCVPSE-ASPATTYDATKFKEYLRHGEEYDLQFIFQLCKVTLTPDIMAYLHTMNSSLLEDWNFGLTLPPSTSLEDTYRFLSSSAITCQKDTP-PTEKQ-DPYAKLNFWDVDLKDRFSLDLSQFPLGRKF  496
[HPV-10CAA52495.1]              ICWANQLFVTVVDTTRSTNMCLCVPSE-ASPATTYDATKFKEYLRHGEEYDLQFIFQLCKVTLTPDIMAYLHTMNSSLLEDWNFGLTLPPSTSLEDTYRFLSSSAITCQKDTP-PTEKQ-DPYAKLNFWDVDLKDRFSLDLSQFPLGRKF  468
[HPV3]                          ICWANQLFVTVVDTTRSTNMTLCVSTE-TS--ATYDATKFKEYLRHGEEYDLQFIFQLCKVTLTPEIMAYLHTMNSTLLEDWNFGLTLPPSTSLEDTYRFLTSSAITCQKDAP-PTEKQ-DPYAKLNFWDVDLKDRFSLDLSQFPLGRKF  497
HPV28                           ICWANQLFVTVVDTTRSTNMTLCVSTD-SS--ATYDASKFKEYLRHGEEYDLQFIFQLCKVTLTPDIMAYLHTMNNSLLEDWNFGLTLPPSTSLEDTYRFISSSAITCQKDAS-PTTKE-DPYAKLNFWEVDLKDRFSLDLSQFPLGRKF  467
HPV56CAA52600.1                 ICWGNQLFVTVVDTTRSTNMTISTATE-QL--SKYDARKINQYLRHVEEYELQFVFQLCKITLSAEVMAYLHNMNANLLEDWNIGLSPPVATSLEDKYRYVRSTAITCQREQP-PTEKQ-DPLAKYKFWDVNLQDSFSTDLDQFPLGRKF  501
HPV66                           ICWGNQVFVTVVDTTRSTNMTINAAKS-TL--TKYDAREINQYLRHVEEYELQFVFQLCKITLTAEVMAYLHNMNNTLLDDWNIGLSPPVATSLEDKYRYIKSTAITCQREQP-PAEKQ-DPLAKYKFWEVNLQDSFSADLDQFPLGRKF  466
[HPV53]                         ICWNNQLFVTVVDTTRNTNMTLSATTQ-SM--STYNSKQIKQYVRHAEEYELQFVFQLCKISLSAEVMAYLHTMNSTLLEDWNIGLSPPVATSLEDKYRYVKSAAITCQKDQP-PPEKQ-DPLSKYKFWEVNLQNSFSADLDQFPLGRKF  465
[HPV53CAA52595.1]               ICWNNQLFVTVVDTTRNTNMTLSATTQ-SM--STYNSKQIKQYVRHAEEYELQFVFQLCKISLSAEVMAYLHTMNSTLLEDWNIGLSPPVATSLEDKYRYVKSAAITCQKDQP-PPEKQ-DPLSKYKFWEVNLQNSFSADLDQFPLGRKF  465
[HPV30]                         ICWGNQVFVTVVDTTRNTNMTISATTQ-TL--STYNSSQIKQYVRHVEEYELQFVFQLCKISLSAETMAYLHTMNSTLLEGWNIGLSPPAATSLEDKYRYVKSLAITCQKDQP-PAEKE-DPLAKYKFWDVNLQDSFSADLDQFPLGRKF  474
[HPV71]                         -FVGQSAFCNSCDTSRSTNMSICATKT-VE--STYKASSFMEYLRHGEEFDLQFIFQLCVINLTAEIMAYLHGMDATLLEDWNFGSLPPPTASLGDTYRFLQSQAITCQKNSPPPAEKK-DPYADLTFWEVDLKERFSLELDQFPLGRKF  485
[HPV81]                         ICWFNEMFVTVVDTTRSTNFTICTATS-AA--AEYKASNFKEFLRHTEEYDLQFIFQLCKIQLTPEIMAYLHNMNKALLDDWNFGVLPPPSTSLDDTYRFLQSRAITCQKGAAAPAPKE-DPYADMSFWTVDLKDKLSTDLDQFPLGRKF  498
[HPV61]                         ICWFNELFVTVVDTTRSTNLTICTATS-PPV-SEYKATSFREYLRHTEEFDLQFIFQLCKIHLTPEIMAYLHNMNKALLDDWNFGVVPPPSTSLEDTYRFLQSRAITCQKGAAAPPPKE-DRYAKLSFWTVDLRDKFSTDLDQFPLGRKF  470
[HPV72]                         ICWFNELFVTVVDTTRSTNVTICTATA-SSV-SEYTASNFREYLRHTEEFDLQFIFQLCKIHLTPEIMAYLHNMNKALLDDWNFGVVPPPSTSLDDTYRFLQSRAITCQKGAATPPPKE-DPYANLSFWTVDLKDKFSTDLDQFPLGRKF  499
[HPV84]                         ICWFNQLFVTVVDTTRSTNFTISAATN-TE--SEYKPTNFKEYLRHVEEYDLQFIFQLCKVRLTPEVMSYLHTMNDSLLDEWNFGVVPPPSTSLDDTYRYLQSRAITCQKGAAAAKPKD-DPYAGMSFWDVDLKDKFSTDLDQYPLGRKF  471
[HPV86]                         ICWFNQLFVTVVDTTRSTNFTISAATQ-KA--SEYDPSKFNEYLRHAEEYDLQFIFQLCKVRLTPEIMSYLHSMNESLLDEWNFGVVPPPSTSLEDTYRFLQSRAITCQKGATAPQPKE-DPYAGMVFWEVDLKDKLSTDLDQFPLGRKF  473
[HPVcand87]                     ICWFNQLFVTVVDTTRSTNFTISAATQ-TT--TEYDPTKFKEYLRHVEEYDLQFIFQLCKVRLTPEVMAYLHTMNESLLDEWNFGVVPPPSTSLDDTYRYLQSRAITCQKGSAATKPKE-DPYTGMLFWEVDLKDKLSTDLDQFPLGRKF  472
HPV83AAD38974.1|AF151983_7      ICWFNELFVTVVDTTRSTNITISAAAT-QA--NEYTASNFKEYLRHTEEYDLQVILQLCKIHLTPEIMAYLHSMNEHLLDEWNFGVLPPPSTSLDDTYRYLQSRAITCQKGPSAPAPKK-DPYDGLVFWEVDLKDKLSTDLDQFPLGRKF  470
[HPVcand89]                     ICWFNELFVTVVDTTRSTNLTICAASQ-SG--TEYSSTRFKEYLRHTEEYDLQFIFQLCKIHLTPEIMSYLHNMNDTLLDEWNFGVIPPPSTSLDDTYRFLTSRAITCQKGTAAPEPKK-DPYDKLSFWDVDLKERLSTDLDQFPLGRKF  486
[HPV90]                         ICWGNQLFVTVVDTTRSTNMTICATQT-PS--DTYKASNFKEYMRHGEEFDLQFIFQLCVINLTTEVMAYLHGMNATLLEDWNFGSLPPPTASLGDTYRFLQSQAISCQKPSAVPTENK-DPYAGLTFWEVDLSERFSLELDQFPLGRKF  471
[HPV57]                         MCWGNRIFLTVVDTTRSTNVSLCATVT-TE--TNYKASNYKEYLRHMEEYDLQFIFQLCKITLTPEIMAYIHNMDARLLEDWNFGVPPPPSASLQDTYRYLQSQAITCQKPTP-PKTPT-DPYATMTFWDVDLSESFSMDLDQFPLGRKF  479
[HPV-2]                         MCWGNRVFLTVVDTTRSTNVSLCATEA-SD--TNYKATNFKEYLRHMEEYDLQFIFQLCKITLTPEIMAYIHNMDPQLLEDWNFGVPPPPSASLQDTYRYLQSQAITCQKPTP-PKTPT-DPYASLTFWDVDLSESFSMDLDQFPLGRKF  478
[HPV27]                         MCWGNRIFLTVVDTTRSTNVSLCAAEV-SDN-TNYKATNFKEYLRHMEEYDLQFIFQLCKITLTPEIMAYIHNMDPQLLEDWNFGVPPPPSASLQDTYRYLQSQAITCQKPTP-PKTPT-DPYANMTFWDVDLRESFSMDLDQFPLGRKF  573
                                .......460.......470.......480.......490.......500.......510.......520.......530.......540.......550.......560.......570.......580.......590.......600
```

CLUSTAL 2.0.5 MULTIPLE SEQUENCE ALIGNMENT


```
                             ::*            :
[HPV7]                       LMQAG---LRTGPKFKSRKRPAPTSSSSSGSVTPKRK---KTKR--   505
[HPV-7]                      LMQAG---LRTGPKFKSRKRPAPTSSSSSGSVTPKRK---KTKR--   505
[HPV40]                      LMQAG---VRAGPRFKSRKRPAPSSSSSSKPVTPKRK---KTKR--   505
[HPV43]                      VMQAG---LRPKPKLKTVKRSAPSSSTSAPA--SKRK---KTKR--   531
[HPV91]                      VMQAG---LRPRPKLKSGKRAAPSSSSAPAS---KRK---KTKR--   469
[HPV6b]                      LLQSG---YRGRSSIRTGVKRPAVSKASAAP-KRKRA---KTKR--   500
[HPV6bNP_040304.1]           LLQSG---YRGRSSIRTGVKRPAVSKASAAP-KRKRA---KTKR--   500
[HPV11]                      LLQSG---YRGRTSARTGIKRPAVSKPSTAP-KRKRT---KTKK--   501
[HPV13]                      LLQTG---VQSRSPIRVGRKRAASTSTATPT---TRK---KAKRK-   499
[PCP                         LLQTG---VQTTSFARAGTKRAASTSSSTPT---TRK---RVKRK-   502
[CCP                         LLQTG---VQSTSLARAGTKRAASTSTATP----TRK---KVKRK-   501
[HPV74]                      LLQTG---VQARSSVRVSKKRSAPTAPSSAT---KQK---RSRKR-   527
[HPV44]                      LLQTG---VQARSSVRVGRKRPASAATSSS----KQK---RSRKK-   500
[hpv55]                      LLQTG---VQARSSVRVGRKRPASAATSSSS-KPKRS---RKK---   501
HPV34_L1aa                   LLQLG---MRARPRLQASKRSAPSSSSTAP----KKK---RAKRI-   528
[HPV34NP_041812.1]           LLQLG---MRARPRLQASKRSAPSSSSTAP----KKK---RAKRI-   528
[HPV34CAA52560.1]            LLQLG---MRARPRLQASKRSAPSSSSTAP----KKK---RAKRI-   528
HPV73CAA63887.1              LLQLG---MRARPKLQASKRSASATTSATP----KKK---RAKRI-   503
[HPV54]                      LLQAG---LRARPRLRPVKRAAPSSSKGTAR---KRA---KTKR--   497
[HPV32]                      LLQAG---LRARPKLTAVKRTASSSQKSSSP--AKRR---KTRK--   503
[HPV32CAA52554.1]            LLQAG---LRARPKLTAVKRTASSSQKSSSP--AKRR---KTRK--   503
HPV42                        LLQAG---LRARPKLSVGKRKASTAKSVSSA---KRK---KTHK--   502
RhesusPV1                    LLQAG---MRARPTLRAPKRTASSTSSSSPR---KRK---RTKR--   501
HPV52_L1aa                   LLQAG---LQARPKL---KRPASSAPRTST----KKK---KVKR--   529
HPV67_L1aa                   LLQAG---FTAKPKL---KRSSPSSSSSSSA---KRK---KVKR--   529
HPV33_L1aa                   LLQAG---LKAKPKL---KRAAPTSTRTSSA---KRK---KVKK--   499
HPV58_L1aa                   LLQSG---LKAKPRL---KRSAPTTRAPST----KRK---KVKK--   524
HPV35                        LLQAG---LKARPNFRLGRRAAPASTSKKSS--TKRR---KVKS--   505
HPV31_L1aa                   LLQAG---YRARPKFKAGKRSAPSASTTTPA---KRK---KTKK--   504
HPV16NP_041332.1             LLQAG---LKAKPKFTLGKRKATPTTSSTST-TAKRK---KRKL--   531
HPV16_L1aa                   LLQAG---LKAKPKFTLGKRKATPTTSSTST-TAKRK---KRKL--   531
[HPV16eaAF534061]            LLQAG---LKAKPKFTLGKRKATPTTSSTST-TAKRK---KRKL--   531
[HPV16gerAAQ10719.1]         LLQAG---LKAKPKFTLGKRKATPTTSSTST-TAKRK---KRKL--   531
[HPV16af2]                   LLQAG---FKAKPKFTLGKRKATPTTSSTST-TAKRK---KRKL--   531
[HPV16asamAF402678]          LLQAG---FKAKPKFTLGKRKATPTTSSTST-TAKRK---KRKL--   531
[HPV16aa]                    LLQAG---FKAKPKFTLGKRKATPTTSSTST-TAKRK---KRKL--   531
[HPV16af1]                   LLQAG---FKAKPKFTLGKRKATPTTSSTST-TAKRK---KRKL--   531
HPV51                        LLQVG---VQRKPRPGL-KRPASSASSSSSS-SAKRK---RVKK--   504
[HPV82]                      LLQIG---AQRKPRPGL-KRPAPSSSASSSA---KRK---RVKK--   503
HPV26NP_041787.1             MLQAG---IQRRPKLGT-KRPLSSTSSST-----KRK---KRKLTK   503
HPV26bCAA52535.1             MLQAG---IQRRPKLGT-KRPLSSTSSSTKR---KKR---KLTK--   503
[HPV69]                      MLQAG---IQRRPKLGT-KRPASSLSASSSSTTRKKR---KLTK--   507
HPV18_X05015                 LVQAG---LRRKPTIGPRKRSAPSATTSSKPAKRVRV---RARK--   568
HPv18_NC001357               LVQAG---LRRKPTIGPRKRSAPSATTSSKPAKRVRV---RARK--   568
HPV45_L1aa                   LVQAG---LRRRPTIGPRKRPAASTSTASTASRPAKRVRIRSKK--   539
HPV59_L1aa                   LLQLG---ARPKPTIGPRKRAAPAPTSTPSPKRVKRR---KSSRK-   508
HPVcand85_L1aa               LLQAG---LRPKPTIGPRKRVASTSTATRPS---KRK---RTAK--   504
HPV68_L1aa                   LLQAG---VRRRPTIGPRKRTATATTTSTSK--HKRK---RVSK--   505
HPV39_L1aa                   LLQAR---VRRRPTIGPRKRPAASTSSSSAT-KHKRK---RVSK--   505
HPV70_L1aa                   LLQVG---ARRRPTIGPRKRPASAKSSSSAS-KHKRK---RVSK--   504
HPV29                        LLQIG---ARRRSVVPSRKRRTTTTAPTPA----KRK---RSKK--   503
[HPV77]                      LLQIG---ARRRSVVPSRKRRAPTPSPAST----KRK---RSKK--   503
[HPV94]                      LLQLG---VRSRSAISVRKRSATAASGSTAA---KRK---RTKK--   532
[HPV10NP041746.1]            LLQLG---VRSRSAVSVRKRPATSATGSTAA---KRK---RTKK--   531
[HPV10CAA52494.1]            LLQLG---VRSRSAVSVRKRPATSATGSTAA---KRK---RTKK--   531
[HPV-10CAA52495.1]           LLQLG---VRSRSAVSVRKRPATSATGSTAA---KRK---RTKK--   503
[HPV3]                       LMQLG---VGTRSSISVRKRSATTTSRTAAA---KRK---RTKK--   532
HPV28                        LMQLG---VGARSSVSVRKRPASTTRGSSAA---KRK---RAKK--   502
HPV56CAA52600.1              LMQLG---TRSKPAVATSKKRSAPTSTSTPA---KRK---RR----   534
HPV66                        LMQLGPRPPRPKASVSASKRRAAPTSSSSSP---AKR---KKR---   503
[HPV53]                      LMQVG---VRTKPPVSSKKRSASTTSTSAPS---SKR---KRK---   499
[HPV53CAA52595.1]            LMQVG---VRTKPPVSSKKRSASTTSTSAPS---SKR---KRK---   499
[HPV30]                      LMQLG---VRTKPSTTTKKRSAPSSSTSTPS---AKR---KRR---   508
[HPV71]                      LLQSG---TRSRPTALSRKRVAASTTSTAP----KRK---RVKRSR   521
[HPV81]                      LLQSG---SRTRSFAVSRKRPASARTPTTSA---KRK---RKK---   532
[HPV61]                      LLQAG-----PRSVSVSRKRAAPSSTPTSSP-ATKRK---KRKQ--   505
[HPV72]                      LLQVG-----SRAVSVSRKRAAPPSSTSTPA-PTKRK---KRKK--   534
[HPV84]                      LLQSA-----PRSTLVSRKRTASASTPPAS----KRR---KAKK--   503
[HPV86]                      LLQSA-----PRVSHVSRKRPASTSTASSS----KRR---KTKK--   505
[HPVcand87]                  LLQSA-----PRVSRVSRKRPASTSTASTS----KRR---KAKK--   504
HPV83AAD38974.1|AF151983_7   LLQLG-----PRSVSVSRKRPASTAPSAPSK---KKV---KRRK--   503
[HPVcand89]                  LLQLG-----GRPSSVPRKRAAPVSTSKAP----KRK---KAKR--   518
[HPV90]                      LLQRG---TRVRSSPVSRKRPAPSTAPST-----KRK---RSKRS-   505
[HPV57]                      LLQRG-------ATPTVSRKRAAATAAAPTA---KRK---KVRR--   510
[HPV-2]                      LLQRG-----AMPTV-SRKRAAVSGTTPPTS---KRK---RVRR--   510
[HPV27]                      LLQRG---T---TPTVSRKRTAVGRGH-------------------   594
                             .......610.......620.......630.......640......
```

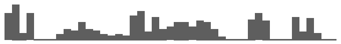

CLUSTAL 2.0.5 MULTIPLE SEQUENCE ALIGNMENT

File: /Users/johnlowe/Desktop/L2muscle.ps **Date: Fri May 2 13:22:30 2008**


```
                                                  ::            ****.*::* :** :** * *:: *::  * *:.:*:: .   ::* ****:.:*:***:** *:         .            :*.         .:.. :.*:: ::*:: .:   .
[HPVcand89]                                -MTHPVR--RR------------KRASVTDLYRTCKATGTCPSDVIPKVEGNTLADKILKWASLGVFFGGLGIGTSSGTGGRTGYVPL--G-TR--PPTVVDVGPTARPPVVI-----EPVGAGEPSIVNLVEDSSIVESGAVLPN---F 122
[HPV81]                                    -MPKVLH--RR------------KRASVTDLYRSCKVSGTCPSDVVPKVEGNTLADKILKWASLGVFFGGLGIGTASGSGGRTGYIPI--G-GR--PPSVVDIGPVSRPPVVI-----DPVGAADPSIVTLVEESSVIEAGATHPN---F 122
[HPV61]                                    ---MALK--RR------------KRASATDLYRTCKQSGTCPPDVVPKVEGDTLADRILKWASLGVFFGGLGIGTGSGTGGRTGYVPI--G-TR--PPTVVDIGPVSRPPVVI-----DPVGAADPSIVTLVEESSVIEAGATVPT---F 120
[HPV72]                                    -MTQAVR--RR------------KRASATDLYRTCKQAGTCPPDVIPKVEGDTLADRFLKWASLGVFFGGLGIGTGSGTGGRTGYVPI--G-TR--PPTVVDIGPTTRPPVVI-----EPVGAADPSIVTLVEESSVVEAGATVPT---F 122
[HPV83]                                    -MSHVHR--RR------------KRASATDLYRTCKVAGTCPPDIVPKIEGDTWADRFLKWASLGLFFGRLGIGTSSGTGGRTGYVPL--G-TR--PPTVVDVGPTARPPVVI-----EPVGATEPSIVNLVEDSSVIESGATIPT---F 122
[HPV84]                                    -MPKVLK--RR------------KRASATDLYRTCKATGTRPADVIPKVEGDTWADRFLKWASLGVFFGGLGIGTSSGTGGRTGYIPL--G-TR--PPTVVDVGPTARPPVVI-----EPVGAADPSIVTLVEDSSVINAGAPFPN---F 122
[HPV86]                                    -MPKVKS--RR------------KRASATDLYRTCKASGTCPADVVPKVEGDTLADRILKWASLGVFFGGLGIGTSSGSGGRTGYVPL--G-TR--PPTVVDVGPTARPPVVI-----EPVGATDPSIVTLVEDSSVINAGAPLPN---F 122
[HPVcand87]                                MPKS-----RR------------KRASATDLYRTCKVTGTCPADVVPKVEGDTLADRILKWASLGVFFGGLGIGTSSGTGGRTGYIPL--G-TR--PPTVVDVGPTARPPVVI-----EPVGAADPSIVTLVEDSSVINSGAPFPN---F 120
[HPV71]                                    --MPRAR--RR------------KRASVTQLYQSCKLTGTCPPDVINKVEHNTLADKILRWGSLGIFLGGLGIGTGSGTGGRTGYIPI--G-TR--PPTVVDVGPPARPPVVI-----ETVGASDPSIVSLVEDSSIIEAGAPYPN---F 121
[HPV90]                                    --MHRSK--RR------------KRASATQLYQTCKLAGTCPSDVINKVEHTTLADKILQWGSLGVFFGGLGIGTGSGTGGRTGYIPI--G-TR--PPTVVDVGPPARPPVVI-----EPVGASDPSIVTLVEDSSIIASGSPHPT---F 121
[HPV10]gil9627262lreflNP_041745.1l         MVAQRAR--RR------------KRASATQLYRTCKASGTCPPDVIPKVEGTTLADRILQWGSLGVYLGGLGIGTGSGTGGRTGYVPI--S-TR--PGTVVDVSVPARPPVVI-----EPVGPSDPSIVNLLEDSSIINSGSTIPT---F 123
[Hpv10]gil396907lemblCAA52493.1l           MVAQRAR--RR------------KRASATQLYRTCKASGTCPPDVIPKVEGTTLADRILQWGSLGVYLGGLGIGTGSGTGGRTGYVPI--S-TR--PGTVVDVSVPARPPVVI-----EPVGPSDPSIVNLLEDSSIINSGSTIPT---F 123
[Hpv94]                                    MVAHRAR--RR------------KRASATQLYRTCKASGTCPPDVIPKVEGTTLADKLLQWGSLGIFLGGLGIGTGSGTGGRTGYVPV--S-TR--PGTVVDVSVPVKPPVVI-----DPVGPSDPSIVNLLEDSSIVNAGSTIPT---F 123
[Hpv3]                                     MVAHRAR--RR------------KRASATQLYRTCKAAGTCPPDVIPKVEGTTLADRILQWGSLGVYLGGLGIGTGSGTGGRTGYAPI--S-TR--PGTVVDVSVPAKPPVVI-----EPVGPSDPSIVNLLEDSSIINSGSTIPT---F 123
hpv28                                      MVAHRAR--RR------------KRASATQLYRTCKAAGTCPPDVIPKVEGTTLADRILQWGGLGIYLGGLGIGTGSGTGGRTGYVPL--S-TR--PGTVVDVSVPARPPVVI-----EPVGPSDPSIVNLLEDSSIINSGSTVPT---F 123
hpv29                                      MVAHRAR--RR------------KRASATELYKTCKVAGTCPPDVIPKVEGTTLADRILQWGSLGVYLGGLGIGTGSGTGGRTGYVPV--G-TR--PGTVVDVSIPTRPPVVI-----EPVGPSDPSIVTLLEESSVINSGATIPT---F 123
[Hpv77]                                    MVAHRAR--RR------------KRASATELYKTCKAAGTCPPDVIPKVEGTTLADRILQWGSLGVYLGGLGIGTGTGTGGRTGYVPI--G-TR--PGTVVDVSVPTRPPVVV-----EPVGPTDPSIVTLLEESSVIDSGASIPT---F 123
[HPV56]                                    MVAHRAT--RR------------KRASATQLYKTCKLSGTCPEDVVNKIEQKTWADKILQWGSLFTYFGGLGIGTGTGSGGRAGYVPL--G-SR--PSTIVDVT-PARPPIVV-----ESVGPTDPSIVTLVEESSVIESGAGIPN---F 122
[hpv66]gi|1020296|gb|AAA79504.1|           MVAHRAT--RR------------KRASATQLYKTCKLSGTCPEDVINKVEQKTWADRILQWGSLFTYFGGLGIGTGSGSGGRAGYVPL--G-SR--PSTIVDVT-PARPPIVV-----ESVGPTDPSIVTLVEESSVINSGAGVPN---F 122
[HPV53]gi|9627381|ref|NP_041847.1|         MVAHRAR--RR------------KRASATQLYQTCKQSGTCPEDVINKIEHKTWADKILQWGSLFTFFGGLGIGTGSGTGGRTGYIPL--G-TR--PSTVVDVT-PARPPIVV-----ESVGPTDPSIVTLVEESSVIESGASFPN---F 122
[HPV53]gi|397051|emb|CAA52594.1|           MVAHRAR--RR------------KRASATQLYQTCKQSGTCPEDVINKIEHKTWADKILQWGSLFTFFGGLGIGTGSGTGGRTGYIPL--G-TR--PSTVVDVT-PARPPIVV-----ESVGPTDPSIVTLVEESSVIESGASFPN---F 122
[HPV30]                                    MVAHRAR--RR------------KRASATQLYQTCKQAGTCPSDVINKIEHTTLADKILQWGSLFTFFGNLGIGTGAGSGGRAGYVPL--G-TR--PTTVVDAS-PARPPIVV-----ESVGPTDPSIVTLVEESSVVNAGASFPN---F 122
HPVcand85_L2aa                             MVSHRAA--RR------------KRASATDLYKTCKQSGTCPPDVINKVEGTTLADKLLQWTSLGIFLGGLGIGTGSGTGGRTGYIPL--G-GR--PNTVVDVS-PARPPVVI-----ESVGPSDPSIVTLVEESSIVTSGAPVPT---F 122
HPV39_L2aa                                 MVSHRAA--RR------------KRASATDLYRTCKQSGTCPPDVVDKVEGTTLADKILQWTSLGIFLGGLGIGTGTGTGGRTGYIPL--G-GR--PNTVVDVS-PARPPVVI-----EPVGPSEPSIVQLVEDSSVITSGTPVPT---F 122
HPV70_L2aa                                 MVSSRAS--RR------------KRASATDIYKTCKQSGTCPPDVVNKVEGTTLADRFLQWASLGIFLGGLGIGTGTGTGGRTGYIPL--G-GR--PSTVVDVT-PARPPVVI-----EPVGPTEPSIVQLVEESSVVSSGTPIPT---F 122
HPV59_L2aa                                 MVSHRAA--RR------------KRASATDLYKTCKQAGTCPSDVINKVEGTTLADKILQWTSLGIFLGGLGIGTGSGTGGRTGYIPL--G-GR--TNTIVDVS-PAKPPVVI-----EPVGPTDPSIVTLVEDSSVITSGAPAPT---F 122
HPV18_NP_040316.1                          MVSHRAA--RR------------KRASVTDLYKTCKQSGTCPPDVVPKVEGTTLADKILQWSSLGIFLGGLGIGTGSGTGGRTGYIPL--G-GR--SNTVVDVG-PTRPPVVI-----EPVGPTDPSIVTLIEDSSVVTSGAPRPT---F 122
HPV18_CAA28670.1                           MVSHRAA--RR------------KRASVTDLYKTCKQSGTCPPDVVPKVEGTTLADKILQWSSLGIFLGGLGIGTGSGTGGRTGYIPL--G-GR--SNTVVDVG-PTRPPVVI-----EPVGPTDPSIVTLIEDSSVVTSGAPRPT---F 122
HPV45_L2aa                                 MVSHRAA--RR------------KRASATDLYRTCKQSGTCPPDVINKVEGTTLADKILQWSSLGIFLGGLGIGTGSGSGGRTGYVPL--G-GR--SNTVVDVG-PTRPPVVI-----EPVGPTDPSIVTLVEDSSVVASGAPVPT---F 122
[HPV82]                                    MVAARAR--RR------------KRASVTQLYSTCKAAGTCPPDVIPKVKGTTLADKILQWSGLGIFLGGLGIGTGSGTGGRTGYIPLGGG-GR--PG-VVDIA-PARPPIII-----EPVAPTEPSIVNLVEDSSIINSGSTIPT---F 123
[HPV69]                                    MVAVRAS--RR------------KRASATDLYKTCKAAGTCPPDVIPKIEGSTLADKILQWSGLGIFLGGLGIGTGTGTGGRTGYIPLGGG-GR--PS-VVDIG-PTRPPIII-----EPVGPTEPSIVTLVEESSIIQSGSPFPN---F 123
[HPV26]                                    MVAVRAP--RR------------KRASATDLYKTCKAAGTCPPDVIPKIEGSTLADKILQWSGLGIFLGGLGIGTGTGSGGRTGYIPLGGG-GR--PS-VVDIG-PTRPPIII-----EPVGPTEPSIVTLVEESSIIQSGAPIPT---F 123
[HPV26b_CAA52534.1]                        MVAVRAP--RR------------KRASATDLYKTCKAAGTCPPDVIPKIEGSTLADKILQWSGLGIFLGGLGIGTGTGSGGRTGYIPLGGG-GR--PS-VVDIG-PTRPPIII-----EPVGPTEPSIVTLVEESSIIQSGAPIPT---F 123
Pcpv1_L2aa                                 MAHSRPR--RR------------KRASATQLYQTCKASGTCPPDIIAKVEQNTLADKILKWGSLGVFFGGLGIGTGSGTGGRTGYVPV--Q-TA--PRPAIPFGPTARPPIIV-----DTVGPSDSSIVSLVEDSTIINSAASDFV---P 123
[ccpv1]                                    MAHSRPR--RR------------KRASATQLYQTCKASGTCP-DIIPKVEQNTLADKILKWGSLGVFFGGLGIGTGSGTGGRTGYVPL--E-SA--PRPAIPFGPTARPPIVV-----DTVGPTDSSIVSLVEDSAIINSGASDLV---P 122
[HPV6b]gil9626061lreflNP_040303.1l         MAHSRAR--RR------------KRASATQLYQTCKLTGTCPPDVIPKVEHNTIADQILKWGSLGVFFGGLGIGTGSGTGGRTGYVPL--Q-TS--AKPSITSGPMARPPVVV-----EPVAPSDPSIVSLIEESAIINAGAPEIV---P 123
[HPV74]gil27462490lgblAAO15461.1lAF436130_7 MAHSRAR--RR------------KRASATQLYQTCKVSGTCPSDIIPKIEHNTIADQILKWGSLGVFFGGLGIGTGSGTGGRTGYIPLQSA-PR--PDI--PSGPAARPPIIV-----DTVGPGDPSIVSLVEESAIINSGAPEVV---P 123
HPV7gil9627394lreflNP_041858.1l            MVSSRPR--RR------------KRASATQLYQTCKAAGTCPPDVVNKVEQTTVADQILKWGSMGVFFGGLGIGSGSGSGGRAGYVPLSTG-SRAIPPKSLAPDVIARPPVVV-----DTVAPTDPSIVSLIEESSIIQSGAPSPV---I 127
Hpv7gi|397066|emb|CAA52480.1|              MVSSRPR--RR------------KRASATQLYQTCKAAGTCPPDVVNKVEQTTVADQILKWGSMGVFFGGLGIGSGSGSGGRAGYVPLSTG-SRAIPPKSLAPDVIARPPVVV-----DTVAPTDPSIVSLIEESSIIQSGAPSPV---I 127
[HPV40]                                    MVSSRPR--RR------------KRASATQLYQTCKAAGTCPPDVVHKVEQTTVADQILKWGSMGVFFGGLGIGSGSGTGGRAGYVPLSTG-SRAVPPKSLVPDVVARPPVVV-----DTVAPSDPSIVSLIEESSIIQSGAPSLT---I 127
[HPV43]                                    MVSHTHK--RR------------KRASATQLYQTCKAAGTCPSDVINKVEHTTIADQILKWASMGVYFGGLGIGTGSGTGGRTGYVPLTTGRTGIVPKVTAEPGVVSRPPIVV-----ESVAPTDPSIVSLIEESSIIQSGAPITN---I 128
[Hpv91]                                    MVSYTLK--RR------------KRASATQLYQTCKAAGTCPSDVINKVEHTTIADQILKWASMGVYFGGLGIGTGSGSGGRTGYVPIPTGRTGTVPKVSAEPGVVSRPPVVI-----EPVAPTDPSIVSLIEESSIIESGAPITT---I 128
[HPV54]                                    MAKARAP--RR------------KRASATQLYQTCKASGTCPSDVIPKVEGTTIADQILRWGSMGVFFGGLGIGTGSGTGGRTGYIPL--G--R--PSTTLEPGPPVRPAGAV-----ETVAPSDPSIVSLVEESSVVDVGAPTPT---I 122
[HPV32]gi|9627332|ref|NP_041805.1|         MPPHRSR--RR------------KRASATQLYQTCKASGTCPPDVIPKIEGRTWADQILKWGSTGVFFGGLGIGTGAGSGGRTGYVPI--G-TR--PPVVAEPGPAIRPPVVV-----DTIGPTDPSVISLLEESAVIDSSIPVPT---D 123
[HPV32]gi|396987|emb|CAA52553.1|           MPPHRSR--RR------------KRASATQLYQTCKASGTCPPDVIPKIEGRTWADQILKWGSTGVFFGGLGIGTGAGSGGRTGYVPI--G-TR--PPVVAEPGPAIRPPVVV-----DTIGPTDPSVISLLEESAVIDSSIPVPT---D 123
[HPV4]2gi|333218|gb|AAA47047.1|           MPPQRSR--RR------------KRASATQLYQTCKASGTCPPDVIPKVEGTTLADKILQWGSLGVFFGGLGIGTGAGTGGRTGYVPL--G-TR--PPVIAEPGPAVRPPIAV-----DTVGPSDPSIVSLLEESSVIDAGITVPD---I 123
HPV34_L2aa                                 MRRKRDTHIRR------------KRASATQLYKTCKQSGTCPPDIIPKVEGNTLADQILKYGSIGVFFGGLGIGSGSGTGGRTGYVPLPTT-TP--SRPVEIPLQPTRPPVIT------SVGASDSSIVSLVEESSFIEAGVPGPT-SIV 128
HPV34gi|9627339|ref|NP_041811.1|           MRRKRDTHIRR------------KRASATQLYKTCKQSGTCPPDIIPKVEGNTLADQILKYGSIGVFFGGLGIGSGSGTGGRTGYVPLPTT-TP--SRPVEIPLQPTRPPVIT------SVGASDSSIVSLVEESSFIEAGVPGPT-SIV 128
HPV34gi|396995|emb|CAA52559.1|             MRRKRDTHIRR------------KRASATQLYKTCKQSGTCPPDIIPKVEGNTLADQILKYGSIGVFFGGLGIGSGSGTGGRTGYVPLPTT-TP--SRPVEIPLQPTRPPVIT------SVGASDSSIVSLVEESSFIEAGVPGPT-SIV 128
[HPV73]                                    MRRKRDTHIRK------------KRASATQLYKTCKQAGTCPPDVIPKVEGSTIADNILKYGSIGVFFGGLGIGSGSGSGGRTGYVPLSTG-TP--SKPVEIPLQPIRPSVVT------SVGPSDSSIVSLVEESSFIESGIPGPT-SIV 128
RMPV_L2aa                                  MKHAHLS--RRKRAAPRPPGGRQKRASATQLYQTCKAAGTCPPDVIPKVEGTTVADQILKYGSMGVYFGGLGIGSGAGTGGRSGYVPL--G-SR--PASIPEPLP--RPPVTI-----EPVGPSDPSIVSLLEESRLIEAGVPAPT---F 133
HPV52_L2aa                                 MRYRRST--RH------------KRASATQLYQTCKASGTCPPDVIPKVEGTTIADQLLKYGSLGVFFGGLGIGTGAGSGGRAGYVPL--S-TR--PPTSSITTSTIRPPVTV-----EPIGPLEPSIVSMIEETTFIESGAPAPS---I 123
HPV67_L2aa                                 MRHKRST--RR------------KRASATQLYQTCKAAGTCPPDVIPKVERTTIADQILKFGSLGVFFGGLGIGTGSGTGGRTGYVPL--S-TR--PPTASAPTSTIRPPVSV-----DTVGPLDSSIVSMIEETSFIESGAPAPS---I 123
HPV33_L2aa                                 MRHKRST--RR------------KRASATQLYQTCKATGTCPPDVIPKVEGSTIADQILKYGSLGVFFGGLGIGTGSGSGGRTGYVPI--G-TD--PPTAAIPLQPIRPPVTV-----DTVGPLDSSIVSLIEETSFIEAGAPAPS---I 123
HPV58_L2aa                                 MRHKRST--RR------------KRASATQLYQTCKASGTCPPDVIPKVEGTTIADQILRYGSLGVFFGGLGIGTGSGTGGRTGYVPL--G-ST--PPSEAIPLQPIRPPVTV-----DTVGPLDSSIVSLIEESSFIDAGAPAPS---I 123
[HPV35]                                    MRHKRST--KR-----------VKRASATQLYRTCKAAGTCPPDVIPKVEGNTVADQILKYGSMAVFFGGLGIGSGSGTGGRSGYVPL--G-TT--PPTAA-TNIPIRPPVTVESIPLDTIGPLDSSIVSLVEETSFIESGAPVVTPRVP 131
HPV31_L2aa                                 MRSKRST--KR-----------TKRASATQLYQTCKAAGTCPSDVIPKIEHTTIADQILRYGSMGVFFGGLGIGSGSGTGGRTGYVPL--S-TR--PSTVSEASIPIRPPVSI-----DPVGPLDPSIVSLVEESGIVDVGAPAPIPH-P 126
[HPV16EAvar]                               MRHKRSA--KR-----------TKRASATQLYKTCKQAGTCPPDIIPKVEGKTIADQILQYGSMGVFFGGLGIGTGSGTGGRTGYIPL--G-TR--PPTATDTLAPVRPPLTV-----DPVGPSDPSIVSFVEETSFIDAGAPTSVPSIP 127
HPV16_L2aa                                 MRHKRSA--KR-----------TKRASATQLYKTCKQAGTCPPDIIPKVEGKTIAEQILQYGSMGVFFGGLGIGTGSGTGGRTGYIPL--G-TR--PPTATDTLAPVRPPLTV-----DPVGPSDPSIVSLVEETSFIDAGAPTSVPSIP 127
[Hpv16NP_041331.1]                         MRHKRSA--KR-----------TKRASATQLYKTCKQAGTCPPDIIPKVEGKTIAEQILQYGSMGVFFGGLGIGTGSGTGGRTGYIPL--G-TR--PPTATDTLAPVRPPLTV-----DPVGPSDPSIVSLVEETSFIDAGAPTSVPSIP 127
[HPV16EGvar]                               MRHKRSA--KR-----------TKRASATQLYKTCKQAGTCPPDIIPKVEGKTIADQILQYGSMGVFFGGLGIGTGSGTGGRTGYIPL--G-TR--PPTATDTLAPVRPPLTV-----DPVGPSDPSIVSLVEETSFIDAGAPTSVPSIP 127
[HPV16AAvar]                               MRHKRSA--KR-----------TKRASATQLYKTCKQAGTCPPDIIPKVEGKTIADQILQYGSMGVFFGGLGIGTGSGTGGRTGYIPL--G-TR--PPTATDTLAPVRPPLTV-----DPVGPSDPSIVSLVEETSFIDAGAPTPVPSIP 127
[HPV16Af2]                                 MRHKRSA--KR-----------TKRASATQLYKTCKQAGTCPPDIIPKVEGKTIADQILQYGSMGVFFGGLGIGTGSGTGGRTGYIPL--G-TR--PPTATDTLAPVRPPLTV-----DPVGPSDPSIVSLVEETSFIDAGAPTPVPSIP 127
[HPV16Af1]                                 MRHKRSA--KR-----------TKRASATQLYKTCKQAGTCPPDIIPKVEGKTIADQILQYGSMGVFFGGLGIGTGSGTGGRTGYIPL--G-TR--PPTATDTLAPVRPPLTV-----DPVGPSDPSIVSLVEETSFIDVGAPTPVPSIP 127
                                           1.......10........20........30........40........50........60........70........80........90.......100.......110.......120.......130.......140.......150
```

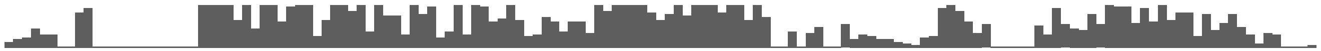

CLUSTAL 2.0.5 MULTIPLE SEQUENCE ALIGNMENT


```
                                         .   ** : ::       .**::::           : :   ** : :*          .  * .         :      *:**: .*              *** *          :  ** :    *  :     *:  *  : .  ** ::      : :
[HPVcand89]                              TGTDGFEITTSS---TTTPAVLDITPSGATVQVSSTSYSNPAFTEPSIIEPPQHGSVSGHVFTS--TPTSGSHTFEEIPMSTFATSGDVDAGPISSTPVPGVRRIAGPRLNLYSKATQQVPVSDSAFVSRPASFVTYDNPVYDP---EET   264
[HPV81]                                  TGTSGFEITTSS---TTTPAVLDITPTGTTVQVSSTNFLNPLYTEPSIIDPPQTGEITGHVFTS--TPTAGSHSYEEIPMVTFASNTGTGGEPISSTPMPGVRRLAGPRLRLYTKATQQVPVRDPAFVSHPASFATFDNPVYDP---EET   264
[HPV61]                                  SGSGGFNVTSSS---TTTPAVLDITPSGGSVQVSSTSFINPLFTEPSIIEPPQAGDLAGHVISS--TPTAGSHSFEEIPMHTFATS----EGPGSSTPLPGIRRLARPRLNLYSKANQQIKVANPTFMSDPASLITYDNPIFDP---EET   258
[HPV72]                                  TGSGGFEVTTSS---TTTPAVLDITPSGASVQVSSSSFTNPLFTEPSIIEPPQAGDLSGHVFTS--TPTSGSHSFEEIPMHTFATHSSTSTDPFSSTPLPGVRRLAQPRLGLYSKANQQVRVTNPAFLSRPQSLVTYDNPVYDP---EET   264
[HPV83]                                  TGTHGFEVTSSS---TTTPAVLDITPSSATVQVSSSSFTNPLFTEPSIIEPPQHGDITGHVFTS--TATSGTHGFEEIPMQTFATSGGTGQEPISSTPIPGVRRVAGPRLGLYSRATSQVKVPDSAFMSRPASFVTYDNPVFDP---DET   264
[HPV84]                                  TGTGGFEVTTSS---ITTPAVLDITPSGSSVQVSSTSYVNPLFTEPSIIEPPQAGDITGHVLSS--TATSGSHTYEEIPMQTFAVQGGTGLEPISSTPTPGVRRLAGPRLNLYSRATQQVPVADTAFLSRPESFVTFDNPVFDP---EET   264
[HPV86]                                  TGTGGFEITTSS---TTTPAVLDITPSGSSVQVSSTSYINPLFTEPSIIEVPQAGDITGHVLSS--TATSGTHTYEEIPMQTFAVQGGTGHEPISSTPIPGVRRLAGPRLNLYSRATQQVPVADSAFLSRPDSFVTFDNPVYDP---DET   264
[HPVcand87]                              TGTGGFEVTTSS---TTTPAVLDITPSGSSVQVSSTSYINPLFTEPSIIEVPQAGDIAGHVLSG--TATSGTHTFEEIPMQTFAVQGGTGQEPISSTPIPGVRRLAGPRLNLYSRVTQQVPVTDSAFLSRPESFVTFHNPMYDP---EET   262
[HPV71]                                  TGTGGFEVTTAS---TTTPAVLDITP-GNTVQVSSSSFTNPSFTEPALVEPPQTGEVSGHILVS--TSTSGTHGYEEIPMQTFASE-GTGNEPISSTPIPGVRRLAGPR--LYSRAYQQVRVDESTFLRHPASMVTYDNPVYDP---EET   259
[HPV90]                                  TGTGGFEVTTAS---TTTPAVLDITPSGGNVQVSSSSFINPLFTEPAIVEPPQAGEVTGHVLVS--TPTAGSHGYEEIPMQTFAVR-GTGDEPIISTPLPGVRRVAGPR--LYSRAYQQVHVQDPRFLSQPETLVTYDNPVFDT---EET   260
[HPV10]gil9627262lreflNP_041745.1l       SGTSGFEVTSSA---TTTPAVLDITPASENVVISSTNFTNPAFTEPSLVEVPQSGEVSGHILIS--TPTAGTHGYEEIPMDTFASS-GTGTEPISSTPVPGVSRIAGPR--LYSRANTQVKVSDPAFLSRPSSLLTFDNPVFEPE--DET   263
[Hpv10]gil396907lemblCAA52493.1l         SGTSGFEVTSSA---TTTPAVLDITPASENVVISSTNFTNPAFTEPSLVEVPQSGEVSGHILIS--TPTAGTHGYEEIPMDTFASS-GTGTEPISSTPVPGVSRIAGPR--LYSRANTQVKVSDPAFLSRPSSLLTFDNPVFEPE--DET   263
[Hpv94]                                  SGTGGFEVTSSA---TTTPAVLDITPAGDNVVISSTSFTNPAYTEPSLVEVPQSGEVSGHILIS--TPTAGTHGYEEIPMDTFASS-GTGTEPISSTPVPGVSRIAGPR--LYGKATTQVKVSDPAFLSRPSTFLTFDNPAFEPE--DET   263
[Hpv3]                                   TGTDGFEVISSA---TTTPAVLDITPASDNVVVSSTNFSNPAFTEPSLLEVPQNGEVSGHILIS--TPTSGTHGYEEIPMETFASP-GTGTEPISSTPVPGVSRIAGPR--LYSKAVTQVKVTDPAFLTRPRSLMTFDNPVFEPE--DET   263
hpv28                                    SGTGGFEVTSSA---TTTPAVLDITPATDNVVISSSNFTNPAFTEPSLLEVPQNGEVSGHILVS--TPTAGTHSYEEIPMETFASP-GTGNEPISSTPVPGVSRIAGPR--LYAKAVTQVKVTDPAFLSRPTSLVTFDNPAFEPG--DET   263
hpv29                                    TGTSGFEITSSA---TTTPAVLDITPAGDNVVITSTNFNNPLFTEPSLLEIPQTGETSGRVLVG--TPTSGVHGYEEIPMDTFATS-GTGLEPISSTPVPGVSRVAGPR--LYGKALTQVRVSDPAFLTQPSSFVTFDNPVYDPE--DET   263
[Hpv77]                                  TGTSGFEITSSA---TTTPAVLDITPAGDTVVVTSTNFTNPLYTEPSLVEVPQTGEISGHLLVS--TATSGTHGYEEIPMDTFATS-GTGSEPISSTPVPGVSRVAGPR--LYGKAMTQVRVPDPAFLSRPSSFVTFDNPVYDPG--DET   263
[HPV56]                                  TGSGGFEITSSS---TTTPAVLDITPTSSTVHVSSTHITNPLFIDPPVIEAPQTGEVSGNILIS--TPTSGIHSYEEIPMQTFAVH-GSGTEPISSTPIPGFRRIAAPR--LYRKAFQQVKVTDPAFLDRPATLVSADNPLFEGT--DTS   262
[hpv66]gi|1020296|gb|AAA79504.1|         TGSGGFEVTSSS---TTTPAVLDITPTSSTVHVSSTTITNPLYIDPPVIEAPQTGEVSGNILIS--TPTSGIHSYEEIPMQTFAIH-GTGNEPISSTPIPGFRRLAAPR--LYSRAFQQVRVTDPAFLDNPTTLISADNPVFEGA--DTT   262
[HPV53]gi|9627381|ref|NP_041847.1|       TGTAGFEVTSSS---TTTPAVLDITPTSTSVHVSSTTYTNPTFVDPPVIEVPQTGEVSGNILIS--TPTSGVHSYEEIPMQTFAVQ-GTGNEPISSTPIPGLRRIAAPR--LYKKAFQQVKVTDPAFLHKPETLINVDNPIFQNA--DTT   262
[HPV53]gi|397051|emb|CAA52594.1|         TGTAGFEVTSSS---TTTPAVLDITPTSTSVHVSSTTYTNPTFVDPPVIEVPQTGEVSGNILIS--TPTSGVHSYEEIPMQTFAVQ-GTGNEPISSTPIPGLRRIAAPR--LYKKAFQQVKVTDPAFLHKPETLINVDNPIFQNA--DTT   262
[HPV30]                                  TGTAGFEVTSSS---TTTPAVLDITPTTGSVHVSSTHFTNPSFVEPPVIEVPQTGEVSGHILVS--TPTSGVHSYEEIPMQTFAVH-GTGTEPISSTPIPGLRRIAAPR--LYQRAFQQVKVTDPTFLTKPETLITVDNPVFEDA--DTT   262
HPVcand85_L2aa                           TGTSGFEITSSA---TTTPAVLDITPASGSVQLSSTSFTNPAFTDPSVIEVPQTGEVSGDIFIT--TPTSGTHGYEEIPMHTFATQ-GRGTEPISSTPIPGVRRVAGPR--LYSQAYQQVKITNSDFISRPSTLVTFTNPAYEPI--DTT   262
HPV39_L2aa                               TGTSGFEITSSS---TTTPAVLDITPSSGSVQITSTSYTNPAFTDPSLIEVPQTGETSGNIFVS--TPTSGTHGYEEIPMEVFATH-GTGTEPISSTPTPGISRVAGPR--LYSRAHQQVRVSNFDFVTHPSSFVTFDNPAFEPV--DTT   262
HPV70_L2aa                               TGTSGFEITSSA---TTTPAVLDITPASGSVQISTTSYTNPAFADPSLIEVPQTGEVSGNIFVT--TPTSGTHGYEEIPMQVFASH-GTGTEPISSTPVPGVSRVAGPR--LYSRAYHQVRVNNFDFVTRPSSFVTFDNPAFEPG--DTS   262
HPV59_L2aa                               TGTSGFEISTSS---TTTPAVLDITPTS-SVQISSSSFINPAFTDPSVIEVPQTGEISGNILIS--TPTSGAHGYEEIPMQTFATE-GTGLEPISSTPNPTVRRVAGPR--LYSRANQQVRVSNADFLTRPSTFVTYDNPAYDPI--DTT   261
HPV18_NP_040316.1                        TGTSGFDITSAG---TTTPAVLDITPSSTSVSISTTNFTNPAFSDPSIIEVPQTGEVAGNVFVG--TPTSGTHGYEEIPLQTFASS-GTGEEPISSTPLPTVRRVAGPR--LYSRAYQQVSVANPEFLTRPSSLITYDNPAFEPV--DTT   262
HPV18_CAA28670.1                         TGTSGFDITSAG---TTTPAVLDITPSSTSVSISTTNFTNPAFSDPSIIEVPQTGEVAGNVFVG--TPTSGTHGYEEIPLQTFASS-GTGEEPISSTPLPTVRRVAGPR--LYSRAYQQVSVANPEFLTRPSSLITYDNPAFEPV--DTT   262
HPV45_L2aa                               TGTSGFEITSSG---TTTPAVLDITPTVDSVSISSTSFTNPAFSDPSIIEVPQTGEVSGNIFVG--TPTSGSHGYEEIPLQTFASS-GSGTEPISSTPLPTVRRVRGPR--LYSRANQQVRVSTSQFLTHPSSLVTFDNPAYEPL--DTT   262
[HPV82]                                  TGTDGFEITSSS---TTTPAVLDITPSAGTVHVTSTNIENPLYIEPPFIEAPQSGEVSGHIFTS--TPTSGTHGYEEIPMEVFASNVTTGKEPISSTPTPGVRRIAAPR--LYSRAFSQVKVTNPDFISKPSTFVTFDNPAFEPA--DTS   264
[HPV69]                                  SGGDGFEVTTSS---TTTPAVLDITPSPGTVHVTSTNIQNPLYIEPP-VDIPQSGEALGHIFTS--TSTAGTHSYEEIPMEVFASNTSSGSKPISSTPIPGIRRVAAPR--LYSKAYQQVKVTDPNFISKPSTFITFDNPAYEPM--DTT   263
[HPV26]                                  SGGNGFELTTSS---ATTPAVLDITPSAGTVHVTSTNIQNPLYIEPP-IDIPQAGEASGHIFTT--TSTAGTHSYEEIPMEVFASTNGTGLEPISSTPIPGIQRVSAPR--LYSKAYQQVKVTDPNFIGNPSTFVTFDNPAYEPI--DET   263
[HPV26b_CAA52534.1]                      SGGNGFELTTSS---ATTPAVLDITPSAGTVHVTSTNIQNPLYIEPP-IDIPQAGEASGHIFTT--TSTAGTHSYEEIPMEVFASTNGTGLEPISSTPIPGIQRVSAPR--LYSKAYQQVKVTDPNFIGNPSTFVTFDNPAYEPI--DET   263
Pcpv1_L2aa                               PIREGFEISTSE---TTTPAILDVSVTTHNT-TSTSIFKNPAFAEPSIVQSQPSVEASGHVLTSTYTSTISSHSVEDIPLDTFIVS-SSDSNPASSTPVP--TPVARPRLGLYSKALQQVQVTNPAFLSSPQRLITFDNPAYEGE--DIS   264
[ccpv1]                                  SIHGGFEISTSE---STTPAILDVSITTHNT-TSTSIFRNPAFAEPSIVQSQPSVEAGGHLLTSTFTSTISPHSVEEIPLDTFIVS-SSNSNPASSTPVPTT--VARPRLGLYSKALHQVQVTDPAFLSSPQRLITFDNPVYEGE--DIS   263
[HPV6b]gil9626061lreflNP_040303.1l       PAHGGFTITSSE---TTTPAILDVSVTS---HTTTSIFRNPVFTEPSVTQPQPPVEANGHILIS--APTVTSHPIEEIPLDTFVVS-SSDSGPTSSTPVPGTAP--RPRVGLYSRALHQVQVTDPAFLSTPQRLITYDNPVYEGE--DVS   260
[HPV74]gil27462490lgblAAO15461.1lAF436130_7 PSHGGFEITTSE---STTPAILDVSVTTHNT-TSTSVFRNPSFTDPSVIQSQPPVEAGGHVLIS--SSTVSSHPVEEIPLDTFIVS-SSDSNPASSTPIPTAA--TRPRIGLYSRALHQVQVTDPAFLSSPQRLITFDNPVYEGE--DVS   262
HPV7gil9627394lreflNP_041858.1l          PTEGGFSITSSG---TDVPAILDIS-STNTVHVTSTTHHNPIFTDPSVVQPIPPVEASGRIIVS--HSSITTGAAEEIPMDTFVVH----SDPLSSTPVPGVS--ARPKVGLYSKALQQVEIVDPTFMSTPQRLITYDNPVFDNI--EDT   263
Hpv7gi|397066|emb|CAA52480.1|            PTEGGFSITSSG---TDVPAILDIS-STNTVHVTSTTHHNPIFTDPSVVQPIPPVEASGRIIVS--HSSITTGAAEEIPMDTFVVH----SDPLSSTPVPGVS--ARPKVGLYSKALQQVEIVDPTFMSTPQRLITYDNPVFDNI--EDT   263
[HPV40]                                  PTEGGFSVTSSG---TDVPAILDVS-STNTVHVTATTHHNPVFTDPSVVQPIPPVEAGGRLIVS--HSTITTSAAEEIPLDTFVVH----SDPLSSTPVPGTS--GRPRLGLYSKALQQVEIVDPAFLSTPQRLITYDNPVFENV--DDT   263
[HPV43]                                  PSHGGFEVTSSG---SEVPAILDVSPST-SVHITTSTHLNPAFTDPTIVQPTPPVEAGGRIIIS--HSTVTADSAEQIPMDTFVIH----SDPTTSTPIPGTAP--RPRLGLYSKALQQVEIVDPTFLSSPQRLITYDNPVFEDP--NAT   264
[Hpv91]                                  PSHGGFEVTSSG---SDVPAILDVSPTT-SVHVTTTTHLNPAFTDPAIVQPTPPLEAGGRIIIS--QSTITAHSAEDIPMDTFVVH----SDPLTSTPIPGNA--SRPRLGLYSKALQQIEIVDPAFLSSPQRLITYDNPVFEDP--DAT   264
[HPV54]                                  PSQGGFEIATSS---DATPAILDVTSTTTPIRVSITSHDNPIYTEPSLLDPPPPVQMDGRVLVS--TSTLQSSTAENIPMDTFIIM-QDHIGTTTSTPIP--RPPARPRLGLYSRALQQVPVQDPAFLQQPSSLITYDNPVYEGN-PDVT   263
[HPV32]gi|9627332|ref|NP_041805.1|       TSHGGFNITSSASGPSSTPAVLDISPPTNTIRVASTTSHNPVYSDPFTLRPSLPVEGNGRLLTS--HPTIAPHSYEEIPMDTFVVS-TDTSNTVTSTPIPGPRPTM--RLGLYTRVTQQRPVATTTFLTSPERLVTYDNPAYEGP-AEGT   267
[HPV32]gi|396987|emb|CAA52553.1|         TSHGGFNITSSASGPSSTPAVLDISPPTNTIRVASTTSHNPVYSDPFTLRPSLPVEGNGRLLTS--HPTIAPHSYEEIPMDTFVVS-TDTSNTVTSTPIPGPRPTM--RLGLYTRVTQQRPVATTTFLTSPERLVTYDNPAYEGP-AEGT   267
[HPV4]2gi|333218|gb|AAA47047.1|          TSHGGFNITTSTGGPASTPAILDISPPTNTIRVTTTTSTNPLYIDPFTLQPPLPAEVNGRLLIS--TPTITPHSYEEIPMDTFVVS-TDTTNTFTSTPIPGPRSSA--RLGLYSRATQQRPVTTSAFLTSPARLVTYDNPAYEGL-TEDT   267
HPV34_L2aa                               PSSSGFNVTTSV---DSTPAIIDVATISDTTQVSVSTFNNPTFTDPSVLQPPPPLEASGRLLFS--NDTVTTHSYENIPLDTFVVT-TDNNSIVSSTPIPGRHPPA--RLGLYGRAIQQVKVVDPAFVTTPTRLVTYDNPAFEGL-QDTT   269
HPV34gi|9627339|ref|NP_041811.1|         PSSSGFNVTTSV---DSTPAIIDVATISDTTQVSVSTFNNPTFTDPSVLQPPPPLEASGRLLFS--NDTVTTHSYENIPLDTFVVT-TDNNSIVSSTPIPGRHPPA--RLGLYGRAIQQVKVVDPAFVTTPTRLVTYDNPAFEGL-QDTT   269
HPV34gi|396995|emb|CAA52559.1|           PSSSGFNVTTSV---DSTPAIIDVATISDTTQVSVSTFNNPTFTDPSVLQPPPPLEASGRLLFS--NDTVTTHSYENIPLDTFVVT-TDNNSIVSSTPIPGRHPPA--RLGLYGRAIQQVKVVDPAFVTTPTRLVTYDNPAFEGL-QDTT   269
[HPV73]                                  PSTSGFDITTSV---NSTPAIIDVSAISDTTQISVTTFKNPTFTDPSVLQPPPPLEASGRLLFS--NDTVTTHSYENIPLDTFVVT-TDHNSIVSSTPIPGRQPAA--RLGLYGRAIQQVKVVDPAFLTTPTRLVTYDNPAFEGL-QDTT   269
RMPV_L2aa                                PTHGGFEISTSE---VSTPAVLDVSSGGSDVHVSVTSFTNPTFTEPSVLRPPPPVEASGRLVIS--ASSVSTHSYEEIPMDTFVIT-GDHNYNTTSTPIPGSRAPA--RLGLYGRATQQVRVVDPAFITTPARLVTYDNPAYEGV-DDAT   274
HPV52_L2aa                               PSATGFDVTTSA---NNTPAIINVTSIGESSVQSVSTHLNPTFTEPSIIQPPAPAEASGHVLFS--SPTISTHTYEEIPMDTFVTS-TDSSSVTSSTPIPGSRPTT--RLGLYSRATQQVKVVDPAFMSSPQKLVTYNNPVFEGVDTDET   265
HPV67_L2aa                               PTASGFDVATSA---DNTPAIINVSSIGESSVQSVTTHLNPTFTEPSVLRPFSSSEASGHLIFS--TPTISTHSYEDIPMDTFIVS-TTSDNVTSSTPIPRPRPTA--RLGLYSKGTQQVKVVDPAFLTSPRRLITFDNPAFQPTEPDET   265
HPV33_L2aa                               PTPSGFDVTTSA---DTTPAIINVSSVGESSIQTISTHLNPTFTEPSVLHPPAPAEASGHFIFS--SPTVSTQSYENIPMDTFVVS-TDSSNVTSSTPIPGSRPVA--RLGLYSRNTQQVKVVDPAFLTSPHKLITYDNPAFESFDPEDT   265
HPV58_L2aa                               PTPSGFDITTSA---DTTPAILNVSSIGESSIQTVSTHLNPSFTEPSVLRPPAPAEASGHLIFS--SPTVSTHSYENIPMDTFVIS-TDSGNVTSSTPIPGSRPVA--RLGLYSRNTQQVKVVDPAFLTSPHRLVTYDNPAFEGFNPEDT   265
[HPV35]                                  PTT-GFTITTST---DTTPAILDVT--------SISTHDNPTFTDPSVLHPPTPAETSGHFVLS--SSSISTHNYEEIPMDTFIVS-TDSNNITNSTPIPGSRPTT--RLGLYSKGTQQVKVVDPAFMTSPAKLITYDNPAYEGLNPDTT   264
HPV31_L2aa                               PTTSGFDIATTA---DTTPAILDVT--------SVSTHENPTFTDPSVLQPPTPAETSGHLLLS--SSSISTHNYEEIPMDTFIVS-TNNENITSSTPIPGVRRPA--RLGLYSKATQQVKVIDPTFLSAPKQLITYENPAYETVNAEES   260
[HPV16EAvar]                             PDVSGFSITTST---DTTPAILDI----NNTVTTVTTHNNPTFTDPSVLQPPTPAETGGHFTLS--SSTISTHNYEEIPMDTFIVS-TNPNTVTSSTPIPGSRPVA--RLGLYSRTTQQVKVVDPAFVTTPTKLITYDNPAYEGIDVDNT   265
HPV16_L2aa                               PDVSGFSITTST---DTTPAILDI----NNTVTTVTTHNNPTFTDPSVLQPPTPAETGGHFTLS--SSTISTHNYEEIPMDTFIVS-TNPNTVTSSTPIPGSRPVA--RLGLYSRTTQQVKVVDPAFVTTPTKLITYDNPAYEGIDVDNT   265
[Hpv16NP_041331.1]                       PDVSGFSITTST---DTTPAILDI----NNTVTTVTTHNNPTFTDPSVLQPPTPAETGGHFTLS--SSTISTHNYEEIPMDTFIVS-TNPNTVTSSTPIPGSRPVA--RLGLYSRTTQQVKVVDPAFVTTPTKLITYDNPAYEGIDVDNT   265
[HPV16EGvar]                             PDVSGFSITTST---DTTPAILDI----NNTVTTVTTHNNPTFTDPSVLQPPTPAETGGHFTLS--SSTISTHNYEEIPMDTFIVS-TNPNTVTSSTPIPGSRPVA--RLGLYSRTTQQVKVVDPAFVTTPTKLITYDNPAYEGIDVDNT   265
[HPV16AAvar]                             PDVSGFSITTST---DTTPAILDI----NNTVTTVTTHNNPTFTDPSVLQPPTPAETGGHFTLS--SSTISTHNYEEIPMDTFIVS-TNPNTVTSSTPIPGSRPVA--RLGLYSRTTQQVKVVDPAFVTAPTKLITYDNPAYEGIDVDNT   265
[HPV16Af2]                               PDVSGFSITTST---DTTPAILDI----NNTVTTVTTHNNPTFTDPSVLQPPTPAETGGHFTLS--SSTISTHNYEEIPMDTFIVS-TNPNTVTSSTPIPGSRPVA--RLGLYSRTTQQVKVVDPAFVTTPTKLITYDNPAYEGIDVDNT   265
[HPV16Af1]                               PDVSGFSITTST---DTTPAILDI----NNTVTTVTTHNNPTFTDPSVLQPPTPAETGGHFTLS--SSTISTHNYEEIPMDTFIVS-TNPNTVTSSTPIPGSRPVA--RLGLYSRTTQQVKVVDPAFVTTPTKLITYDNPAYEGIDVDNT   265
                                         .......160.......170.......180.......190.......200.......210.......220.......230.......240.......250.......260.......270.......280.......290.......300
```

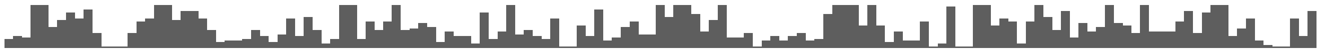

CLUSTAL 2.0.5 MULTIPLE SEQUENCE ALIGNMENT

File: /Users/johnlowe/Desktop/L2muscle.ps   Date: Fri May 2 13:22:30 2008


```
                                             . :          *:  *:::  **** :::::  :* **:* : :: ** *  :    ::: *:* *               *:..:                           :    *:
[HPVcand89]                                  IIFEHPDL--HAPPDPDFLDVVALHRPALTSRKGTVRFSRVGQRATLKTRSGKQIGARVHFYHDVSPI----SAA-----ESIEMQPL---LPS--------------PDLTQPVY----DIYADTTHL--DSVLAERPFPAYSSANTTF   380
[HPV81]                                      IIFEHPSI--YQPPDPDFLDIVQLHRPALTARQGTVRVSRIGQRASLRTRSGKHIGARVHFYHDISPI----PTE-----ADIELQPL---LPPSS------------VSSADALY----DVYADDQHL--DAVLQ----SVPSLSSRSS   378
[HPV61]                                      IIFEHPSI--YTPPDPDFLDIVSLHRPALTSRQGTVRFSRLGQRATLRTRSGRRIGARVHFYHDISPI-----PS-----DAVELQPL---VP---------------SSSPSITY----DIYADPEVL--DLPAQ--------HTQPTL   364
[HPV72]                                      IIFEHPSI--YTPPDPDFLDIISLHRPALTARQGTVRVSRLGQRATLRTRSGKRIGARVHFYQDISPI-----SS-----DTIEMQSL---AS---------------STQPDITY----DIYADPDLG--EPPPR------ASVSSTSL   372
[HPV83]                                      IIFEHPSL--HNPPDPDFLDIVSLHRPALTARQGTVRVSRVGQRATLRTRSGKQIGARVHFYHDISPI----PPT-----ESIELQPL---EP---------------SVANTSLY----DIYADDVFL--ESSVP--LRPSPSSTSVSL   377
[HPV84]                                      IIFEHPSL--HAPPDPDFLDIVTLHRPALTARRSGVRFSRIGQRASMRTRSGKHIGARVHFYHDLSPI----PHL-----EDIELQPL---VSSS-------------AVPTDSLY----DIYADDAHL--SSVLR---PPSVSALRPAS   378
[HPV86]                                      IIFEHPSL--HAPPDPDFLDIVTLHRPALTARRSGVRYSRIGQRASMRTRSGKHIGARVHFYHDLSPI----AQA-----EDIELQPL---GATTS---------SGQASGQDTLY----DIYADDTHL--SSILQ-----EPSVPSLHS   380
[HPVcand87]                                  IIFEHPSL--HAPPDPDFLDIVTLHRPALTARRSGVRYSRIGQRASMRTRSGKHIGARVHFYHDISPI----APA-----DSIELQPL---VSST-------------APSDDTLY----DIYADDTHL--SSVLR---GPSLPSLHSST   376
[HPV71]                                      IIFEHPSI--HQAPDPAFMDIVALHRPALTARKGTVRFSRLGQKSTLRTRSGKQIGARVHFYHDISPI----QPT-----EHLELQPLGRALQ---------------QEPIDTLY----DIYLTQIIP--MILSFNLLLCPAGLHLLLY   377
[HPV90]                                      ILFEHPSI--HQVPDPDFLDIVALHRPALTARRGTVRYSRLGQRATLKTRSGKRIGATVHFYQDLSPIA---PVA-----DELEMQPL---VSDT-------------PDYLDSLY----DIYADTASVSRHRTLT----PTRPSTPLQA   376
[HPV10]gil9627262lreflNP_041745.1l           IIFERPYSP-SRVPDPDFLDIVRLHRPALTSRRGTVRFSRLGQKFSMRTRSGKGIGARVHYYQDLSPI----API-----EDIEMEPL---LA---------------PAASDTIY----DIFADVDDG--DVAFT---EGYRSTTQSRG   376
[Hpv10]gil396907lemblCAA52493.1l             IIFERPYSP-SRVPDPDFLDIVRLHRPALTSRRGTVRFSRLGQKFSMRTRSGKGIGARVHYYQDLSPI----API-----EDIEMEPL---LA---------------PAASDTIY----DIFADVDDG--DVAFT---EGYRSTTQSRG   376
[Hpv94]                                      IIFERPYSP-SRVPDPDFLDIVRLHRPALTSRRGAVRFSRLGQKFSMRTRSGKNIGARVHYYHDLSPI----AAT-----EEIEMEPL---LA---------------PADPDTLY----DIFADVDDG--DVAFT---NGTRSVTPARS   376
[Hpv3]                                       IIFERPYSP-SQVPDSDFLDILRLHRPALTSRRGTVRYSRVGQKLSMRTRSGKGLGARVHYYQDLSPI----GPT-----EDIEMEPL---IAPAS------------ASAYDSLY----DVYADVDDA--DIGFT---SGGRSDTLSRG   379
hpv28                                        IIFERPYPP-SQVPDPDFMDIIRLHRPALTSRRGTVRFSRLGTKLSMHTRSGKGIGARVHYYQDLSPI----GPT-----EDIEMEPL---LAPAE------------NAAGDSIY----DVFADVEDA--DIAFT---GRSRSATSSRG   379
hpv29                                        IIFERPSPG-TRVPDPDFMDIVKLHRPALTSRRGTVRFSRVGQKFSMRTRSGTNIGARVHYYHDLSPI----LPT-----EDIELEPL---LPPAD------------PTAEESLY----DIYADVDEA--DMAFT---GGGRGATTYGG   379
[Hpv77]                                      IIFERPSPG-TRVPDPDFLDIVRLHRPALTSRRGTVRFSRVGQKFSMRTRSGTQIGARVHYYHDLSPI----THT-----EDIELEPL---LPPAD------------SAAEDSLY----DVYADVDDA--DVAFT---NSGRNLTSFGG   379
[HPV56]                                      LAF-SPS---GVAPDPDFMNIVALHRPAFTTRRGGVRFSRLGRKATIQTRRGTQIGARVHYYYDISPI----AQA-----EEIEMQPL---LSA--------------NNSFDGLY----DIYANIDDE--APGLSSQSVATPSAHLPIK   376
[hpv66]gi|1020296|gb|AAA79504.1|             LTF-SPS---GVAPDPDFMDIVALHRPAFTTRRTGVRFSRLGKKATMQTRRGTQIGARVHYYYDISPI----AQA-----DEIEMQPL---LST--------------DNSFDGLY----DIYANIDDE--APISFRQSGATPSAQLPIK   376
[HPV53]gi|9627381|ref|NP_041847.1|           LTF-SPS---GVAPDPDFLDIVALHRPAFTTRRGGVRFSRLGTKATMRTRSGKQIGARVHYYYDVSPI----TQT-----EEIEMQPL---LST--------------DNTFDGLY----DIYANIDDE--APVSS----RFSIATPSRL   372
[HPV53]gi|397051|emb|CAA52594.1|             LTF-SPS---GVAPDPDFLDIVALHRPAFTTRRGGVRFSRLGTKATMRTRSGKQIGARVHYYYDVSPI----TQT-----EEIEMQPL---LST--------------DNTFDGLY----DIYANIDDE--APVSS----RFSIATPSRL   372
[HPV30]                                      LTF-SPS---GVAPDPDFLDIVALHRPAFTTRRGGVRFSRLGTKATMRTRSGKQIGARVHYYYDVSPI----AHT-----EEIEMQPL---LSA--------------NNSFDGLY----DIYANLDDE--APVSS----HLSIATPSRL   372
HPVcand85_L2aa                               LTF-SPQ---DVVPDPDFMDIVRLHRPALTSRRGTVRFSRLGKKLTMSTRSGKQIGAQVHYYHDISPIS---HIG-----ESIEMQPL---LPDAA-----------VTADTNGLF----DIYADTDID--NNAML----YDRNISDVTQ   376
HPV39_L2aa                                   LTYEAAD----IAPDPDFLDIVRLHRPALTSRKGTVRFSRLGKKATMVTRRGTQIGAQVHYYHDISSI----APA-----ESIELQPL---VHAEP------------SDASDALF----DIYADVDNN--TYLDT-----AFNNTRDSG   373
HPV70_L2aa                                   LTFEPAD----TAPDPDFLDIVRLHRPALTSRRGTVRFSRLGKKATMFTRRGTQIGAQVHYYHDISNI----TAT-----EDIEMQPL---LT---------------SESTDGLY----DIYADADID--NAMLH-----TTSHTGSTG   370
HPV59_L2aa                                   LTF-DPS---SEVPDPDFMDIVRLHRPALTSRRSTVRFSRLGQRATMFTRSGKQIGARVHFYHDISPI----PHA-----EDIELQPL---VSS--------------QAATDDIY----DIYADITDE--APTST----ANTAFTIPKS   371
HPV18_NP_040316.1                            LTF-DPR---SDVPDSDFMDIIRLHRPALTSRRGTVRFSRLGQRATMFTRSGTQIGARVHFYHDISPI----APSP----EYIELQPL---VS---------------ATEDNDLF----DIYADDMDP--AVPVP-----SRSTTSFAF   371
HPV18_CAA28670.1                             LTF-DPR---SDVPDSDFMDIIRLHRPALTSRRGTVRFSRLGQRATMFTRSGTQIGARVHFYHDISPI----APSP----EYIELQPL---VS---------------ATEDNDLF----DIYADDMDP--AVPVP-----SRSTTSFAF   371
HPV45_L2aa                                   LSF-EPT---SNVPDSDFMDIIRLHRPALSSRRGTVRFSRLGQRATMFTRSGKQIGGRVHFYHDISPI----AAT-----EEIELQPL---IS---------------ATNDSDLF----DVYADFPPP--ASTTP-----STIHKSFTY   370
[HPV82]                                      LSFEEPT---DVAPDPDFLDIIKLHRPALTSRRGTVRFSRLGQKATIRTRSGKQIGARGHYYHDISSI----APA-----EELEMQPL---LS---------------PSTNNYSY----DIYADLNEA--ETGFM---QPTQTTPMLRS   375
[HPV69]                                      LTFSADS---HVAPDPDFLDIIALHRPALTSRRGTVRFSRLGQKATLKTRSGKQIGAKVHYYHDISPIH---ATE-----EAIELQPL---ITS--------------EQHSTPLF----DVYADADPA---PTFT-----FPSTTPTTI   373
[HPV26]                                      LTYASSS---TVAPDPDFLDIIALHRPALTSRKGTVRYSRLGQKATMKTRSGKQIGATVHYYHDISPIQSF-AEH-----EEIELQPL---HTS--------------THSSAPLF----DIYADPDTV--PSIHT--PRMSYSPTTLPV   379
[HPV26b_CAA52534.1]                          LTYASSS---TVAPDPDFLDI-ALHRPALTSRKGTVRYSRLGQKATMKTRSGKQIGATVHYYHDISPIQSF-AEH-----EEIELQPL---HTS--------------THSSAPLF----DIYADPDTV--PSIHT--PRMSYSPTTLPV   378
Pcpv1_L2aa                                   LQFQHNTI--HNPPDDAFMDIVRLHRPAITSRRGIVRFSRIGQRGSMYTRSGKHIGGRVHFYTDISPIS---AAA-----EELEMQPL---VAA--------------AQDDSGLF----DVYVDPTPG--PVAVQ----------NMSY   371
[ccpv1]                                      LHFEHNSI--HEPPNEAFMDIIRLHRPAITSRRGVVRFSRIGQRGSMYTRSGKHIGGRVHFFTDISPIS---ADA-----QDIELQPL---VAA--------------AQDDSDLF----DIYVDPDTT--PVAVD---------NIPSA   371
[HPV6b]gil9626061lreflNP_040303.1l           VQFSHDSI--HNAPDEAFMDIIRLHRPAIASRRGLVRYSRIGQRGSMHTRSGKHIGARIHYFYDISPIA---QAA-----EEIEMHPL--------------------VAAQDDTF----DIYAESFEPGINPTQH---------PVTNI   367
[HPV74]gil27462490lgblAAO15461.1lAF436130_7  LQFQHNTI--HNPPDDAFMDIIRLHRPAITSRRGLVRFSRIGQRGSMHTRSGKHIGGRVHFFQDISPISAAESAA-----EEIELHPL---VAH--------------AQDSSGLF----DIYAEPDLE------------VMEEPVPLS   372
HPV7gil9627394lreflNP_041858.1l              LHFEQPSI--HNAPDPAFMDIITLHRPALTSRRGVVRFSRVGQRGTMYTRRGTRIGGRVHFFKDISPI----ASS-----EEIELHPL---VA---------------SPNNSDLF----DVYADIDDI--DENIL------YSTIDNNT   372
Hpv7gi|397066|emb|CAA52480.1|                LHFEQPSI--HNAPDPAFMDIITLHRPALTSRRGVVRFSRVGQRGTMYTRRGTRIGGRVHFFKDISPI----ASS-----EEIELHPL---VA---------------SPNNSDLF----DVYADIDDI--DENIL------YSTIDNNT   372
[HPV40]                                      LQFEQPSI--HDAPDPAFMDIITLHRPALTSRRGVIRFSRVGQRGTMYTRRGTRIGGRVHFFRDISPI----GAA-----DDIELHPL---VASAPHTLETPHTLETPLDTTDALF----DVYADMDTIDDDAAYA--------------   381
[HPV43]                                      LTFEQPTV--HEAPDSRFMDIVTLHRPALTSRRGIVRFSRVGARGTMYTRSGIRIGGRVHFFTDISSI----PTE-----ESIELQPL---GRSQSFP---------TVSDTSDLY----DIYADENLLNNDISFT--------------   373
[Hpv91]                                      LAFEQPTV--HEAPDSNFMDIVTLHRPALTSRRGLVRFSRVGARGTMYTRSGIRIGGRVHFFKDISPI----ATQ-----ESIELQPL---GHSTV-----------NVPDATDLY----DIYADDSIL--DVPHT--------------   369
[HPV54]                                      LHFEQPTI--HNAPDPAFMDIFALHRPALTTRRGVVRYSRVGDRATLHTRSGLQLKPRVHFFQDLSPIA---HVP-----EEIELHPL---ISANN------------TSINNGLYSDIYDVYADTDFA--DTGGF-------SSSTVSH   379
[HPV32]gi|9627332|ref|NP_041805.1|           LEFEHPTI--HEAPDSDFMDIIALHRPVLSARQGTVRVSRIGQRASLQTRSGARIGSRVHFFHDISPIT---RPS-----EAIELQPL---GSSST-----AVSTTASSAINDGLF----DVYVDPDIP-----------PSHALPPLRS   384
[HPV32]gi|396987|emb|CAA52553.1|             LEFEHPTI--HEAPDSDFMDIIALHRPVLSARQGTVRVSRIGQRASLQTRSGARIGSRVHFFHDISPIT---RPS-----EAIELQPL---GSSST-----AVSTTASSAINDGLF----DVYVDPDIP-----------PSHALPPLRS   384
[HPV4]2gi|333218|gb|AAA47047.1|              LVFEHPSI--HTAPDPDFMDIVALHRPMLSSKQGSVRVSRIGQRLSMQTRRGTRFGSRVHFFHDLSPIT---HSS-----ETIELQPL---SASSV---------SAASNINDGLF----DIYVDTSDV--NVTNTTSSIPMHGFATPRL   389
HPV34_L2aa                                   LEFQHSDL--HNAPDSDFLDIVKLHRPALTARKTGIRVSRLGQRATMFTRSGKRIGGRVHFYHDLSPI-----PT-----ENIELQPL---LPSAS------ATVTDANGINDGLY----DVLLDNNVD------------ITEVETPTG   382
HPV34gi|9627339|ref|NP_041811.1|             LEFQHSDL--HNAPDSDFLDIVKLHRPALTARKTGIRVSRLGQRATMFTRSGKRIGGRVHFYHDLSPI-----PT-----ENIELQPL---LPSAS------ATVTDANGINDGLY----DVLLDNNVD------------ITEVETPTG   382
HPV34gi|396995|emb|CAA52559.1|               LEFQHSDL--HNAPDSDFLDIVKLHRPALTARKTGIRVSRLGQRATMFTRSGKRIGGRVHFYHDLSPI-----PT-----ENIELQPL---LPSAS------ATVTDANGINDGLY----DVLLDNNVD------------ITEVETPTG   382
[HPV73]                                      LEFQHSDL--HNAPDSDFLDIVKLHRPALTSRKTGIRVSRLGQRATLSTRSGKRIGAKVHFYHDISPI-----PT-----NDIEMQPL---VTPQT------PSIVTGSSINDGLY----DVFLDNDVE--ETVLQ------QTYTPTSI   386
RMPV_L2aa                                    LQFSHSDI--HQPPDPDFLDIVALHRPALTSRKGTVRFSRLGQRATLTTRSGKRIGAKVHFYHDLSPI----APA-----ESIELQPL---------------------SSQGELY----DIYADVDGQEDAAAVA--------------   374
HPV52_L2aa                                   IIFDRSQL--LPAPDPDFLDIIALHRPALTSRRGTVRFSRLGNKATLRTRSGKQIGARVHYYHDISPI----QPAEVQ--EDIELQPL---LPQSV----------SPYTINDGLY----DVYADSLQQ----------------PTFHL   374
HPV67_L2aa                                   LYFQHQDI--SPAPDPDFLDIVALHRPALTSRKGTIRFSRLGSKATMKTRSGKQIGARVHYYQDLSPI----VPA-----DSIELQPLSRPVSSAS------------HSINDGLY----DVYMDPD------------------TPFPQ   370
HPV33_L2aa                                   LQFQHSDI--SPAPDPDFLDIIALHRPAITSRRHTVRFSRVGQKATLKTRSGKQIGARIHYYQDLSPIV---PLDHTVPNEQYELQPL---HDTST----------SSYSINDGLY----DVYADDVDN--------------VHTPMQH   379
HPV58_L2aa                                   LQFQHSDI--SPAPDPDFLDIVALHRPALTSRRGTVRYSRVGQKATLRTRSGKQIGAKVHYYQDLSPIQPVQEQVQQQ--QQFELQSLNTSVSP--------------YSINDGLY----DIYADDADT-----------------IHDF   376
[HPV35]                                      LQFEHEDI--SLAPDPDFMDIIALHRPALTSRKGTIRYSRVGNKRTMHTRSGKAIGARVHYYQDLSSIT-----------EDIELQPL-QHVPSSL------PHTTVSTSLNDGMF----DIYAPIDTE--EDIIF------SASSNNTL   382
HPV31_L2aa                                   LYFSNTSH--NIAPDPDFLDIIALHRPALTSRRNTVRYSRLGNKQTLRTRSGATIGARVHYYYDISSI----NPAG----ESIEMQPL---GASAT----------TTSTLNDGLY----DIYADTDFTVDTPATH-----------NVS   372
[HPV16EAvar]                                 LYFPNNDNSINIAPDPDFLDIVALHRPALTSRRTGIRYSRIGNKQTLRTRSGKSIGAKVRYYYDLSTI----DPA-----EEIELQTI---TPSTY---TTTSHAASPTSINNGLY----DIYADDFIT-----------DTFTTPVPSV   385
HPV16_L2aa                                   LYFSSNDNSINIAPDPDFLDIVALHRPALTSRRTGIRYSRIGNKQTLRTRSGKSIGAKVHYYYDLSTI----DPA-----EEIELQTI---TPSTY---TTTSHAASPTSINNGLY----DIYADDFIT--DTSTT---------PVPSV   385
[Hpv16NP_041331.1]                           LYFSSNDNSINIAPDPDFLDIVALHRPALTSRRTGIRYSRIGNKQTLRTRSGKSIGAKVHYYYDLSTI----DPA-----EEIELQTI---TPSTY---TTTSHAASPTSINNGLY----DIYADDFIT--DTSTT---------PVPSV   385
[HPV16EGvar]                                 LYFPSNDNSINIAPDPDFLDIVALHRPALTSRRTGIRYSRIGNKQTLRTRSGKSIGAKVHYYYDLSTI----DPA-----EEIELQTI---TPSTY---TTTLHAASPTSINNGLY----DIYADDFIT--DTSTT---------PVPSV   385
[HPV16AAvar]                                 FYFPSNDNSINIAPDPDFLDIVALHRPALTSRRTGIRYSRIGNKQTLRTRSGKSIGAKVHYYYDLSTI----NPA-----EEIELQTI---TPSTY---TTTSHAASPTSINNGLY----DIYADDFIT--DTVTT---------PVPAI   385
[HPV16Af2]                                   LYFASNDNSINIAPDPDFLDIVALHRPALTSRRTGIRYSRIGNKQTLRTRSGKSIGAKVHYYYDLSTI----NPA-----EEIELQTI---TPSTY---TTPSHAASPTSINNGLY----DIYADDFIT-----------DTFTTPVPSI   385
[HPV16Af1]                                   LYFPSNDNSINIAPDPDFLDIVALHRPALTSRRTGIRYSRIGNKQTLRTRSGKSIGAKVHYYYDLSTI----NPA-----EEIELQTI---TPSTY---TTASHAASPTSINNGLY----DIYADDFIT--DTSTT---------PVPSI   385
                                             .......310.......320.......330.......340.......350.......360.......370.......380.......390.......400.......410.......420.......430.......440.......450
```

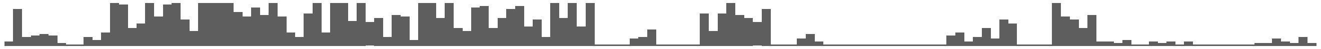

CLUSTAL 2.0.5 MULTIPLE SEQUENCE ALIGNMENT

File: /Users/johnlowe/Desktop/L2muscle.ps **Date: Fri May 2 13:22:30 2008**


```
                                                                    . : *:                    .                                 : .
[HPVcand89]                                  SGP----TVTAT-SAVSSQ--YTNVTVPLSQGFD------VPAVT---GPDIAFPTAPSVPPIFP---------------SYTLGPSTGYI-----YSRYGFLFSTQLCVFS 456
[HPV81]                                      VSG----SATLSATSVASS---YNTTVPLSSGFT------IPAST---GPDVELPYAPLAPSIVPSFPATTPYAIYVVGTDFYLFPSYIFFPKK--HKRIHYSFTDGYVAAW 472
[HPV61]                                      TVQ----GPSLSAASASTK--VHNVTVPLATGLD------TPVTS---GPDVDFAHAPAPVPAVPYVPATHPHSIYIQGSDFYLLPAYVFFPKR--RKRVPYSFSDGFVAAW 459
[HPV72]                                      HSP----SLSAA-SAVSAK--YDNVTVPLSLGPH------IPASS---GPDIDLSFAPAPVPTMPLVPSTHPHSIYVEGFDFYLLPAYIFFPKR--RKRVPYSFADGFVAAW 466
[HPV83]                                      QST----NVSAT-SAVTAQ--YDNVTVPLSPGLD------VPALS---GPDIHGAAHMPADPSVPVPSNIPIQSVFIDGTDYYLLPNYIFFPKK--RKRVHYSFADGYVAAW 471
[HPV84]                                      PFA----SADLSATSITAST-YDNVTVPLFSGTD------VPVYT---GPDIDHSAAPSAPPFVPVIPSTTPYAIYILGSDYYLLPNYIFFPKK--RKRVPYSFSDGFVAAW 474
[HPV86]                                      TTPVSSATVSAT-SMVSST--YDNVTVPLSSATE------VPLYT---GPDIDHAIAPSPTPLPPVVPSTTPYAIYIQGSDYYLLPNYIFFPRK--RKRVHYSFSDGFVAAW 478
[HPVcand87]                                  PTA----STKVSATSILSSSHYDNVTVPL-SATD------VPLYT---GPDIDHSMAPSPTPFVPVVPSTAPYSVYIQGSDYYLLPNYIFFPKR--RKRVPYSFSDGFVAAW 472
[HPV71]                                      PSV------TAT-SAVSASR-TQNVTAPLSAGAD------VPVFD---GPDIDFSTSHATTPTPVVPSIAPPSSFIVYGTEVLFNA-------------------------- 446
[HPV90]                                      PSV------TAS-SALSSA--ASNTTVPLSTGLD------IPVFS---GPDSALPDSHAVWPVPPAPPGVVPGSVLVNGSTYYLLPPLGLLPKK--RKRFPYFFADGNVEA- 467
[HPV10]gil9627262lreflNP_041745.1l           YNT----TSPLS-STLSTK--YGNVTIPFVSPVD------VTLHT---GPDIVLPTS-AQWPYVPLSPADTTHYVYIDGGDFYLWPVTFHFSRHRRRKRVSYFFADGTLAL- 470
[Hpv10]gil396907lemblCAA52493.1l             YNT----TSPLS-STLSTK--YGNVTIPFVSPVD------VTLHT---GPDIVLPTS-AQWPYVPLSPADTTHYVYIDGGDFYLWPVTFHFSRHRRRKRVSYFFADGTLAL- 470
[Hpv94]                                      YST----ASPLS-STLSTK--FGNVTIPFVSPVD------VTLHT---GPDIILPTS-AQWPYVPLTPADTTHYVYIDGGDFYLWPVTFYLSRRRRRKRV------------ 459
[Hpv3]                                       RAT----VSPLS-STLSTK--YGNVTIPFVSPVD------VPLQP---GPDILLPAS-AQWPFVPLSPVDTTHYVYIDGGDFYLWPVTFFLPRRRRRKRVSYFLADGTVAL- 473
hpv28                                        YTT----VSPLS-STLTTK--YGNVTIPFVSPVD------VHLHP---GPDIITPAS-TQWPFVPLVPADTTHYVYIDGGDFYLWPVTLFVPRRRRRKRLSYFLADGTVAL- 473
hpv29                                        RIT----PSVFS-STLSTR--YGNVTIPFVSPVD------VPLHT---GPDIILPSS-AQWPFVPVAPADTTHYVYIDGGDYFLWPVTFPVSRKRRRKRLSYFLADGFVAL- 473
[Hpv77]                                      RGA----SSSLP-SALSTK--IGNVTIPFISPVD------VHLHT---GPDIVLPSS-AQWPFVPVLPADTTHYVYIDGGNFYLWPVTFSVSRKRRRKRLSYFFADGTVAL- 473
[HPV56]                                      PST----------LSFASN--TTNVTAPLGNVWE------TPFYS---GPDIVLPTGPSTWPFVPQSPYDVTHDVYIQGSSFALWPVYFFRRRR--RKRIPYFFADGDVAA- 464
[hpv66]gi|1020296|gb|AAA79504.1|             PST----------LSFASN--TANVTAPLGNVWE------TPFYS---GPDIVLPTGPSTWPFVPQSPSDVTHDVYIQGATFALWPVYFFKRRR--RKRIPYFFADGDVAA- 464
[HPV53]gi|9627381|ref|NP_041847.1|           PTN----TVPLS---FSGS--TSNVTIPFGTSWD------VPIYS---GPDVVLPTGPPTWPYAPQSPFDTTHDVVIQGSTFALWPVYFLKRRR--RKRIPYFLADGGVAA- 463
[HPV53]gi|397051|emb|CAA52594.1|             PTN----TVPLS---FSGS--TSNVTIPFGTSWD------VPIYS---GPDVVLPTGPPTWPYAPQSPFDTTHDVVIQGSTFALWPVYFLKRRR--RKRIPYFLADGGVAA- 463
[HPV30]                                      PTN----TVPLS---FSSQ--TTNVTIPLGKYWD------VPIYS---GPDIVLPTGPTTWPYAPQAPFDTTHDVVIHGSTFALWPVYFLRRRR--RKHVPYFLADGGVAA- 463
HPVcand85_L2aa                               PTT------STI-SSVSSR--YSNTTIPLATSWD------VPVHT---GPDMTLPTTIPQWPNIVPLLPNNTHSVVLQGTNYYLWPNYYFIFKK--RKRVPYFLTDGFVAF- 467
HPV39_L2aa                                   TTYNTGSLPSVA-SSASTK--YANTTIPFSTSWN------MPVNT---GPDIALPSTTPQLPLVPSGPIDTTYAITIQGSNYYLLPLLYFFLKK--RKRIPYFFSDGYVAV- 470
HPV70_L2aa                                   PRSHL-SFPSIP-STVSTK--YSNTTIPFTTSWD------IPVTT---GPDIVLPTASPNLPFVPPTSIDTTVAIAIQGSNYYLLPLLYYFLKK--RKRIPYFFTDGFVAV- 466
HPV59_L2aa                                   SFQ----SLSLT-RSASST--FSNVTVPLATAWD------VPVNT---GPDIVLPNTNIVEPTYSTTPFTTIQSINIEGTNYFLWPIYYFLPRK--RKRVPYFFTDGSMAF- 464
HPV18_NP_040316.1                            FKY----SPTIS-SASS----YSNVTVPLTSSWD------VPVYT---GPDITLPSTTSVWPIVSPTAPASTQYIGIHGTHYYLWPLYYFIPKK--RKRVPYFFADGFVAA- 462
HPV18_CAA28670.1                             FKY----SPTIS-SASS----YSNVTVPLTSSWD------VPVYT---GPDITLPSTTSVWPIVSPTAPASTQYIGIHGTHYYLWPLYYFIPKK--RKRVPYFFADGFVAA- 462
HPV45_L2aa                                   PKY----SLTMP-STAASS--YSNVTVPLTSAWD------VPIYT---GPDIILPSHTPMWPSTSPTNASTTTYIGIHGTQYYLWPWYYYFPKK--RKRIPYFFADGFVAA- 463
[HPV82]                                      PFSPLSTQLPSLSSSVSSS--YANVTIPFSTTYN------VPIHT---GPDVVLPTSPTVWPFIPHTSIDTQHAIVIQGGDYYFVAVYIFVTQT------P----------- 459
[HPV69]                                      PRF----SSTIF-STTSSA--PLNVTIPLSTSFD------IPIYN---GPDIYAPVPSSTWPYIPPPPTTMSHSVVAQGGNYYLWPYIYLIHKR-RRKRVPCFFSDGLAAY- 467
[HPV26]                                      PRY----ASNVF-SSINTS--TTNVTVPLSTSFE------LPVYS---GSDIYTPTSSPTWPSLPPPPTTNLPAIVVHGDNYYLWPYIYLIHKR--RKRMPYFFSDGFVAY- 472
[HPV26b_CAA52534.1]                          PRY----ASNVF-SSINTS--TTNVTVPLSTSFE------LPVYS---GSDIYTPTSSPTWPSLPPPPTTNLPAIVVHGDNYYLWPYIYLIHKR--RKRMPYFFSDGFVAY- 471
Pcpv1_L2aa                                   PSS----TSFVRSSMFTTK--WGNTTVPLSLPSN------IFAQP---GPDIIFPAAPGVPPYNPVIPSLPITPIFISGSQFYLHPSLYLARKR--RKRVSLFFAD--VAA- 463
[ccpv1]                                      NST----LFIKS-SIFDTS--WGNTTIPLSLPNN------IFVQP---GPDILFPTTPAVPPYGPVISPLPVGPVFISGSEFYLHPSLYFARKR--RKRVSLFFSD--VAA- 462
[HPV6b]gil9626061lreflNP_040303.1l           SDT----YLTSTPNTVTQP--WGNTTVPLSLPND------LFLQS---GPDITFPTAPMGTPFSPVTPALPTGPVFITGSGFYLHPAWYFARKR--RKRIPLFFSD--VAA- 459
[HPV74]gil27462490lgblAAO15461.1lAF436130_7  FST----STPFQRSAVSAT--WGNTTVPLSLPGD------MFIQP---GPDIMFPTASSTTPYSPVTPALPTGPVFINGSTFYLYPSWYFARKR--RKHVPLFFTD--VAA- 464
HPV7gil9627394lreflNP_041858.1l              PTS----TYSLY-PGNSTR--IANTSIPLATIPD------TFLTS---GPDIVFPSVPAGTPYLPVSPSIPAISVLIRGTDYYLNPAYYFRKRR--KRILAY---------- 456
Hpv7gi|397066|emb|CAA52480.1|                PTS----TYSLY-PGNSTR--IANTSIPLATIPD------TFLTS---GPDIVFPSVPAGTPYLPVSPSIPAISVLIRGTDYYLNPAYYFRKRR--KRILAY---------- 456
[HPV40]                                      -------TFSLH-PADSTR--ISNTSIPLATVSD------TLLTS---GPDIVFPSIPAGTPYLPVSPSIPAISVLIHGTDYYLHPAYY-LRKR--RKRI---LAHQYVAT- 467
[HPV43]                                      -------DTHVS-LQNSTK--VVNTAVPLATVPD------IYAQT---GPDISFPTIPIHIPYIPVSPSISPQSVSIHGTDFYLHPSLWHLGKR--RKRFSYFFTDNYVAA- 463
[Hpv91]                                      -------NTNVSTLPNTTK--IVNTTVPLATVPD------IYTQA---GPDITFPTVPLSVPYVPLSPSVTPSAVSIYGTDFYLHPSLLHFGKR--RKRISYFFADNYVAA- 460
[HPV54]                                      SSV----QTALQTTSIPSQ--YGNTTVPLTAS--------SPYTP---IPTSFRPSS-GHTPFVPARPIFPQTPIAVNGGDFYLHPSYTYVRKR--RKRFPYFLADGYVAA- 470
[HPV32]gi|9627332|ref|NP_041805.1|           PTHVSTVSLTSL-GSVPAQ--TANTTVPLSLPTN------INV-----GPDLSPPESPPFISTRPVSP--SFDSVMVLGWDFILHPSY-MWRKR--RKPVPYFFADVRVAA- 476
[HPV32]gi|396987|emb|CAA52553.1|             PTHVSTVSLTSL-GSVPAQ--TANTTVPLSLPTN------INV-----GPDLSPPESPPFISTRPVSP--SFDSVMVLGWDFILHPSY-MWRKR--RKPVPYFFADVRVAA- 476
[HPV4]2gi|333218|gb|AAA47047.1|              STT----SFPTL-PSMSTH--SANTTIPFSFPAT------VHV-----GPDLSVVDHPWDSTPTSVMP--QGNFVMVSGWDFILHPSYF-WRRR--RKPVPYFFADVRVAA- 477
HPV34_L2aa                                   TNT----QSVFA-SEISTT--TANTTIPLNAGLD--------THP---GPDIALPVPTAETIFTPTVPVQPSGPIYIYGSDFILHPSLYVIPRK--RKRLSYFFAD--VATY 472
HPV34gi|9627339|ref|NP_041811.1|             TNT----QSVFA-SEISTT--TANTTIPLNAGLD--------THP---GPDIALPVPTAETIFTPTVPVQPSGPIYIYGSDFILHPSLYVIPRK--RKRLSYFFAD--VATY 472
HPV34gi|396995|emb|CAA52559.1|               TNT----QSVFA-SEISTT--TANTTIPLNAGLD--------THP---GPDIALPVPTAETIFTPTVPVQPSGPIYIYGSDFILHPSLYVIPRK--RKRLSYFFAD--VATY 472
[HPV73]                                      HSN------SLVSSDISTA--TANTTIPFSTGLD--------THP---GPDIALPLPSTETIFTPIVPLQPAGPIYIYGSGFILHPSYYLLKRK--RKRLSYSFTD--VATY 475
RMPV_L2aa                                    -------NTPLN-SNSSGIASPWNTTVPLSAGAD------VTLQS---GPDVSLDAPVAESPVHPGVPLRPSAHIILYGGDFYLHPSYLGIRRK--RKRMHNFFSDVYVAA- 466
HPV52_L2aa                                   PST------------LSTH--NNTFTVPINSGIDFVYQPTMSIES---GPDIPLPSLPTHTPFVPIAPTAPSTSIIVDGTDFILHPSYFLLRRR--RKRFPYFFTDVRVAA- 466
HPV67_L2aa                                   PSI----SYSLH-SPQT-----TNVTVPLSSGFDFPFSSTVPLQP---GPDIVSPVAPTYTPFVPVIPTSPFNNVLVYGSDFILHPSYF-LRRR--RKRFPYFFADVRVAA- 465
HPV33_L2aa                                   SYS----------TFATTR--TSNVSIPLNTGFD------TPVMS---GPDIPSPLFPTSSPFVPISPFFPFDTIVVDGADFVLHPSYFILRRR--RKRFPYFFTDVRVAA- 467
HPV58_L2aa                                   QSP----LHSHT-SFATTR--TSNVSIPLNTGFD------TPLVSLEPGPDIASSVTSMSSPFIPISPLTPFNTIIVDGADFMLHPSYFILRRR--RKRFPYFFADVRVAA- 472
[HPV35]                                      YTT-----------SNTAYV-PSNTTIPLSSGYD------IPITA---GPDIVFNSNTITNSVLPV-PTGPIYSIIADGGDFYLHPSYYLLKRR--RKAIPYFFADVSVAV- 469
HPV31_L2aa                                   PST----AVQST-SAVSAYV-PTNTTVPLSTGFD------IPIFS---GPDVPIEHAPTQVFPFPLAPTTPQVSIFVDGGDFYLHPSYYMLKRR--RKRVSYFFTDVSVAA- 466
[HPV16EAvar]                                 PST------SLS-GYIP-----ANTTIPFGGAYN------IPLVS---GPDIPINITDQAPSLLPIVPGSPQYTIIADAGDFYLHPSYYMLRKR--RKRLPYFFSDVSLAA- 473
HPV16_L2aa                                   PST------SLS-GYI-----PANTTIPFGGAYN------IPLVS---GPDIPINITDQAPSLIPIVPGSPQYTIIADAGDFYLHPSYYMLRKR--RKRLPYFFSDVSLAA- 473
[Hpv16NP_041331.1]                           PST------SLS-GYIP-----ANTTIPFGGAYN------IPLVS---GPDIPINITDQAPSLIPIVPGSPQYTIIADAGDFYLHPSYYMLRKR--RKRLPYFFSDVSLAA- 473
[HPV16EGvar]                                 PST------SLS-GYIP-----ANTTIPFGGAYN------IPLVS---GPDIPINITDQAPSLIPIVPGSPQYTIIADAGDFYLHPSYYMLRKR--RKRLPYFFSDVSLAA- 473
[HPV16AAvar]                                 PST------SLS-GYIP-----ANTTIPFGGAYN------IPLVS---GPDIPINTTDQTPSLIPIVPGSPQYTIIADGGDFYLHPSYYMLRKR--RKRLPYFFSDVSLAA- 473
[HPV16Af2]                                   PST------SLS-GYIP-----ANTTIPFGGAYN------IPLVS---GPDIPINTTDQTPSLIPIVPGSPQYTIIADGGDFYLHPSYYMLRKR--RKRLPYFFSDVSLAA- 473
[HPV16Af1]                                   PST------SLS-GYIP-----ANTTIPFGGAYN------IPLVS---GPDIPINTTDQTPSLIPIVPGSPQYTIIADGGDFYLHPSYYMLRKR--RKRLPYFFSDVSLAA- 473
                                             .......460.......470.......480.......490.......500.......510.......520.......530.......540.......550.......560..
```

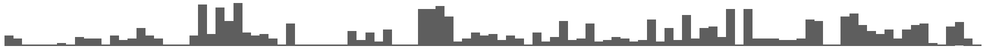

```
HPV16                  1 MRHKRSAKRTKRASATQLYKTCKQAGTCPPDIIPKVEGKTIAEQILQYGS    50
                                                 .....||.:|...|
bpv1                   1                         PAIVTPDAVPADSG------------    14

HPV16                 51 MGVFFGGLGIGTGSGTGGRTGYIPLGTRPPTATDTLAPVRPPLTVDPVGP   100
                             ...|.|||.|                 :|:||..:     ::|.||
bpv1                  15 ----LDALSIGTDS-----------------STETLITL-----LEPEGP    38

HPV16                101 SDPSIVSLVEETSFIDAGAPTSVPSIPPDVSGFSITTSTDTTPAILDINN   150
                         .|.:::.|          .|...|:                    ..::|
bpv1                  39 EDIAVLEL----------QPLDRPT--------------------WQVSN    58

HPV16                151 TVTTVTTHNNPTFTDPSVLQPPTPAETGG--HFTLSSSTISTHNYEEIPM   198
                         .|     |.:..:..|..|| .:.|||.|  :..:..|.:.....|.|.:
bpv1                  59 AV-----HQSSAYHAPLQLQ-SSIAETSGLENIFVGGSGLGDTGGENIEL   102

HPV16                199 DTFIVSTNPNTVTSSTPIPGSRPVARLGLYSRTTQQVKVVDPAFVTTPTK   248
                         ..|   .:|.|   |||
bpv1                 103 TYF---GSPRT---STP                                     113
```
